# An analysis of past and future heatwaves based on a heat-associated mortality threshold: towards a heat health warning system

**DOI:** 10.1186/s12940-022-00921-4

**Published:** 2022-11-19

**Authors:** Thandi Kapwata, Michael T. Gebreslasie, Caradee Y. Wright

**Affiliations:** 1 Environment and Health Research Unit, South African Medical Research Council, Johannesburg, 2028 South Africa; 2 Department of Geography, Geoinformatics and Meteorology, University of Pretoria, Pretoria, 0028 South Africa; 3 School of Agriculture, Earth, and Environmental Sciences, University of KwaZulu-Natal, Durban, 3629 South Africa; 4 Environment and Health Research Unit, South African Medical Research Council, Pretoria, 0084 South Africa

**Keywords:** Heatwave, Diurnal temperature range, Threshold, Mortality

## Abstract

Heatwaves can have severe impacts on human health extending from illness to mortality. These health effects are related to not only the physical phenomenon of heat itself but other characteristics such as frequency, intensity, and duration of heatwaves. Therefore, understanding heatwave characteristics is a crucial step in the development of heat-health warning systems (HHWS) that could prevent or reduce negative heat-related health outcomes. However, there are no South African studies that have quantified heatwaves with a threshold that incorporated a temperature metric based on a health outcome. To fill this gap, this study aimed to assess the spatial and temporal distribution and frequency of past (2014 – 2019) and future (period 2020 – 2039) heatwaves across South Africa. Heatwaves were defined using a threshold for diurnal temperature range (DTR) that was found to have measurable impacts on mortality. In the current climate, inland provinces experienced fewer heatwaves of longer duration and greater intensity compared to coastal provinces that experienced heatwaves of lower intensity. The highest frequency of heatwaves occurred during the austral summer accounting for a total of 150 events out of 270 from 2014 to 2019. The heatwave definition applied in this study also identified severe heatwaves across the country during late 2015 to early 2016 which was during the strongest El Niño event ever recorded to date. Record-breaking global temperatures were reported during this period; the North West province in South Africa was the worst affected experiencing heatwaves ranging from 12 to 77 days. Future climate analysis showed increasing trends in heatwave events with the greatest increases (80%—87%) expected to occur during summer months. The number of heatwaves occurring in cooler seasons is expected to increase with more events projected from the winter months of July and August, onwards. The findings of this study show that the identification of provinces and towns that experience intense, long-lasting heatwaves is crucial to inform development and implementation of targeted heat-health adaptation strategies. These findings could also guide authorities to prioritise vulnerable population groups such as the elderly and children living in high-risk areas likely to be affected by heatwaves.

**Supplementary Information:**

The online version contains supplementary material available at 10.1186/s12940-022-00921-4.

## Background

There is no universally accepted definition of a heatwave. However, heatwaves are typically described as a consecutive period of hot days with temperatures above a given threshold [38]. Increasing evidence shows that climate change induced by global warming will lead to more frequent and more extreme weather events such as heatwaves [21, 57]. Projections of future climates based on the Representative Concentration Pathways (RCP) scenarios of emissions and concentrations of greenhouse gases show that the frequency of heatwaves and heat-related mortality is expected to increase significantly in many parts of the world [11, 12, 45, 47]. These scenarios were presented in the Intergovernmental Panel on Climate Change (IPCC) Sixth Assessment Report (AR6) and are widely used to investigate the effects of climate change [27]. The most commonly used RCP scenarios are 4.5 (intermediate scenario with moderate emissions) and 8.5 (high emission scenario) [43].

Climate change projections for Africa show an increase in the frequency, duration, and intensity of heatwave events, all of which can negatively impact human health, air quality and environmental conditions [4, 14, 39, 66]. The World Meteorological Organization Expert Team on Climate Change Detection (ETCCDI) and the Expert Team on Sector-specific Climate Indices (ET-SCI) developed climate indices to identify and describe temperature and rainfall extremes and heatwave characteristics that are widely used as a tool to assess and monitor changes in weather and climate extremes under a wide variety of climates [2, 54]. The advantage of these indices is that they provide consistent, standardized methodologies which can be applied across the world to study changes in climate and attribute the changes to causes [54]. However, due to the sparse coverage of weather monitoring stations and the lack of long records of daily temperature data, there are very few studies in Africa that investigate climate and the associated influence on heat extremes. In Nigeria, an analysis of four percentile-based threshold indices (warmest day, warmest night, coldest day, coldest night) using data from stations located around the country found statistically significant increases in warm days and nights [1]. The number of days where maximum and minimum temperatures were above the 90^th^ percentile increased at mean annual rates ranging from 1.71 – 1.95 and 1.34 – 2.50 days per decade respectively during the period 1971–2012 resulting in a higher frequency of hot extreme events [1]. Similar patterns were observed in a comprehensive South African study that found statistically significant annual trends with warm days increasing at a rate of 0.15% per year between 1960—2016. Maximum temperature across the country increased at a rate of 0.02 °C per year annually and heatwave events across the country increased by 0.03 events per year during the same time period [61]. Increases in high temperature extremes calculated from in situ measurements have also been reported in the Greater Horn Region of Africa [41].

These studies applied climate indices to access and quantify warming trends for parts of Africa using observed data however modelled future climate conditions also show a high likelihood of significant temperature increases in Africa with estimates of 4 °C – 6 °C over sub-tropical regions and 3 °C – 5 °C over tropical regions [15, 26, 53, 66]. The number of heatwaves is also expected to increase especially over the Sahel and Saharan Africa [15, 63]. South Africa is no exception and simulations of future climate scenarios show that the country could experience 20 to 80 more heatwave days during the period 2017—2100 [14]. The intensity of these heatwaves will also be high, with maximum temperatures calculated to increase by 4 °C to 6 °C across Southern Africa [14]. Furthermore, these events will last longer with durations of up to 10 days with the northern parts of the country being particularly susceptible. [39].

The most severe human impacts of heatwaves relate to morbidity and mortality associated with exposure to extreme heat [6, 68]. Most notably, 70 000 fatalities were reported during a record-breaking heatwave in Europe in 2003, while more recently, 55 000 people died during a heatwave in Russia in 2010 [18, 46,19]. Studies show increased morbidity during heatwaves based on high numbers of emergency department visits, hospitalisations, and ambulance callouts [65,7, 40].

Given the impacts of heat on human health, heatwaves should be defined and monitored by heat thresholds that are associated with negative health outcomes. Several studies around the world have identified country or region-specific heat thresholds beyond which negative health consequences are expected [9], [30, 37, 44]. An examination of the temperature-mortality relationship in seven regions across China found that mortality increased when mean daily temperature exceeded values ranging from 17.7 °C to 27.4 °C [36]. A multi-city epidemiologic study in Europe found that the mortality threshold for heat effects was 29.4 °C for Mediterranean cities while and 23.3 °C for the north European cities [5]. Another comprehensive heat mortality study involved a multi-country analysis of cities in India, Canada, Brazil, Chile, Mexico, Thailand, Slovenia, Romania, and Bulgaria [28]. Results showed that heat thresholds were higher in cities with warmer climates and heat-related mortality increased beyond thresholds ranging from 16.0 °C to 31.0 °C [28].

In South Africa, city-specific apparent temperature (a metric for ‘real-feel temperature’) thresholds of 18.6 °C, 24.8 °C and 18.7 °C, were identified for increased risk of mortality in Cape Town, Durban and Johannesburg, respectively [67]. However, temperature-mortality associations in this study were limited to only three cities therefore monitoring heatwaves using these thresholds would exclude large parts of the overall population. Scovronick et al. [50] estimated the mean daily temperature beyond which mortality increases, also known as Minimum Mortality Temperature (MMT), for 52 district municipalities in South Africa. Mortality was found to increase above mean daily temperatures ranging from 18.5 °C to 39 °C [50]. However, the two cited studies were aimed at identifying single day extreme temperature events. They did not apply thresholds of the different temperature exposure metrics (apparent temperature, maximum temperature, minimum temperature and average temperature) to characterise heatwaves in South Africa.

To the best of our knowledge, there are no South African studies that have quantified heatwaves in the country using thresholds for internationally recognised climate indices beyond which the risk of adverse health outcomes increases. Our study investigates the distribution and characteristics of past and future heatwaves in South Africa by applying a previously determined temperature—mortality threshold for Diurnal Temperature Range (DTR), to define a heat limit. The threshold was identified in a recent South African study that assessed the performance of ETCCDI climate indices to identify the most important predictor of mortality during summer[29].

DTR reflects daily temperature variability and an increasing body of research has shown the adverse health impacts of exposure to high DTR [10, 73]. Additionally, evidence suggests that high DTR (above 90th percentile) increases short-term mortality [73] hence its effectiveness in identifying hot extremes such as heatwaves [32]. An understanding of heatwave patterns is an important component of risk assessment for heat response planning to minimise the impacts of extreme heat. Knowledge of the extent and severity of future heatwaves can guide strategies and policy development for that reduce the heat-related health risks and enhance climate change adaptation.

## Methods

### Data

Maximum and minimum daily temperature data for 2014 – 2019 (inclusive) in South Africa were obtained with permission from the South African Weather Service. Data were available for 50 out of the 52 district municipalities (there were no data for Alfred Nzo district municipality which is in the Eastern Cape province and Amajuba district municipality in KwaZulu- Natal province) in South Africa, hereafter referred to districts. Missing temperature variables accounted for 16% of the data and were excluded from analysis.

### Analysis

#### Past Heatwaves

Previous work by Kapwata (2021) identified that DTR was the most suitable metric for use in a Heat-Health Warning System (HHWS) for South Africa. Furthermore, the study identified a country-specific (nationwide) threshold of DTR 12.8 °C at which alerts should be triggered for heat awareness. All-cause mortality in South Africa was found to increase significantly above this threshold [29]. Thus, the national threshold was therefore used to define the temperature threshold for heatwaves in this study.

The period used to heatwave in this study was set at two days as previous studies have found that heatwave-related mortality risks were highest when the definition used was two or more consecutive days of temperatures exceeding heat thresholds [3, 70]. In Australia, which has a similar sub-tropical climate to South Africa, an investigation into the relationship between heatwaves and health outcomes in Australia, which has a similar sub-tropical climate to South Africa, found that a heatwave duration of two days was most suitable to use to define heatwaves when considering the impact of mortality and morbidity (emergency hospital admissions) [58]. Similar findings were also reported for a multi-country study that found heatwave durations of four days or more did not have higher effect estimates on mortality than shorter durations of two days or more [24]. This was corroborated by another study which found that differences in excess mortality during 2-day and 3-day heatwave events were minor [60], therefore two days is sufficient to model heatwave mortality relationships.

Given the projected increases in global temperature, we also projected data from RCP 4.5 and 8.5 emission scenarios [43] to investigate future characteristics of heatwave events in South Africa during the period 2020 **–** 2039.

Minimum Mortality Temperature (MMT) is an important indicator to assess the temperature–mortality relationship [71]. It represents the mean daily temperature at which the lowest mortality occurs and quantifies the separation between mortality attributed to cold and heat [17]. MMT varies significantly across regions and reflects human adaptability to local climate Therefore, in order to limit heatwave analysis to days when the population would have been most susceptible to heat, district level MMT estimates calculated by [50] were used to create a subset of data that was included in heatwave event analysis. All days with mean temperature below MMT were excluded when defining a heatwave based on the exceedance of the national threshold of DTR of 12.8 oC. This limited the heatwave analysis to days which were associated with the highest mortality risk.

The HeatwaveR package in R was used to detect district level heatwaves based on the exceedance of the national threshold [49]. The input data was a full time series of daily DTR. Heatwave duration was set at two days when running the algorithm and DTR was used as input for the heatwave exposure metric and was calculated as the difference between maximum and minimum temperature. The detection algorithm produces information on the duration of heatwave events (set at two or more consecutive days above the threshold), start of, peak (highest temperature during heatwave) and end dates of heatwaves as well as information on intensity.

#### Future Heatwaves

The global climate model, Coupled Model Intercomparison Project Phase 5 (CMIP5) simulates data for the difference (anomaly) in daily maximum and minimum temperature for the period 2020 – 2039 using Representative Concentration Pathway (RCP) 4.5 and 8.5 emission scenarios. These anomalies were applied to observed data to calculate future daily minimum and maximum temperature. CIMP5 models have a spatial resolution of 1° x 1^o^ produced through bi-linear interpolation. Similar to the analysis of heatwaves using observed data, this dataset also excluded days during which mean temperature was below MMT to estimate future heatwave events.

Stata version 15 [52] was used for descriptive statistics and to calculate the number of ‘hot days’ per district (days during DTR was above country threshold).

## Results

### Past climatic conditions and heatwaves

Descriptive statistics for maximum and minimum temperatures, and DTR for the districts in South Africa from 2014—2019 are presented in Table 1.

**Table 1:** Description of maximum and minimum temperature and calculated DTR in South Africa from 2014 – 2019 (*SD*  Standard deviation, *p95*   95^th^ percentile). See Additional File 1 for the full name of the District code shown in column 1

*District*	*Province*	*Maximum temperature*			*Minimum temperature*			*DTR*		
		mean	SD	p95	mean	SD	p95	mean	SD	p95
*BUF*	Eastern Cape	23.9	4.7	32.3	13.2	4.0	19.4	10.7	4.2	18.3
*CPT*	Western Cape	22.9	5.3	31.9	12.5	4.0	18.6	10.5	4.2	18.3
*DC1*	Western Cape	26	6.6	37.3	10.8	4.6	17.8	15.2	5.6	25
*DC10*	Eastern Cape	24.9	5.9	35.4	10.9	5.0	18.2	14.0	5.8	24.3
*DC12*	Eastern Cape	24.9	6.2	35.5	10.8	4.6	18.0	14.1	5.0	22.7
*DC13*	Eastern Cape	24.2	6.5	35.3	8.8	5.4	16.6	15.5	5.1	23.6
*DC14*	Eastern Cape	22.4	6.3	32.2	5.4	6.3	14.5	17.0	4.9	25.2
*DC15*	Eastern Cape	25	4.5	32.8	13.6	4.5	20.1	11.4	4.8	20.6
*DC16*	Free State	25.8	6.2	35.3	8.4	6.8	17.9	17.4	4.5	24.3
*DC18*	Free State	27.1	5.5	35.4	10.0	6.5	18.6	17.1	4.5	24.2
*DC19*	Free State	23.5	5.1	31.3	7.7	5.7	15.5	15.7	4.8	23.2
*DC2*	Western Cape	25.2	6.6	35.7	10.6	5.1	18.7	14.6	5.0	22.6
*DC20*	Free State	26.3	5.3	34.3	10.2	5.9	17.8	16.0	4.0	21.7
*DC21*	KwaZulu-Natal	23.9	3.5	29.5	15.9	3.5	21.3	8	3.3	14.4
*DC22*	KwaZulu-Natal	24.6	5.6	33.7	9.9	5.7	17.8	14.6	6	24.4
*DC23*	KwaZulu-Natal	24.9	5.7	34	9.6	5.1	17.1	15.3	5	23.2
*DC24*	KwaZulu-Natal	25.6	5.8	34.6	11	5	17.9	14.7	5.4	22.9
*DC26*	KwaZulu-Natal	26.8	5.2	35.1	13.4	4.6	20.4	13.4	4.9	21.5
*DC27*	KwaZulu-Natal	28.6	4.1	35.6	17.2	3.9	22.8	11.3	4	18.4
*DC28*	KwaZulu-Natal	27.8	4.7	35.9	16.5	4.1	22.3	11.3	4.3	18.8
*DC29*	KwaZulu-Natal	27.8	4.9	36	16.3	3.8	21.9	11.5	4.6	19.9
*DC3*	Western Cape	23	5.2	32.8	12.4	4.3	18.7	10.6	5.3	20.5
*DC30*	Mpumalanga	23.9	4.8	31.3	8.5	4.9	15.2	15.4	4.4	22.2
*DC31*	Mpumalanga	23.6	4.7	30.9	9.3	4.8	16	14.3	4.1	20.3
*DC32*	Mpumalanga	26.5	5.5	35.5	13.3	5.3	21.2	13.2	5.5	23.3
*DC33*	Limpopo	28.7	5.1	37.1	15	4.5	21.7	13.7	5.1	21.9
*DC34*	Limpopo	28.1	5.2	36.6	15.1	4.7	21.8	13	5	21.8
*DC35*	Limpopo	26	4.4	33.1	12	4.8	18.6	14	4	20
*DC36*	Limpopo	29.8	4.8	37.4	13.6	6.1	21.9	16.2	4.7	23.7
*DC37*	North West	28.9	5.2	36.8	12.1	6.1	20.2	16.9	4.5	23.6
*DC38*	North West	27.9	5.3	36.2	11	6.2	19.4	17	4.5	23.7
*DC39*	North West	29.6	5.9	38.6	10.1	7	19.7	19.4	4.9	26.6
*DC4*	Western Cape	23.6	5.3	33.4	12.1	4.1	18.5	11.5	4.9	20.7
*DC40*	North West	27.3	5.2	35	10.5	6.1	18.4	16.8	4.5	24
*DC42*	Gauteng	25	4.8	32.3	8.9	6.4	17.4	16.1	4.5	23
*DC43*	KwaZulu-Natal	23.7	5.6	32.5	8.2	5.5	16	15.5	5.8	24.6
*DC45*	Northern Cape	29.4	6.2	39	10.9	6.8	20.7	18.5	4.5	25.3
*DC47*	Limpopo	30	4.4	36.8	12.9	5.6	20.5	17.1	4.2	23.3
*DC48*	Gauteng	25.4	4.8	32.3	9.1	6.1	17.2	16.3	4.6	23.5
*DC5*	Western Cape	26.7	7	37.8	11.1	5.8	19.8	15.7	4.5	22.8
*DC6*	Northern Cape	25.3	6.8	36.2	10.0	5.9	18.8	15.3	5.4	24.1
*DC7*	Northern Cape	27	7.1	37.6	10.4	6.7	20.6	16.6	3.9	22.8
*DC8*	Northern Cape	31.1	6.8	41.2	12.1	7.1	22.4	19	4.5	26.3
*DC9*	Northern Cape	28.4	6.1	37.5	10.2	6.8	19.9	18.2	4.5	25.1
*EKU*	Gauteng	23.8	4.6	30.7	10.1	5.3	17	13.7	4	20.3
*ETH*	KwaZulu-Natal	25.6	3.4	31	16.4	4	22.3	9.2	3.8	16.3
*JHB*	Gauteng	25.7	4.6	32.6	10.9	5	17.9	14.8	3.9	20.2
*MAN*	Free State	26.8	6	35.9	8.3	6.8	17.8	18.5	4.7	25.6
*NMA*	Eastern Cape	23.9	4.5	31.6	12.6	4.4	19.3	11.3	5.1	21.3
*TSH*	Gauteng	26.8	4.9	34.3	12.1	5.2	19.1	14.7	3.9	20.9

Daily mean maximum temperature ranged from 22.3 °C in the Eastern Cape to 31.0 °C inland in the Northern Cape province. Daily mean minimum temperature was lowest in Eastern Cape (5.4 °C) and highest in KwaZulu-Natal (17.2 °C). DTR was lowest in the coastal provinces of KwaZulu-Natal, Western Cape and Eastern Cape compared to the provinces situated inland, i.e., North West, Northern Cape, and Free State. It ranged from 8.0 °C in DC21 in KwaZulu-Natal to 19.4 °C in North West province.

**Fig. 1:**
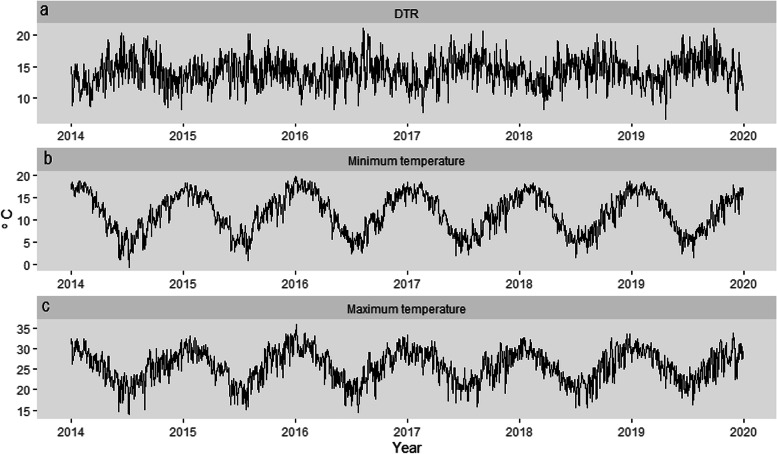
Trends in diurnal temperature range (**a**), daily minimum (**b**) and maximum temperatures (**c**) in South Africa from 2014 – 2019

Figure 1a, b and c present the trends in DTR, minimum and maximum temperatures for South Africa from 2014 to 2019. Maximum and minimum temperatures follow a seasonal pattern peaking during summer months (December, January February) and decreasing during winter (June, July, and August). Maximum and minimum temperatures in summer and winter ranged between 22.4 °C – 36.0 °C and -0.6 °C – 19.6 °C, respectively. The years 2015 and 2016 appeared to have been the warmest during this 6-year period, with the highest mean maximum temperatures reaching 34.0 °C and 36.0 °C, respectively. DTR has been relatively constant across the years, however, the greatest variability was observed in 2014 and 2019. The largest difference between maximum and minimum temperatures occurred during winter and spring (September, October, and November). The highest DTR of 21.1 °C was observed in the spring of 2019.

**Fig. 2:**
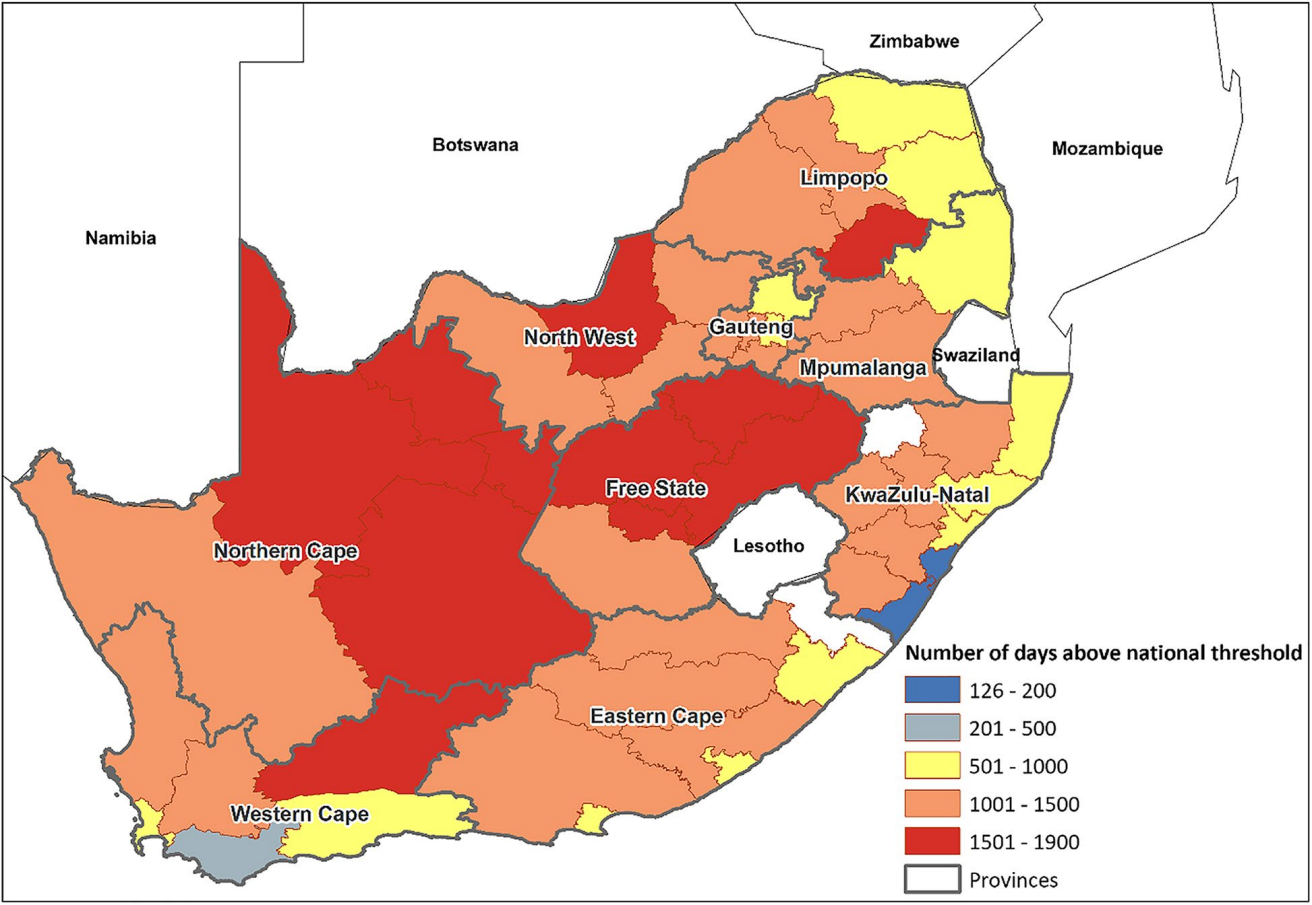
Spatial distribution of the number of days in each of the 50 districts above the national DTR threshold of 12.8 °C over 5 years from 2014–2019 (total n days = 2 191 days)

Figure 2 shows the spatial distribution of the number of ‘hot days’—that being the number of days above the national threshold of DTR above 12.8 °C—across the 50 districts in South Africa over the 6-year period. The spatial variation of DTR that is evident exhibits the difference between coastal and continental climates. Due to cooler conditions, coastal districts experienced a lower prevalence of days during which DTR exceeded the heat threshold. While districts in the warmer interior experienced the highest number of days above the threshold. Districts that experienced the highest number of ‘hot days’ were in the arid regions of the Free State (1 900 days in MAN district) and Northern Cape (1 886 days in DC45). During the study period, four out of five districts in the Northern Cape exceeded the heat threshold between 68.5% and 86.7% of the time while the same percentages of exceedance were observed in four out of five districts in Free State. Figure 2 also emphasises that the risk of extreme heat is not uniform across the country as some parts are more prone to high temperatures than others.

**Fig. 3:**
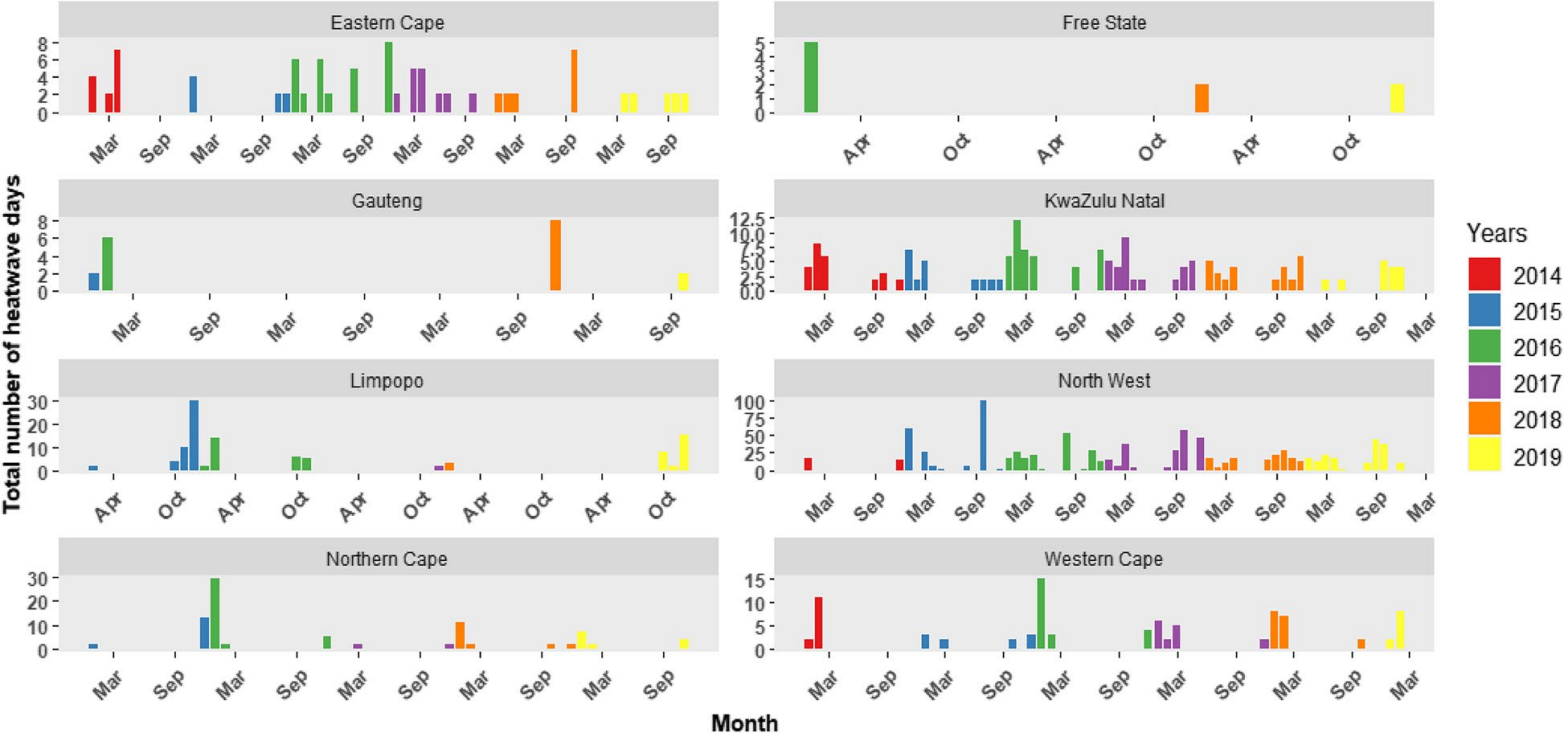
Heatwave events in the nine provinces of South Africa using the national threshold of DTR = 12.8 °C (analysis excludes days during which mean temperature was below mean mortality temperature). Months are shown as 01 for January, 02 for February, 03 for March, 04 April, 05 for May, 06 for June, 07 for July, 08 for August, 09 for September, 10 for October, 11 for November and 12 for December. Years are shown in the colour bars according to the figure legend

The number of heatwave events that occurred in each district during the study period was calculated for days during which mean temperature was above MMT (Fig. 3). A heatwave was defined as ‘the exceedance of DTR of 12.8 °C for 2 days or more’. The occurrence of heatwaves showed spatial, temporal, and seasonal variations. Heatwaves generally occurred more frequently during the summer months. However, coastal provinces (such as Eastern Cape, KwaZulu Natal and Western Cape) experienced heatwaves throughout the year, although these were of low intensity (lower DTR). Inland provinces experienced fewer heatwaves of longer duration, except the North West that had heatwaves during most months of the year. The North -West province had the most intense heatwaves with the longest durations, events ranged from 13 – 77 days and maximum intensity of DTR peaked at 17.9 °C (see Additional File 2). The longest heatwave, using our definition, lasted 77 days from the 29 October 2015 to 13 January 2016. During this time, the population was exposed to more than two months of consecutive days when mean temperature was non-optimum (above MMT) and DTR was above the recommended threshold. Mean and maximum temperatures exceeded 30 °C and 40 °C, respectively. The North West province also had the most extreme heat events accounting for 70 heatwaves out of a total of 270 across the country between 2014 and 2019. Trend analysis showed that 2016 was the hottest year and this is reiterated by the most heatwaves (*n =* 67 events) occurring nationally in that year.

### Future heatwaves based on RCP scenarios

**Fig. 4:**
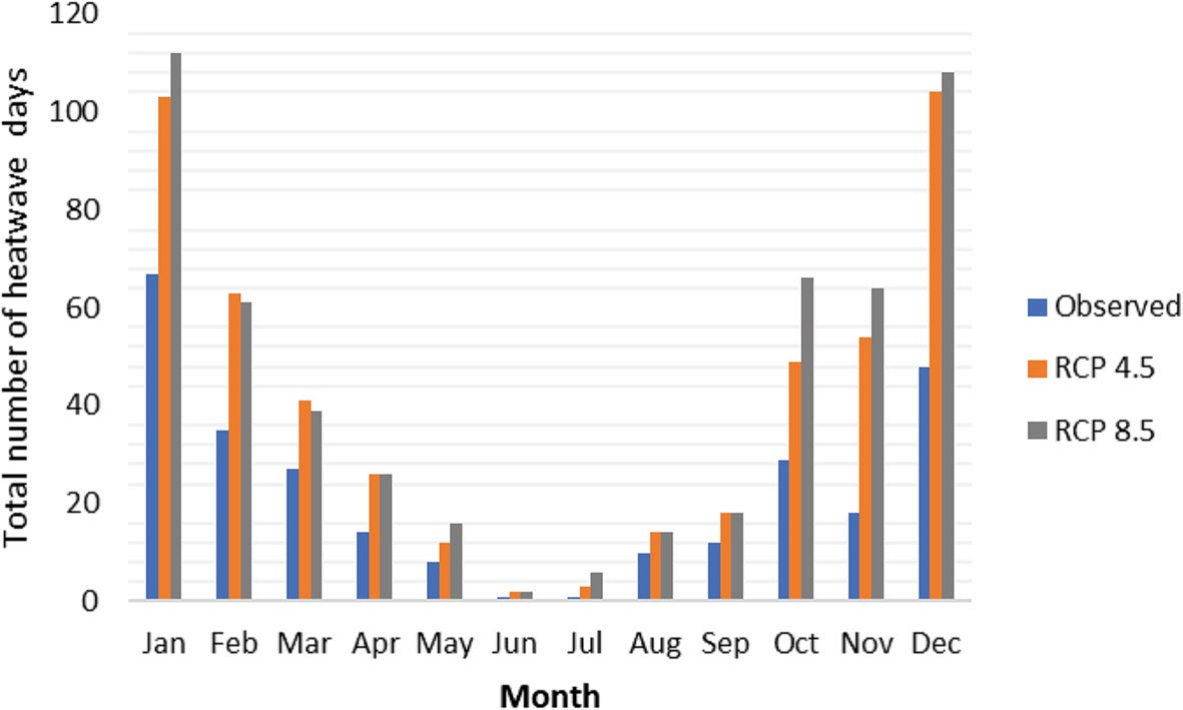
Heatwaves in South Africa under observed climate conditions and Representative Concentration Pathway (RCP) scenarios 4.5 and 8.5

Future projections show that the frequency, duration, and intensity of heatwaves will increase under both the RCP 4.5 and RCP 8.5 scenarios for the period 2020 – 2039 using a health-based metric threshold (see Additional Files 3 and 4). The occurrence of heatwaves is projected to increase throughout the year under both scenarios, however, the greatest increases in heatwave events is estimated to occur during summer (Fig. 4). The total number of heatwaves during December, January and February will increase by up to 80% under the RCP 4.5 scenario across a period of 19 years (270 days compared to 150 under observed climate conditions) and by 87% under RCP 8.5 (281 days). Furthermore, the number of heatwave events starting before summer will increase with more events expected from the winter months of July and August, onwards (Fig. 4). This projected increase in extreme temperature events suggests an increased risk of heat exposure and the associated adverse health outcomes.

**Fig. 5:**
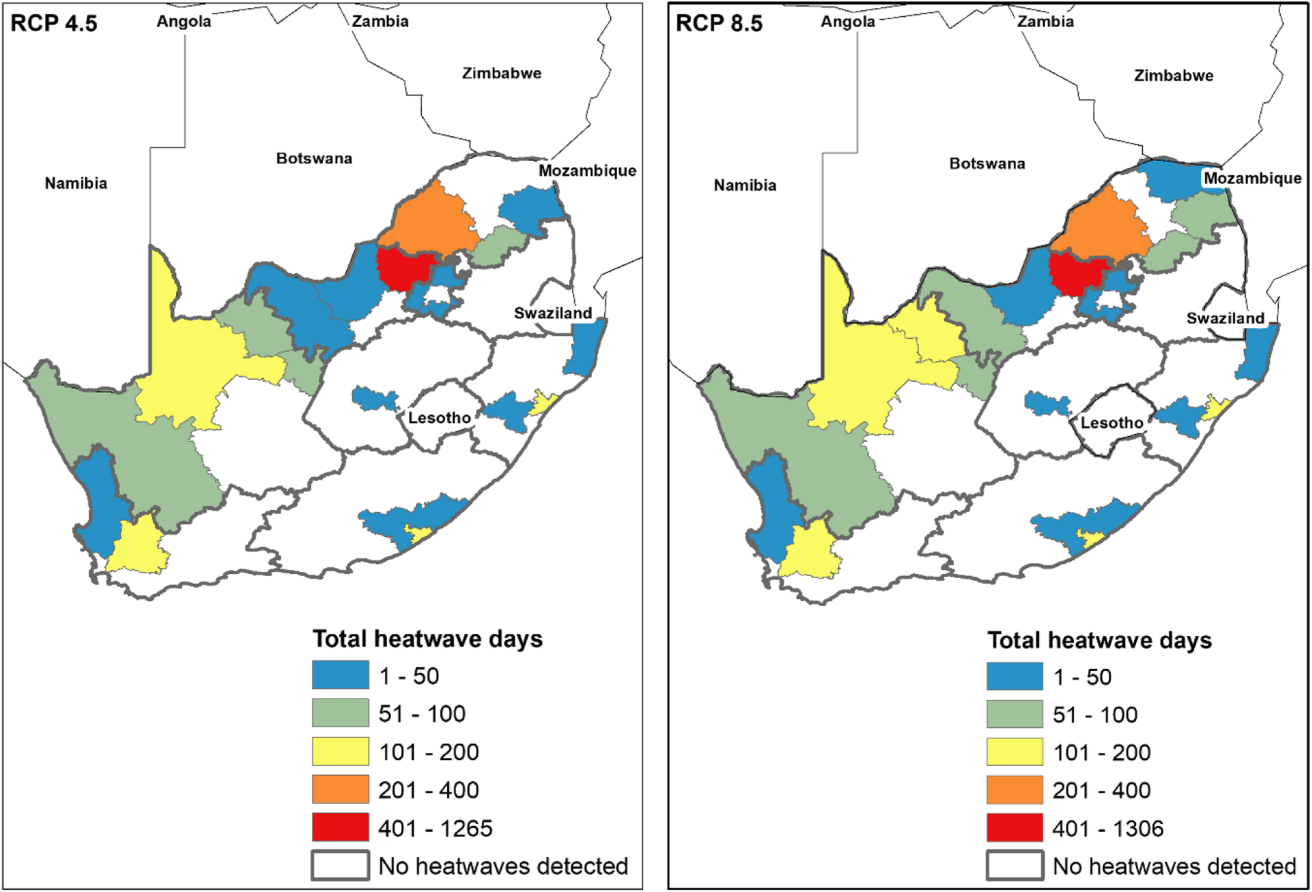
Simulated heatwaves in South Africa during the period 2020 – 2039 (19 years) under Representative Concentration Pathway (RCP) scenarios 4.5 and 8.5

Spatial analysis of the projected district level heatwave data showed that the greatest increases in heatwave days are predicted to occur inland across the north eastern and north western parts of the country (Fig. 5). The geographic extent of heatwaves is slightly wider under RCP 8.5 compared to RCP 4.5 with heatwaves of higher intensity, frequency and duration expected to occur across the districts under the higher emission scenario. Bojanala district in the North West province will experience an increased prevalence of heatwaves in comparison to the other districts under both RCP scenarios; results show more than double the number of heatwaves compared to those predicted in other districts (see Additional Files 3 and 4). This district is also predicted to experience longer-lasting heatwaves over the 19-year future period under both RCP 4.5 and 8.5 scenarios. Coastal districts will experience more heatwaves of lower intensity during the period 2020 – 2039 under both scenarios.

## Discussion

This study set out to explore the distribution and characteristics of past and future heatwaves by applying a newly defined heat-associated mortality threshold to define extreme heat for South Africa. To the best of our knowledge, this is the first study that uses a temperature metric informed by a health outcome (namely, mortality) to assess heatwave characteristics in Africa with the intention to contribute towards a HHWS. Given the rising trends of heatwaves [39, 45], evaluating their frequency, intensity and prevalence enables countries to develop and implement effective heatwave risk assessments by identifying regions that are most vulnerable to these extreme events. This information can also assist countries to enhance heat management and adaptation strategies that strengthen HHWS to minimize the health risks associated with exposure to heatwaves. The rising trends of heatwaves.

We used DTR as an exposure metric to define heatwaves because it was the most significant predictor of mortality in a previous study [29] and it’s applicability in heat-mortality associations has been established in several studies [23, 44, 51, 62, 73, 74]. During the development of HHWS for France, several biometeorological indicators were tested to determine their effect on excess mortality and a combination of minimum and maximum temperature which is suggestive of DTR had the highest performance in models [44]. Studies conducted in North America, Asia and Europe have found statistically significant associations between DTR and mortality [23, 44, 51, 62, 73, 74].

In Shanghai, China, a 1 °C increase in DTR corresponded to a 2.5% [95%CI: 1.76%—3.16%] increase in mortality caused by coronary heart disease adjusted for mean temperature, relative humidity, and air pollution [8]. Similar associations were reported in the USA where risk of non-accidental mortality increased by 0.27% [95% CI: 0.24% – 0.30%] for every 1 °C increase in DTR across 95 communities [35]. DTR was also found to be significantly associated with all-cause mortality (0.66% increased change with 1 °C increase in DTR; [95% CI: 0.26 – 1.06] and respiratory mortality [2.23%; 95% CI: 0.54 – 3.95] in Tokyo, Japan [31]. This evidence coupled with projections showing notable increases in DTR in South Africa in the future [34] and suggests the importance of DTR in heat studies.

Our findings support the idea that continentality is one of the main factors affecting DTR (Jury, 2016, Schulze, 1997), because coastal regions experienced lower DTR compared to inland regions. Furthermore, coastal provinces in South Africa experienced heatwaves throughout the year, however, these were of low intensity compared to those experienced by inland provinces. Inland provinces experienced fewer heatwaves of longer duration and greater intensity with DTR peaking at 17.9 °C in the North West. The national spatial variability of heatwave duration, frequency and intensity could be attributed to the cooling effect that the sea breeze and associated marine layer clouds have on coastal region [20]. Overall, the highest frequency of heatwaves occurred during the austral summer accounting for a total of 150 heatwave events out of 270 events that occurred during 2014 to 2019 (5-year period). These findings are corroborated by Mbokodo et al., 2020 [39] who found most heatwaves (that were defined by temperature thresholds not linked to a health outcome) were experienced during summer and the highest frequency was in the northern regions of South Africa. The findings of our study are consistent with an IPPC report that predicted an increase in the length, frequency and/or intensity of heatwaves over most inland areas based on model projections of temperature [16].

The intensity and duration of heatwaves occurring inland in South Africa could pose an increased risk of heat-related mortality. A systematic review of global studies that assessed mortality risks associated with heatwaves reported that when using a heatwave definition of mean temperatures ≥ 95^th^ percentile for ≥ 2 days, heatwave-related mortality increased by 4% [70]. A related study conducted in Australia found that the higher the intensity and the longer the duration of a heatwave, the greater the health impacts resulting in increased mortality and emergency hospital admissions [59].

In our study, the longest heatwave event that was detected lasted 77 days from the 29 October 2015 to 13 January 2016 over the North West province. This could have been the result of record-breaking temperatures caused by one of the strongest El Niño events ever recorded, occurring during 2015/2016 [55]. During this event, global surface temperatures and the surface air temperature over South Africa reached record highs with maximum temperatures exceeding 40 °C [72,13]. Anomalies of maximum temperature up to 6 °C higher than normal were reported in various parts of the country during typical spring months of October and November 2015, and summer months of December 2015, January and February 2016, and the autumn month of March 2016 [13]. This heatwave had a substantial effect on health, with several media reports made about hospitalisations due to heat-related illness and even death due to heat stroke in the North West province [56]. The detection of this significant heatwave in part substantiates the use of the DTR threshold applied in this study and the 2-day period used to define the duration.

Future climate shows increasing trends in heatwave events with, the greatest increases are expected to occur during summer under both RCP scenarios. Inland provinces such as the North West will experience the largest increase in heatwaves. Similar findings were reported by Mbokodo et al. [39] who found that north western parts of South Africa are predicted to have the highest increase in heatwaves. However, the number of expected heatwave events could have been under-represented because the threshold used was based only on exceedance of regional average mean maximum temperature while the threshold in our study was determined by temperature effect on mortality. 

Our findings suggest that the number of heatwave events starting before summer- months in future climate scenarios is expected to increase with more events expected from the winter months of July and August, onwards. Evidence suggests that risk of heat-related mortality is higher when heatwaves occur ealier in the year, for example, in spring or early summer [22,42]. This is because people are more vulnerable to heat stress earlier in the year before summer as they are not yet acclimitized to high temperatures [25].Therefore, the increase in duration and intensity of heatwaves earlier in the year in South Africa under both RCP sceanrios poses a significant health risk. The projected increase of heatwaves along the coast is concerning because previous studies have found that coastal populations are more susceptibe to heat—coastal districts will be more vulnerable to the adverse impacts of heatwaves in the immediate future due to the possible lower tolerance of coastal residents to heat [48].

A limitation of the study is that while stationary thresholds might perform well in defining past heatwaves applying this threshold to projected data could lead to an overestimation of future heatwave events [33, 64]. Another limitation is the short time span covered by the observed data, since ideally this data should extend over at least 30 years [69]. Also, the longest lasting heatwave event of 77 days in North West province could appear to be an overestimation, or considered ‘days of extreme heat’, however, the definition of a heatwave applied in this study is based on mortality risk associated with exceedance of a temperature metric threshold. Therefore, the population in that province was exposed to an increased risk of possible heat-associated mortality for over two months.

## Conclusion

This study used a mortality risk-based metric to define heatwaves. Continued research into the frequency, intensity and duration of heatwaves is important for the implementation of HHWSs to reduce heat-related mortality and morbidity. It is also necessary to understand the spatial and temporal distribution of heatwaves so that heat warnings can inform interventions and be targeted appropriately. Findings from this study can be used by the South African Weather Service, policy makers and emergency services to prioritise their actions and focus heat prevention strategies on the districts which are vulnerable to heatwaves and where populations are at high risk of being exposed to negative heat related outcomes. The identification of these heatwave ‘hot spots’ will also allow authorities to focus on vulnerable population groups such as the elderly and children living in districts prone to heatwaves. Furthermore, identifying changes in attributes of future heatwaves across the country could guide mitigation and adaptation strategies and policy development.

## Supplementary Information

**Additional** **file 1.** Full names of codes used for districts.

**Additional** **file 2.** Heatwave characteristics from 2014 - 2019 as defined using threshold of 12.8 ^o^C and duration of two days or more.

**Additional** **file 3.** Heatwave characteristics for period 2020 – 2039 using data simulated from RCP 4.5 projections.

**Additional** **file 4.** Heatwave characteristics for period 2020 – 2039 using data simulated from RCP 8.5 projections.

## Data Availability

The data used in this study belongs to the South African Weather Service and can therefore not be made publicly available. Permission was granted for the data to be applied to the current study.
