# Supplementary material for: An analysis of past and future heatwaves based on a heat-associated mortality threshold: towards a heat health warning system
Source: Environ Health. 2022 Nov 19;21:112. doi: 10.1186/s12940-022-00921-4 (PMC9675182; doi:10.1186/s12940-022-00921-4)
Supplement: Supplementary file 1 — Additional file 1. Full names of codes used for districts. [file 12940_2022_921_MOESM1_ESM.docx]

Additional File 1: Full names of codes used for districts

| **District Code** | **District Name** | **District Province** |
| --- | --- | --- |
| DC37 | Bojanala | North West |
| BUF | Buffalo City | Eastern Cape |
| DC29 | iLembe | KwaZulu-Natal |
| DC2 | Cape Winelands | Western Cape |
| DC48 | West Rand | Gauteng |
| DC22 | Umgungundlovu | KwaZulu-Natal |
| DC6 | Namakwa | Northern Cape |
| DC25 | Amajuba | KwaZulu-Natal |
| DC42 | Sedibeng | Gauteng |
| DC43 | Sisonke | KwaZulu-Natal |
| DC27 | Umkhanyakude | KwaZulu-Natal |
| DC1 | West Coast | Western Cape |
| DC12 | Amathole | Eastern Cape |
| DC36 | Waterberg | Limpopo |
| MAN | Mangaung | Free State |
| DC23 | Uthukela | KwaZulu-Natal |
| TSH | City of Tshwane | Gauteng |
| DC44 | Alfred Nzo | Eastern Cape |
| DC38 | Ngaka Modiri Molema | North West |
| EKU | Ekurhuleni | Gauteng |
| DC30 | Gert Sibande | Mpumalanga |
| DC47 | Greater Sekhukhune | Limpopo |
| DC24 | Umzinyathi | KwaZulu-Natal |
| DC33 | Mopani | Limpopo |
| DC9 | Frances Baard | Northern Cape |
| DC45 | John Taolo Gaetsewe | Northern Cape |
| DC3 | Overberg | Western Cape |
| DC39 | Dr Ruth Segomotsi Mompati | North West |
| DC4 | Eden | Western Cape |
| DC20 | Fezile Dabi | Free State |
| NMA | Nelson Mandela Bay | Eastern Cape |
| DC8 | Siyanda | Northern Cape |
| DC31 | Nkangala | Mpumalanga |
| DC15 | O.R.Tambo | Eastern Cape |
| JHB | City of Johannesburg | Gauteng |
| DC19 | Thabo Mofutsanyane | Free State |
| DC13 | Chris Hani | Eastern Cape |
| DC26 | Zululand | KwaZulu-Natal |
| DC34 | Vhembe | Limpopo |
| DC40 | Dr Kenneth Kaunda | North West |
| DC14 | Joe Gqabi | Eastern Cape |
| DC21 | Ugu | KwaZulu-Natal |
| DC32 | Ehlanzeni | Mpumalanga |
| DC18 | Lejweleputswa | Free State |
| DC5 | Central Karoo | Western Cape |
| DC7 | Pixley ka Seme | Northern Cape |
| ETH | eThekwini | KwaZulu-Natal |
| DC10 | Cacadu | Eastern Cape |
| DC16 | Xhariep | Free State |
| DC35 | Capricorn | Limpopo |
| CPT | City of Cape Town | Western Cape |
| DC28 | Uthungulu | KwaZulu-Natal |
