# Supplementary material for: An analysis of past and future heatwaves based on a heat-associated mortality threshold: towards a heat health warning system
Source: Environ Health. 2022 Nov 19;21:112. doi: 10.1186/s12940-022-00921-4 (PMC9675182; doi:10.1186/s12940-022-00921-4)
Supplement: Supplementary file 2 — Additional file 2. Heatwave characteristics from 2014 - 2019 as defined using threshold of 12.8 oC and duration of two days or more. [file 12940_2022_921_MOESM2_ESM.docx]

Additional File 2: Heatwave characteristics from 2014 - 2019 as defined using threshold of 12.8 ^o^C and duration of two days or more

| Duration (days) | Date_start | Date_peak | Date_end | Intensity_mean | Intensity_max | District | Province |
| --- | --- | --- | --- | --- | --- | --- | --- |
| 21 | 9/13/2018 | 9/15/2018 | 10/3/2018 | 9.101 | 17.93 | DC37 | North West |
| 15 | 9/10/2019 | 9/13/2019 | 9/24/2019 | 9.7493 | 14.03 | DC37 | North West |
| 29 | 9/6/2017 | 9/14/2017 | 10/4/2017 | 7.109 | 13.13 | DC37 | North West |
| 26 | 9/28/2019 | 9/28/2019 | 10/23/2019 | 7.3235 | 12.46 | DC37 | North West |
| 3 | 8/28/2019 | 8/28/2019 | 8/30/2019 | 9.3667 | 11.3 | DC37 | North West |
| 3 | 3/20/2017 | 3/20/2017 | 3/22/2017 | 7.4533 | 11.04 | DC2 | Western Cape |
| 12 | 2/25/2019 | 3/1/2019 | 3/8/2019 | 5.5858 | 11.03 | DC37 | North West |
| 77 | 10/29/2015 | 11/9/2015 | 1/13/2016 | 4.0266 | 10.96 | DC37 | North West |
| 2 | 5/13/2015 | 5/14/2015 | 5/14/2015 | 7.56 | 10.96 | DC37 | North West |
| 16 | 3/19/2016 | 3/29/2016 | 4/3/2016 | 4.5656 | 10.93 | DC37 | North West |
| 34 | 12/23/2017 | 1/1/2018 | 1/25/2018 | 4.1991 | 10.86 | DC37 | North West |
| 2 | 10/1/2018 | 10/1/2018 | 10/2/2018 | 7.7 | 10.8 | DC29 | KwaZulu- Natal |
| 3 | 12/18/2015 | 12/18/2015 | 12/20/2015 | 7.5033 | 10.55 | DC8 | Northern Cape |
| 3 | 8/15/2019 | 8/17/2019 | 8/17/2019 | 8.3967 | 10.53 | DC37 | North West |
| 2 | 10/27/2015 | 10/27/2015 | 10/28/2015 | 9.08 | 10.52 | DC2 | Western Cape |
| 14 | 4/14/2018 | 4/24/2018 | 4/27/2018 | 3.8586 | 10.5 | DC37 | North West |
| 2 | 1/10/2018 | 1/11/2018 | 1/11/2018 | 9.095 | 10.42 | DC2 | Western Cape |
| 2 | 1/3/2018 | 1/3/2018 | 1/4/2018 | 9.53 | 10.35 | DC2 | Western Cape |
| 2 | 10/12/2015 | 10/13/2015 | 10/13/2015 | 8 | 10 | DC29 | KwaZulu- Natal |
| 2 | 11/6/2015 | 11/6/2015 | 11/7/2015 | 5.15 | 10 | DC29 | KwaZulu- Natal |
| 19 | 10/24/2018 | 10/26/2018 | 11/11/2018 | 5.3763 | 9.96 | DC37 | North West |
| 3 | 8/15/2018 | 8/15/2018 | 8/17/2018 | 9.5167 | 9.83 | DC37 | North West |
| 2 | 9/4/2019 | 9/5/2019 | 9/5/2019 | 9.315 | 9.83 | DC37 | North West |
| 2 | 1/6/2016 | 1/7/2016 | 1/7/2016 | 8.55 | 9.8 | DC47 | Limpopo |
| 2 | 2/6/2019 | 2/7/2019 | 2/7/2019 | 8.06 | 9.75 | DC2 | Western Cape |
| 3 | 11/11/2015 | 11/11/2015 | 11/13/2015 | 7.1 | 9.7 | DC47 | Limpopo |
| 10 | 12/11/2019 | 12/16/2019 | 12/20/2019 | 3.276 | 9.66 | DC37 | North West |
| 23 | 10/2/2015 | 10/3/2015 | 10/24/2015 | 5.693 | 9.56 | DC37 | North West |
| 2 | 8/20/2016 | 8/21/2016 | 8/21/2016 | 7.395 | 9.53 | DC37 | North West |
| 2 | 3/2/2015 | 3/3/2015 | 3/3/2015 | 8.045 | 9.5 | DC1 | Western Cape |
| 2 | 12/18/2016 | 12/18/2016 | 12/19/2016 | 7.43 | 9.43 | DC8 | Northern Cape |
| 2 | 1/17/2017 | 1/18/2017 | 1/18/2017 | 7.875 | 9.41 | DC2 | Western Cape |
| 2 | 12/16/2018 | 12/16/2018 | 12/17/2018 | 5.58 | 9.38 | DC8 | Northern Cape |
| 36 | 3/6/2017 | 4/1/2017 | 4/10/2017 | 3.4467 | 9.3 | DC37 | North West |
| 4 | 10/9/2018 | 10/11/2018 | 10/12/2018 | 7.455 | 9.13 | DC37 | North West |
| 2 | 12/6/2015 | 12/6/2015 | 12/7/2015 | 6.18 | 9.13 | DC45 | Northern Cape |
| 2 | 5/4/2019 | 5/4/2019 | 5/5/2019 | 8.545 | 9.06 | DC37 | North West |
| 21 | 3/13/2019 | 3/21/2019 | 4/2/2019 | 4.6933 | 9.03 | DC37 | North West |
| 6 | 8/20/2015 | 8/25/2015 | 8/25/2015 | 6.4283 | 8.9 | DC37 | North West |
| 3 | 1/6/2018 | 1/6/2018 | 1/8/2018 | 7.22 | 8.9 | DC36 | Limpopo |
| 2 | 1/22/2017 | 1/22/2017 | 1/23/2017 | 6.71 | 8.8 | DC2 | Western Cape |
| 10 | 1/14/2019 | 1/22/2019 | 1/23/2019 | 3.261 | 8.73 | DC37 | North West |
| 4 | 12/18/2015 | 12/18/2015 | 12/21/2015 | 7.08 | 8.56 | DC45 | Northern Cape |
| 4 | 8/6/2019 | 8/8/2019 | 8/9/2019 | 7.2125 | 8.46 | DC37 | North West |
| 2 | 10/30/2016 | 10/30/2016 | 10/31/2016 | 6.95 | 8.4 | DC47 | Limpopo |
| 5 | 11/9/2015 | 11/9/2015 | 11/13/2015 | 6.176 | 8.38 | DC36 | Limpopo |
| 3 | 8/30/2017 | 8/31/2017 | 9/1/2017 | 6.65 | 8.36 | DC37 | North West |
| 5 | 10/16/2018 | 10/16/2018 | 10/20/2018 | 5.776 | 8.3 | DC37 | North West |
| 18 | 1/9/2014 | 1/18/2014 | 1/26/2014 | 3.8578 | 8.26 | DC37 | North West |
| 2 | 12/29/2016 | 12/30/2016 | 12/30/2016 | 8.16 | 8.25 | DC2 | Western Cape |
| 3 | 10/26/2019 | 10/28/2019 | 10/28/2019 | 4.2733 | 8.23 | DC33 | Limpopo |
| 6 | 4/25/2015 | 4/27/2015 | 4/30/2015 | 6.5233 | 8.13 | DC37 | North West |
| 2 | 10/31/2015 | 10/31/2015 | 11/1/2015 | 7.05 | 8.06 | DC36 | Limpopo |
| 2 | 11/7/2019 | 11/7/2019 | 11/8/2019 | 7.01 | 8.02 | DC36 | Limpopo |
| 2 | 5/19/2019 | 5/19/2019 | 5/20/2019 | 7.75 | 8 | BUF | Eastern Cape |
| 2 | 1/21/2014 | 1/22/2014 | 1/22/2014 | 6.45 | 8 | DC2 | Western Cape |
| 11 | 12/9/2017 | 12/14/2017 | 12/19/2017 | 3.1764 | 7.9 | DC37 | North West |
| 4 | 1/11/2018 | 1/13/2018 | 1/14/2018 | 6.39 | 7.8 | DC8 | Northern Cape |
| 5 | 1/1/2014 | 1/2/2014 | 1/5/2014 | 3.652 | 7.7 | DC37 | North West |
| 10 | 1/15/2017 | 1/19/2017 | 1/24/2017 | 2.7 | 7.66 | DC37 | North West |
| 3 | 12/29/2015 | 12/29/2015 | 12/31/2015 | 4.87 | 7.64 | DC1 | Western Cape |
| 3 | 2/8/2016 | 2/10/2016 | 2/10/2016 | 5.0533 | 7.64 | DC2 | Western Cape |
| 4 | 12/24/2018 | 12/26/2018 | 12/27/2018 | 4.25 | 7.6 | DC48 | Gauteng |
| 3 | 1/24/2015 | 1/24/2015 | 1/26/2015 | 4.7 | 7.6 | DC29 | KwaZulu- Natal |
| 2 | 2/26/2014 | 2/27/2014 | 2/27/2014 | 6.155 | 7.57 | DC2 | Western Cape |
| 5 | 2/13/2016 | 2/16/2016 | 2/17/2016 | 5.18 | 7.5 | DC47 | Limpopo |
| 2 | 3/20/2017 | 3/20/2017 | 3/21/2017 | 6.135 | 7.47 | DC1 | Western Cape |
| 5 | 8/27/2016 | 8/28/2016 | 8/31/2016 | 5.4 | 7.45 | BUF | Eastern Cape |
| 3 | 1/22/2015 | 1/22/2015 | 1/24/2015 | 6.32 | 7.44 | DC2 | Western Cape |
| 2 | 11/28/2019 | 11/28/2019 | 11/29/2019 | 5.08 | 7.43 | DC8 | Northern Cape |
| 2 | 12/17/2019 | 12/18/2019 | 12/18/2019 | 4.25 | 7.4 | DC29 | KwaZulu- Natal |
| 2 | 10/20/2019 | 10/21/2019 | 10/21/2019 | 7.23 | 7.38 | DC36 | Limpopo |
| 2 | 12/13/2016 | 12/14/2016 | 12/14/2016 | 4.995 | 7.34 | DC2 | Western Cape |
| 2 | 10/29/2018 | 10/30/2018 | 10/30/2018 | 3.7 | 7.3 | DC29 | KwaZulu- Natal |
| 3 | 1/29/2016 | 1/29/2016 | 1/31/2016 | 6.7967 | 7.27 | DC2 | Western Cape |
| 2 | 1/5/2016 | 1/5/2016 | 1/6/2016 | 5.17 | 7.27 | DC22 | KwaZulu- Natal |
| 2 | 12/23/2016 | 12/23/2016 | 12/24/2016 | 6.6 | 7.2 | DC29 | KwaZulu-Natal |
| 2 | 10/4/2015 | 10/5/2015 | 10/5/2015 | 6.37 | 7.16 | DC36 | Limpopo |
| 2 | 2/7/2018 | 2/7/2018 | 2/8/2018 | 6.95 | 7.08 | DC2 | Western Cape |
| 2 | 1/5/2018 | 1/6/2018 | 1/6/2018 | 6.89 | 7.03 | DC8 | Northern Cape |
| 4 | 4/23/2018 | 4/26/2018 | 4/26/2018 | 3.4 | 6.9 | DC29 | KwaZulu- Natal |
| 2 | 3/22/2014 | 3/22/2014 | 3/23/2014 | 4.2 | 6.85 | BUF | Eastern Cape |
| 2 | 10/16/2019 | 10/17/2019 | 10/17/2019 | 4.775 | 6.8 | BUF | Eastern Cape |
| 2 | 11/5/2015 | 11/5/2015 | 11/6/2015 | 4.4 | 6.75 | BUF | Eastern Cape |
| 2 | 12/31/2017 | 1/1/2018 | 1/1/2018 | 5.68 | 6.72 | DC36 | Limpopo |
| 5 | 12/20/2015 | 12/22/2015 | 12/24/2015 | 4.436 | 6.7 | DC36 | Limpopo |
| 2 | 12/6/2018 | 12/7/2018 | 12/7/2018 | 4.5 | 6.7 | DC29 | KwaZulu- Natal |
| 2 | 11/28/2019 | 11/28/2019 | 11/29/2019 | 4.865 | 6.63 | DC45 | Northern Cape |
| 4 | 12/15/2018 | 12/18/2018 | 12/18/2018 | 3.075 | 6.6 | DC29 | KwaZulu-Natal |
| 3 | 1/22/2017 | 1/23/2017 | 1/24/2017 | 3.7667 | 6.6 | DC29 | KwaZulu- Natal |
| 2 | 1/4/2016 | 1/5/2016 | 1/5/2016 | 4.25 | 6.6 | DC29 | KwaZulu- Natal |
| 2 | 1/3/2016 | 1/3/2016 | 1/4/2016 | 6.16 | 6.6 | DC2 | Western Cape |
| 2 | 1/27/2019 | 1/28/2019 | 1/28/2019 | 4.455 | 6.6 | DC2 | Western Cape |
| 2 | 1/28/2018 | 1/29/2018 | 1/29/2018 | 6.01 | 6.58 | DC2 | Western Cape |
| 2 | 5/11/2016 | 5/12/2016 | 5/12/2016 | 5.43 | 6.56 | DC37 | North West |
| 8 | 12/4/2014 | 12/5/2014 | 12/11/2014 | 1.9787 | 6.53 | DC37 | North West |
| 2 | 1/31/2016 | 1/31/2016 | 2/1/2016 | 5.8 | 6.5 | DC8 | Northern Cape |
| 26 | 2/11/2016 | 2/13/2016 | 3/7/2016 | 3.3727 | 6.4 | DC37 | North West |
| 8 | 3/9/2018 | 3/15/2018 | 3/16/2018 | 3.6888 | 6.4 | DC37 | North West |
| 3 | 4/17/2017 | 4/19/2017 | 4/19/2017 | 5 | 6.4 | DC37 | North West |
| 2 | 10/27/2018 | 10/27/2018 | 10/28/2018 | 5.805 | 6.4 | DC6 | Northern Cape |
| 2 | 1/3/2019 | 1/3/2019 | 1/4/2019 | 6.19 | 6.4 | DC8 | Northern Cape |
| 2 | 3/6/2016 | 3/7/2016 | 3/7/2016 | 5.17 | 6.3 | DC22 | KwaZulu- Natal |
| 2 | 2/21/2018 | 2/22/2018 | 2/22/2018 | 4.025 | 6.3 | DC2 | Western Cape |
| 2 | 12/29/2015 | 12/29/2015 | 12/30/2015 | 4.335 | 6.25 | DC6 | Northern Cape |
| 2 | 2/19/2016 | 2/20/2016 | 2/20/2016 | 3.97 | 6.22 | DC27 | KwaZulu Natal |
| 2 | 12/6/2015 | 12/7/2015 | 12/7/2015 | 5.72 | 6.22 | DC38 | North West |
| 6 | 4/11/2016 | 4/16/2016 | 4/16/2016 | 3.45 | 6.2 | DC29 | KwaZulu Natal |
| 2 | 2/13/2016 | 2/14/2016 | 2/14/2016 | 3.95 | 6.1 | DC29 | KwaZulu- Natal |
| 2 | 3/30/2017 | 3/30/2017 | 3/31/2017 | 3.325 | 6.05 | BUF | Eastern Cape |
| 2 | 3/30/2017 | 3/31/2017 | 3/31/2017 | 3.8 | 6 | DC29 | KwaZulu- Natal |
| 4 | 10/28/2016 | 10/30/2016 | 10/31/2016 | 5.08 | 5.98 | DC36 | Limpopo |
| 3 | 11/29/2016 | 11/29/2016 | 12/1/2016 | 4.4533 | 5.98 | DC36 | Limpopo |
| 2 | 2/6/2019 | 2/6/2019 | 2/7/2019 | 4.97 | 5.98 | DC6 | Northern Cape |
| 2 | 1/10/2017 | 1/10/2017 | 1/11/2017 | 5 | 5.98 | DC2 | Western Cape |
| 2 | 10/22/2016 | 10/22/2016 | 10/23/2016 | 5.53 | 5.96 | DC37 | North West |
| 7 | 12/20/2014 | 12/21/2014 | 12/26/2014 | 1.6214 | 5.9 | DC37 | North West |
| 2 | 11/24/2018 | 11/25/2018 | 11/25/2018 | 4.5 | 5.9 | DC29 | KwaZulu- Natal |
| 3 | 12/19/2016 | 12/21/2016 | 12/21/2016 | 5.6867 | 5.8 | DC45 | Northern Cape |
| 3 | 1/22/2019 | 1/24/2019 | 1/24/2019 | 5.4433 | 5.8 | DC45 | Northern Cape |
| 3 | 12/25/2018 | 12/26/2018 | 12/27/2018 | 3.6733 | 5.8 | DC38 | North West |
| 2 | 11/20/2017 | 11/20/2017 | 11/21/2017 | 4.95 | 5.8 | DC29 | KwaZulu- Natal |
| 2 | 12/6/2017 | 12/6/2017 | 12/7/2017 | 5.425 | 5.77 | DC1 | Western Cape |
| 7 | 10/23/2018 | 10/24/2018 | 10/29/2018 | 1.9429 | 5.75 | BUF | Eastern Cape |
| 4 | 1/30/2016 | 2/1/2016 | 2/2/2016 | 4.14 | 5.68 | DC6 | Northern Cape |
| 3 | 10/28/2014 | 10/30/2014 | 10/30/2014 | 4.4333 | 5.6 | DC29 | KwaZulu- Natal |
| 3 | 12/25/2019 | 12/25/2019 | 12/27/2019 | 5.0867 | 5.6 | DC33 | Limpopo |
| 2 | 12/6/2015 | 12/7/2015 | 12/7/2015 | 4.75 | 5.6 | DC47 | Limpopo |
| 2 | 2/12/2016 | 2/12/2016 | 2/13/2016 | 3.225 | 5.6 | DC9 | Northern Cape |
| 5 | 4/25/2019 | 4/27/2019 | 4/29/2019 | 3.852 | 5.5 | DC37 | North West |
| 4 | 1/4/2016 | 1/7/2016 | 1/7/2016 | 3.575 | 5.5 | DC48 | Gauteng |
| 5 | 1/3/2016 | 1/5/2016 | 1/7/2016 | 2.648 | 5.46 | MAN | Free State |
| 2 | 1/17/2018 | 1/18/2018 | 1/18/2018 | 4.01 | 5.38 | DC2 | Western Cape |
| 2 | 1/6/2016 | 1/6/2016 | 1/7/2016 | 4.9 | 5.3 | DC42 | Gauteng |
| 2 | 12/23/2015 | 12/24/2015 | 12/24/2015 | 3.6 | 5.3 | DC47 | Limpopo |
| 2 | 1/8/2015 | 1/9/2015 | 1/9/2015 | 4.88 | 5.26 | DC45 | Northern Cape |
| 2 | 3/26/2016 | 3/27/2016 | 3/27/2016 | 3.9 | 5.2 | DC29 | KwaZulu- Natal |
| 2 | 4/4/2017 | 4/4/2017 | 4/5/2017 | 2.65 | 5.2 | DC29 | KwaZulu- Natal |
| 3 | 4/6/2018 | 4/6/2018 | 4/8/2018 | 4.53 | 5.16 | DC37 | North West |
| 2 | 1/14/2018 | 1/14/2018 | 1/15/2018 | 4.23 | 5.16 | DC45 | Northern Cape |
| 6 | 2/14/2014 | 2/17/2014 | 2/19/2014 | 2.4617 | 5.11 | DC2 | Western Cape |
| 4 | 10/31/2017 | 10/31/2017 | 11/3/2017 | 3.3 | 5.1 | DC29 | KwaZulu- Natal |
| 2 | 4/29/2019 | 4/29/2019 | 4/30/2019 | 4.1 | 5.05 | BUF | Eastern Cape |
| 3 | 10/26/2019 | 10/28/2019 | 10/28/2019 | 3.98 | 5.04 | DC36 | Limpopo |
| 2 | 12/18/2015 | 12/18/2015 | 12/19/2015 | 4.82 | 5.04 | DC22 | KwaZulu- Natal |
| 3 | 1/10/2016 | 1/10/2016 | 1/12/2016 | 2.4667 | 5.02 | DC2 | Western Cape |
| 5 | 2/18/2016 | 2/22/2016 | 2/22/2016 | 3.224 | 4.86 | DC36 | Limpopo |
| 8 | 2/11/2014 | 2/12/2014 | 2/18/2014 | 2.125 | 4.8 | DC29 | KwaZulu- Natal |
| 2 | 2/1/2017 | 2/2/2017 | 2/2/2017 | 2.65 | 4.8 | DC29 | KwaZulu- Natal |
| 2 | 2/11/2015 | 2/11/2015 | 2/12/2015 | 3.22 | 4.72 | DC36 | Limpopo |
| 3 | 11/12/2017 | 11/13/2017 | 11/14/2017 | 2.3 | 4.7 | DC29 | KwaZulu- Natal |
| 2 | 10/10/2018 | 10/11/2018 | 10/11/2018 | 3.35 | 4.7 | DC29 | KwaZulu- Natal |
| 2 | 12/23/2019 | 12/24/2019 | 12/24/2019 | 2.85 | 4.7 | DC29 | KwaZulu-Natal |
| 2 | 2/19/2016 | 2/20/2016 | 2/20/2016 | 3.6 | 4.7 | DC33 | Limpopo |
| 2 | 2/22/2017 | 2/23/2017 | 2/23/2017 | 3.81 | 4.68 | DC2 | Western Cape |
| 3 | 1/29/2019 | 1/31/2019 | 1/31/2019 | 2.9633 | 4.6 | DC37 | North West |
| 2 | 9/21/2017 | 9/22/2017 | 9/22/2017 | 3.5 | 4.6 | DC29 | KwaZulu- Natal |
| 8 | 11/3/2016 | 11/10/2016 | 11/10/2016 | 1.2087 | 4.53 | DC37 | North West |
| 4 | 2/28/2018 | 3/3/2018 | 3/3/2018 | 2.8125 | 4.53 | DC37 | North West |
| 2 | 2/1/2016 | 2/1/2016 | 2/2/2016 | 2.275 | 4.5 | DC12 | Eastern Cape |
| 6 | 1/3/2016 | 1/6/2016 | 1/8/2016 | 3.07 | 4.48 | DC39 | North West |
| 2 | 12/1/2015 | 12/2/2015 | 12/2/2015 | 4.06 | 4.48 | DC36 | Limpopo |
| 4 | 1/3/2016 | 1/6/2016 | 1/6/2016 | 2.4625 | 4.45 | DC9 | Northern Cape |
| 3 | 3/5/2016 | 3/7/2016 | 3/7/2016 | 1.8 | 4.4 | DC29 | KwaZulu- Natal |
| 3 | 12/31/2016 | 1/1/2017 | 1/2/2017 | 3.1 | 4.4 | DC29 | KwaZulu- Natal |
| 2 | 6/6/2017 | 6/6/2017 | 6/7/2017 | 3.475 | 4.35 | BUF | Eastern Cape |
| 2 | 3/10/2018 | 3/10/2018 | 3/11/2018 | 3.8 | 4.3 | DC29 | KwaZulu- Natal |
| 7 | 4/8/2014 | 4/14/2014 | 4/14/2014 | 1.6929 | 4.25 | BUF | Eastern Cape |
| 5 | 1/16/2016 | 1/16/2016 | 1/20/2016 | 3.666 | 4.25 | DC2 | Western Cape |
| 7 | 3/17/2017 | 3/18/2017 | 3/23/2017 | 1.2857 | 4.2 | DC29 | KwaZulu- Natal |
| 5 | 1/5/2019 | 1/5/2019 | 1/9/2019 | 1.12 | 4.2 | DC37 | North West |
| 3 | 2/27/2018 | 2/27/2018 | 3/1/2018 | 2.4333 | 4.2 | DC29 | KwaZulu- Natal |
| 2 | 9/23/2014 | 9/23/2014 | 9/24/2014 | 2.2 | 4.2 | DC29 | KwaZulu- Natal |
| 2 | 1/12/2015 | 1/12/2015 | 1/13/2015 | 2.2 | 4.2 | DC29 | KwaZulu- Natal |
| 3 | 2/15/2014 | 2/16/2014 | 2/17/2014 | 2.8133 | 4.19 | DC1 | Western Cape |
| 2 | 2/3/2016 | 2/3/2016 | 2/4/2016 | 4.04 | 4.18 | DC36 | Limpopo |
| 3 | 1/14/2018 | 1/14/2018 | 1/16/2018 | 2.4 | 4.1 | DC9 | Northern Cape |
| 2 | 12/11/2015 | 12/12/2015 | 12/12/2015 | 3 | 4.1 | DC33 | Limpopo |
| 2 | 9/8/2019 | 9/9/2019 | 9/9/2019 | 3.225 | 4.05 | BUF | Eastern Cape |
| 2 | 10/26/2019 | 10/27/2019 | 10/27/2019 | 3.6 | 4 | DC48 | Gauteng |
| 10 | 1/25/2016 | 2/2/2016 | 2/3/2016 | 2.551 | 3.9 | DC37 | North West |
| 2 | 1/23/2014 | 1/24/2014 | 1/24/2014 | 3.55 | 3.8 | DC29 | KwaZulu- Natal |
| 2 | 9/26/2016 | 9/27/2016 | 9/27/2016 | 2.95 | 3.8 | DC29 | KwaZulu- Natal |
| 2 | 11/29/2019 | 11/30/2019 | 11/30/2019 | 2.495 | 3.77 | DC27 | KwaZulu-Natal |
| 2 | 7/6/2017 | 7/6/2017 | 7/7/2017 | 3.325 | 3.7 | BUF | Eastern Cape |
| 2 | 5/5/2017 | 5/5/2017 | 5/6/2017 | 3.5 | 3.7 | DC29 | KwaZulu- Natal |
| 2 | 12/7/2017 | 12/8/2017 | 12/8/2017 | 3.065 | 3.7 | DC6 | Northern Cape |
| 2 | 11/5/2016 | 11/6/2016 | 11/6/2016 | 3.33 | 3.64 | DC36 | Limpopo |
| 2 | 3/1/2017 | 3/1/2017 | 3/2/2017 | 2.115 | 3.63 | DC37 | North West |
| 3 | 1/17/2018 | 1/19/2018 | 1/19/2018 | 2.2 | 3.6 | DC29 | KwaZulu- Natal |
| 2 | 3/31/2014 | 4/1/2014 | 4/1/2014 | 3.45 | 3.6 | DC29 | KwaZulu- Natal |
| 3 | 2/12/2018 | 2/13/2018 | 2/14/2018 | 1.94 | 3.44 | DC2 | Western Cape |
| 6 | 2/19/2016 | 2/24/2016 | 2/24/2016 | 0 | 3.4 | DC29 | KwaZulu- Natal |
| 2 | 10/22/2018 | 10/22/2018 | 10/23/2018 | 2.45 | 3.37 | DC1 | Western Cape |
| 2 | 9/15/2016 | 9/15/2016 | 9/16/2016 | 2.75 | 3.3 | DC29 | KwaZulu- Natal |
| 2 | 1/22/2014 | 1/23/2014 | 1/23/2014 | 1.95 | 3.25 | BUF | Eastern Cape |
| 2 | 1/6/2016 | 1/6/2016 | 1/7/2016 | 2.585 | 3.15 | DC27 | KwaZulu- Natal |
| 2 | 10/31/2017 | 10/31/2017 | 11/1/2017 | 2.1 | 3.1 | BUF | Eastern Cape |
| 2 | 2/12/2018 | 2/12/2018 | 2/13/2018 | 1.835 | 3.09 | DC6 | Northern Cape |
| 2 | 2/8/2018 | 2/9/2018 | 2/9/2018 | 1.85 | 3.05 | BUF | Eastern Cape |
| 4 | 12/31/2016 | 1/1/2017 | 1/3/2017 | 1.8225 | 3 | DC37 | North West |
| 2 | 11/6/2019 | 11/7/2019 | 11/7/2019 | 1.795 | 2.97 | DC27 | KwaZulu- Natal |
| 6 | 4/10/2016 | 4/11/2016 | 4/15/2016 | 0.8417 | 2.95 | BUF | Eastern Cape |
| 2 | 12/17/2016 | 12/18/2016 | 12/18/2016 | 2.4 | 2.95 | BUF | Eastern Cape |
| 2 | 3/20/2017 | 3/20/2017 | 3/21/2017 | 2.885 | 2.95 | DC6 | Northern Cape |
| 2 | 1/16/2019 | 1/17/2019 | 1/17/2019 | 2.695 | 2.93 | MAN | Free State |
| 2 | 1/7/2016 | 1/7/2016 | 1/8/2016 | 1.895 | 2.92 | DC38 | North West |
| 4 | 12/24/2018 | 12/27/2018 | 12/27/2018 | 2.225 | 2.9 | DC42 | Gauteng |
| 2 | 1/22/2019 | 1/23/2019 | 1/23/2019 | 2.25 | 2.75 | DC9 | Northern Cape |
| 3 | 3/21/2017 | 3/22/2017 | 3/23/2017 | 2.3167 | 2.7 | BUF | Eastern Cape |
| 2 | 3/22/2014 | 3/23/2014 | 3/23/2014 | 2.1 | 2.7 | DC29 | KwaZulu- Natal |
| 2 | 9/24/2015 | 9/25/2015 | 9/25/2015 | 1.45 | 2.6 | DC29 | KwaZulu- Natal |
| 2 | 1/12/2018 | 1/12/2018 | 1/13/2018 | 2.6 | 2.6 | DC29 | KwaZulu- Natal |
| 2 | 12/8/2016 | 12/9/2016 | 12/9/2016 | 1.485 | 2.5 | DC27 | KwaZulu- Natal |
| 3 | 2/11/2017 | 2/12/2017 | 2/13/2017 | 1.5767 | 2.4 | DC37 | North West |
| 2 | 1/23/2015 | 1/24/2015 | 1/24/2015 | 1.7 | 2.4 | BUF | Eastern Cape |
| 2 | 12/23/2015 | 12/24/2015 | 12/24/2015 | 2.05 | 2.4 | DC48 | Gauteng |
| 2 | 1/14/2018 | 1/14/2018 | 1/15/2018 | 1.88 | 2.36 | MAN | Free State |
| 2 | 1/27/2014 | 1/27/2014 | 1/28/2014 | 1.575 | 2.35 | BUF | Eastern Cape |
| 2 | 1/16/2016 | 1/17/2016 | 1/17/2016 | 1.8 | 2.3 | DC1 | Western Cape |
| 2 | 2/23/2016 | 2/23/2016 | 2/24/2016 | 1.425 | 2.25 | BUF | Eastern Cape |
| 2 | 12/18/2015 | 12/18/2015 | 12/19/2015 | 1.35 | 2.25 | DC9 | Northern Cape |
| 4 | 1/31/2017 | 2/2/2017 | 2/3/2017 | 0.8575 | 2.2 | DC37 | North West |
| 2 | 12/27/2015 | 12/27/2015 | 12/28/2015 | 2.1 | 2.2 | BUF | Eastern Cape |
| 5 | 4/27/2017 | 5/1/2017 | 5/1/2017 | 1 | 2 | BUF | Eastern Cape |
| 2 | 5/2/2019 | 5/3/2019 | 5/3/2019 | 1.95 | 2 | DC29 | KwaZulu- Natal |
| 2 | 1/29/2016 | 1/29/2016 | 1/30/2016 | 1.6 | 1.85 | BUF | Eastern Cape |
| 2 | 1/4/2015 | 1/5/2015 | 1/5/2015 | 1.225 | 1.75 | DC27 | KwaZulu- Natal |
| 3 | 3/14/2015 | 3/15/2015 | 3/16/2015 | 1.3 | 1.7 | DC29 | KwaZulu- Natal |
| 2 | 1/4/2016 | 1/4/2016 | 1/5/2016 | 1.6 | 1.7 | DC12 | Eastern Cape |
| 2 | 1/15/2015 | 1/16/2015 | 1/16/2015 | 1.525 | 1.6 | BUF | Eastern Cape |
| 2 | 1/11/2018 | 1/11/2018 | 1/12/2018 | 1.475 | 1.55 | BUF | Eastern Cape |
| 2 | 2/14/2016 | 2/15/2016 | 2/15/2016 | 1.285 | 1.5 | DC27 | KwaZulu- Natal |
| 2 | 3/24/2015 | 3/24/2015 | 3/25/2015 | 1 | 1.5 | DC29 | KwaZulu- Natal |
| 2 | 3/14/2014 | 3/15/2014 | 3/15/2014 | 1.15 | 1.4 | DC29 | KwaZulu- Natal |
| 2 | 3/29/2018 | 3/30/2018 | 3/30/2018 | 1.33 | 1.36 | DC37 | North West |
| 2 | 5/31/2016 | 6/1/2016 | 6/1/2016 | 0.825 | 1.35 | BUF | Eastern Cape |
| 2 | 3/27/2018 | 3/28/2018 | 3/28/2018 | 0.95 | 1.25 | BUF | Eastern Cape |
| 2 | 2/11/2017 | 2/11/2017 | 2/12/2017 | 1.095 | 1.12 | DC27 | KwaZulu- Natal |
| 2 | 1/28/2014 | 1/28/2014 | 1/29/2014 | 0.75 | 1.1 | DC29 | KwaZulu- Natal |
| 2 | 2/28/2017 | 3/1/2017 | 3/1/2017 | 0.75 | 1 | DC29 | KwaZulu- Natal |
| 2 | 1/27/2017 | 1/28/2017 | 1/28/2017 | 0.575 | 0.75 | BUF | Eastern Cape |
| 2 | 11/5/2019 | 11/5/2019 | 11/6/2019 | 0.6 | 0.65 | BUF | Eastern Cape |
| 2 | 3/6/2019 | 3/6/2019 | 3/7/2019 | 0.5 | 0.6 | DC29 | KwaZulu- Natal |
| 2 | 2/10/2015 | 2/11/2015 | 2/11/2015 | 0.285 | 0.42 | DC27 | KwaZulu- Natal |
| 2 | 12/20/2014 | 12/20/2014 | 12/21/2014 | 0.21 | 0.27 | DC27 | KwaZulu- Natal |
