# Supplementary material for: An analysis of past and future heatwaves based on a heat-associated mortality threshold: towards a heat health warning system
Source: Environ Health. 2022 Nov 19;21:112. doi: 10.1186/s12940-022-00921-4 (PMC9675182; doi:10.1186/s12940-022-00921-4)
Supplement: Supplementary file 3 — Additional file 3. Heatwave characteristics for period 2020 – 2039 using data simulated from RCP 4.5 projections. [file 12940_2022_921_MOESM3_ESM.docx]

Additional File 3: Heatwave characteristics for period 2020 – 2039 using data simulated from RCP 4.5 projections. NA means not applicable.

| duration (days) | date_start | date_peak | date_end | intensity_mean | intensity_max | District | Province |
| --- | --- | --- | --- | --- | --- | --- | --- |
| 121 | 9/15/2015 | 11/9/2015 | 1/13/2016 | 4.7568 | 11.3272 | DC37 | North West |
| 108 | 9/12/2018 | NA | 12/28/2018 | NA | NA | DC37 | North West |
| 86 | 9/9/2019 | NA | 12/3/2019 | NA | NA | DC37 | North West |
| 79 | 1/2/2015 | 2/7/2015 | 3/21/2015 | 2.7277 | 9.2848 | DC37 | North West |
| 58 | 10/8/2017 | NA | 12/4/2017 | NA | NA | DC37 | North West |
| 56 | 9/7/2014 | NA | 11/1/2014 | NA | NA | DC37 | North West |
| 53 | 8/26/2016 | NA | 10/17/2016 | NA | NA | DC37 | North West |
| 43 | 1/25/2016 | 2/13/2016 | 3/7/2016 | 2.5898 | 6.1848 | DC37 | North West |
| 36 | 3/6/2017 | 4/1/2017 | 4/10/2017 | 3.009 | 8.958 | DC37 | North West |
| 35 | 4/9/2016 | NA | 5/13/2016 | NA | NA | DC37 | North West |
| 34 | 12/23/2017 | 1/1/2018 | 1/25/2018 | 4.3748 | 10.9589 | DC37 | North West |
| 29 | 11/13/2014 | 11/21/2014 | 12/11/2014 | 2.3196 | 8.6105 | DC37 | North West |
| 29 | 10/21/2016 | 10/25/2016 | 11/18/2016 | 3.5012 | 9.2784 | DC37 | North West |
| 29 | 9/6/2017 | 9/14/2017 | 10/4/2017 | 7.2087 | 13.1429 | DC37 | North West |
| 27 | 1/5/2019 | 1/22/2019 | 1/31/2019 | 2.304 | 8.8256 | DC37 | North West |
| 26 | 4/25/2015 | NA | 5/20/2015 | NA | NA | DC37 | North West |
| 23 | 12/17/2015 | NA | 1/8/2016 | NA | NA | DC36 | Limpopo |
| 21 | 3/13/2019 | 3/21/2019 | 4/2/2019 | 4.2319 | 8.5555 | DC37 | North West |
| 20 | 11/22/2016 | 11/29/2016 | 12/11/2016 | 2.7079 | 5.5939 | DC37 | North West |
| 20 | 2/17/2019 | 3/1/2019 | 3/8/2019 | 4.4531 | 10.5555 | DC37 | North West |
| 19 | 8/15/2015 | 8/19/2015 | 9/2/2015 | 7.7148 | 10.3743 | DC37 | North West |
| 18 | 1/9/2014 | 1/18/2014 | 1/26/2014 | 3.9534 | 8.3589 | DC37 | North West |
| 18 | 1/31/2018 | 2/17/2018 | 2/17/2018 | 1.6324 | 5.9514 | DC37 | North West |
| 18 | 12/29/2015 | NA | 1/15/2016 | NA | NA | DC8 | Northern Cape |
| 17 | 2/28/2018 | 3/15/2018 | 3/16/2018 | 2.4141 | 5.9222 | DC37 | North West |
| 16 | 12/28/2015 | NA | 1/12/2016 | NA | NA | DC2 | Western Cape |
| 16 | 3/19/2016 | 3/29/2016 | 4/3/2016 | 4.1164 | 10.4555 | DC37 | North West |
| 16 | 4/19/2018 | 4/24/2018 | 5/4/2018 | 4.9271 | 10.158 | DC37 | North West |
| 15 | 9/30/2015 | NA | 10/14/2015 | NA | NA | DC36 | Limpopo |
| 14 | 1/29/2016 | NA | 2/11/2016 | NA | NA | DC2 | Western Cape |
| 14 | 12/29/2016 | NA | 1/11/2017 | NA | NA | DC2 | Western Cape |
| 14 | 8/13/2018 | NA | 8/26/2018 | NA | NA | DC37 | North West |
| 14 | 8/4/2019 | NA | 8/17/2019 | NA | NA | DC37 | North West |
| 13 | 12/1/2015 | NA | 12/13/2015 | NA | NA | DC36 | Limpopo |
| 13 | 12/19/2019 | NA | 12/31/2019 | NA | NA | DC36 | Limpopo |
| 13 | 4/9/2019 | 4/17/2019 | 4/21/2019 | 2.4324 | 8.6247 | DC37 | North West |
| 13 | 4/25/2019 | 5/4/2019 | 5/7/2019 | 5.3638 | 9.1425 | DC37 | North West |
| 12 | 12/8/2017 | 12/14/2017 | 12/19/2017 | 3.2883 | 8.2966 | DC37 | North West |
| 12 | 1/15/2019 | NA | 1/26/2019 | NA | NA | DC45 | Northern Cape |
| 11 | 2/3/2015 | 2/11/2015 | 2/13/2015 | 2.5425 | 4.5048 | DC36 | Limpopo |
| 11 | 2/12/2016 | 2/22/2016 | 2/22/2016 | 2.7211 | 4.6448 | DC36 | Limpopo |
| 11 | 3/27/2015 | 4/6/2015 | 4/6/2015 | 1.1569 | 5.9914 | DC37 | North West |
| 11 | 4/11/2015 | NA | 4/21/2015 | NA | NA | DC37 | North West |
| 11 | 4/30/2017 | 5/5/2017 | 5/10/2017 | 3.9409 | 5.8759 | DC37 | North West |
| 11 | 12/31/2015 | 1/6/2016 | 1/10/2016 | 5.9631 | 7.4423 | DC39 | North West |
| 11 | 1/13/2014 | NA | 1/23/2014 | NA | NA | DC8 | Northern Cape |
| 10 | 10/19/2019 | NA | 10/28/2019 | NA | NA | DC36 | Limpopo |
| 10 | 2/12/2014 | 2/12/2014 | 2/21/2014 | 1.3648 | 4.3348 | DC37 | North West |
| 10 | 1/15/2017 | 1/19/2017 | 1/24/2017 | 2.7956 | 7.7589 | DC37 | North West |
| 10 | 12/11/2019 | 12/16/2019 | 12/20/2019 | 3.6766 | 10.0633 | DC37 | North West |
| 10 | 1/11/2018 | NA | 1/20/2018 | NA | NA | DC8 | Northern Cape |
| 9 | 2/14/2019 | NA | 2/22/2019 | NA | NA | DC2 | Western Cape |
| 9 | 4/2/2014 | NA | 4/10/2014 | NA | NA | DC37 | North West |
| 9 | 1/2/2016 | 1/5/2016 | 1/10/2016 | 6.1737 | 7.8589 | DC45 | Northern Cape |
| 9 | 11/28/2016 | NA | 12/6/2016 | NA | NA | DC45 | Northern Cape |
| 9 | 2/12/2016 | NA | 2/20/2016 | NA | NA | DC47 | Limpopo |
| 9 | 12/29/2015 | 12/29/2015 | 1/6/2016 | 4.3075 | 6.7066 | DC6 | Northern Cape |
| 9 | 1/7/2015 | NA | 1/15/2015 | NA | NA | DC8 | Northern Cape |
| 8 | 12/30/2015 | NA | 1/6/2016 | NA | NA | DC12 | Eastern Cape |
| 8 | 2/11/2014 | 2/12/2014 | 2/18/2014 | 1.9098 | 4.5848 | DC29 | KwaZulu-Natal |
| 8 | 10/24/2016 | 10/30/2016 | 10/31/2016 | 4.6209 | 6.6084 | DC36 | Limpopo |
| 8 | 12/13/2018 | 12/13/2018 | 12/20/2018 | 3.7247 | 6.4466 | DC36 | Limpopo |
| 8 | 12/16/2016 | 12/20/2016 | 12/23/2016 | 3.5029 | 5.5466 | DC37 | North West |
| 8 | 1/1/2016 | NA | 1/8/2016 | NA | NA | DC38 | North West |
| 8 | 1/1/2019 | NA | 1/8/2019 | NA | NA | DC8 | Northern Cape |
| 8 | 1/3/2016 | NA | 1/10/2016 | NA | NA | DC9 | Northern Cape |
| 7 | 10/23/2018 | 10/24/2018 | 10/29/2018 | 2.5712 | 6.3784 | BUF | Eastern Cape |
| 7 | 1/15/2016 | 1/15/2016 | 1/21/2016 | 3.3048 | 5.8048 | DC1 | Western Cape |
| 7 | 10/22/2018 | NA | 10/28/2018 | NA | NA | DC1 | Western Cape |
| 7 | 4/10/2016 | 4/16/2016 | 4/16/2016 | 2.8295 | 5.858 | DC29 | KwaZulu-Natal |
| 7 | 3/17/2017 | 3/18/2017 | 3/23/2017 | 0.8079 | 3.7222 | DC29 | KwaZulu-Natal |
| 7 | 10/22/2018 | 10/22/2018 | 10/28/2018 | 8.4321 | 11.1141 | DC2 | Western Cape |
| 7 | 12/20/2014 | 12/21/2014 | 12/26/2014 | 2.0204 | 6.2966 | DC37 | North West |
| 7 | 12/31/2016 | 1/1/2017 | 1/6/2017 | 0.8691 | 3.0923 | DC37 | North West |
| 7 | 12/18/2015 | NA | 12/24/2015 | NA | NA | DC48 | Gauteng |
| 7 | 10/24/2015 | NA | 10/30/2015 | NA | NA | DC8 | Northern Cape |
| 7 | 11/30/2015 | NA | 12/6/2015 | NA | NA | DC8 | Northern Cape |
| 6 | 4/10/2016 | 4/11/2016 | 4/15/2016 | 0.4997 | 2.608 | BUF | Eastern Cape |
| 6 | 12/26/2016 | NA | 12/31/2016 | NA | NA | BUF | Eastern Cape |
| 6 | 12/18/2016 | NA | 12/23/2016 | NA | NA | DC22 | KwaZulu-Natal |
| 6 | 8/27/2016 | 8/29/2016 | 9/1/2016 | 6.5524 | 8.3409 | DC29 | KwaZulu-Natal |
| 6 | 10/30/2017 | 10/30/2017 | 11/4/2017 | 3.6165 | 6.6284 | DC29 | KwaZulu-Natal |
| 6 | 1/30/2014 | NA | 2/4/2014 | NA | NA | DC2 | Western Cape |
| 6 | 2/14/2014 | 2/17/2014 | 2/19/2014 | 2.2491 | 4.8991 | DC2 | Western Cape |
| 6 | 12/7/2015 | NA | 12/12/2015 | NA | NA | DC33 | Limpopo |
| 6 | 11/8/2015 | 11/9/2015 | 11/13/2015 | 6.6472 | 8.7405 | DC36 | Limpopo |
| 6 | 4/15/2017 | 4/20/2017 | 4/20/2017 | 4.4025 | 7.558 | DC37 | North West |
| 6 | 3/25/2018 | 3/26/2018 | 3/30/2018 | 2.3416 | 5.4222 | DC37 | North West |
| 6 | 9/1/2018 | 9/4/2018 | 9/6/2018 | 9.4263 | 14.8429 | DC37 | North West |
| 6 | 8/26/2019 | 8/26/2019 | 8/31/2019 | 9.4632 | 13.7076 | DC37 | North West |
| 6 | 12/6/2015 | NA | 12/11/2015 | NA | NA | DC45 | Northern Cape |
| 6 | 12/6/2015 | NA | 12/11/2015 | NA | NA | DC47 | Limpopo |
| 6 | 12/28/2015 | NA | 1/2/2016 | NA | NA | DC47 | Limpopo |
| 5 | 4/10/2014 | 4/14/2014 | 4/14/2014 | 1.858 | 3.908 | BUF | Eastern Cape |
| 5 | 8/27/2016 | 8/28/2016 | 8/31/2016 | 5.7409 | 7.7909 | BUF | Eastern Cape |
| 5 | 12/14/2016 | 12/18/2016 | 12/18/2016 | 0.8266 | 3.3466 | BUF | Eastern Cape |
| 5 | 11/29/2019 | NA | 12/3/2019 | NA | NA | DC27 | KwaZulu-Natal |
| 5 | 4/29/2019 | 5/3/2019 | 5/3/2019 | 0.9887 | 2.0759 | DC29 | KwaZulu-Natal |
| 5 | 10/3/2019 | NA | 10/7/2019 | NA | NA | DC29 | KwaZulu-Natal |
| 5 | 1/20/2015 | 1/20/2015 | 1/24/2015 | 6.4037 | 7.678 | DC2 | Western Cape |
| 5 | 1/16/2016 | 1/16/2016 | 1/20/2016 | 3.7637 | 4.3494 | DC2 | Western Cape |
| 5 | 2/5/2019 | 2/7/2019 | 2/9/2019 | 6.1991 | 9.5419 | DC2 | Western Cape |
| 5 | 12/2/2018 | 12/2/2018 | 12/6/2018 | 5.9616 | 8.9966 | DC36 | Limpopo |
| 5 | 2/27/2019 | 2/27/2019 | 3/3/2019 | 2.4222 | 4.8598 | DC36 | Limpopo |
| 5 | 1/1/2014 | 1/2/2014 | 1/5/2014 | 3.7456 | 7.7923 | DC37 | North West |
| 5 | 11/5/2014 | 11/5/2014 | 11/9/2014 | 4.0705 | 5.2105 | DC37 | North West |
| 5 | 8/29/2017 | 8/31/2017 | 9/2/2017 | 5.8951 | 8.7076 | DC37 | North West |
| 5 | 8/4/2018 | NA | 8/8/2018 | NA | NA | DC37 | North West |
| 5 | 12/23/2018 | 12/26/2018 | 12/27/2018 | 5.3916 | 7.7466 | DC38 | North West |
| 5 | 12/17/2016 | 12/17/2016 | 12/21/2016 | 6.7566 | 8.2966 | DC45 | Northern Cape |
| 5 | 11/9/2015 | 11/9/2015 | 11/13/2015 | 8.5605 | 12.3605 | DC47 | Limpopo |
| 5 | 12/20/2015 | 12/22/2015 | 12/24/2015 | 5.0966 | 6.6966 | DC47 | Limpopo |
| 5 | 12/23/2018 | 12/26/2018 | 12/27/2018 | 4.4366 | 7.9966 | DC48 | Gauteng |
| 5 | 2/14/2014 | 2/15/2014 | 2/18/2014 | 1.347 | 2.5292 | DC6 | Northern Cape |
| 5 | 1/20/2015 | 1/20/2015 | 1/24/2015 | 5.7243 | 6.7323 | DC6 | Northern Cape |
| 5 | 1/29/2016 | 1/29/2016 | 2/2/2016 | 4.5193 | 6.0423 | DC6 | Northern Cape |
| 5 | 1/20/2015 | 1/23/2015 | 1/24/2015 | 5.9423 | 7.6423 | DC8 | Northern Cape |
| 5 | 2/9/2015 | NA | 2/13/2015 | NA | NA | DC9 | Northern Cape |
| 5 | 1/3/2016 | 1/6/2016 | 1/7/2016 | 4.3856 | 7.1989 | MAN | Free State |
| 4 | 4/28/2017 | 5/1/2017 | 5/1/2017 | 1 | 2.0759 | BUF | Eastern Cape |
| 4 | 1/30/2016 | 2/1/2016 | 2/2/2016 | 5.1385 | 7.3848 | DC12 | Eastern Cape |
| 4 | 4/10/2014 | 4/12/2014 | 4/13/2014 | 7.4362 | 8.258 | DC1 | Western Cape |
| 4 | 1/4/2016 | 1/6/2016 | 1/7/2016 | 2.4943 | 3.2423 | DC27 | KwaZulu-Natal |
| 4 | 4/23/2018 | 4/26/2018 | 4/26/2018 | 3.058 | 6.558 | DC29 | KwaZulu-Natal |
| 4 | 12/15/2018 | 12/18/2018 | 12/18/2018 | 3.4716 | 6.9966 | DC29 | KwaZulu-Natal |
| 4 | 1/1/2014 | 1/2/2014 | 1/4/2014 | 4.4815 | 7.5923 | DC2 | Western Cape |
| 4 | 4/10/2014 | 4/12/2014 | 4/13/2014 | 7.8009 | 8.9866 | DC2 | Western Cape |
| 4 | 3/3/2016 | 3/4/2016 | 3/6/2016 | 3.6936 | 6.1793 | DC2 | Western Cape |
| 4 | 2/20/2017 | 2/23/2017 | 2/23/2017 | 2.7884 | 4.4705 | DC2 | Western Cape |
| 4 | 3/19/2017 | 3/20/2017 | 3/22/2017 | 7.0222 | 10.565 | DC2 | Western Cape |
| 4 | 2/12/2018 | 2/13/2018 | 2/15/2018 | 1.4026 | 3.2276 | DC2 | Western Cape |
| 4 | 10/25/2019 | 10/28/2019 | 10/28/2019 | 6.145 | 9.6617 | DC33 | Limpopo |
| 4 | 2/19/2015 | 2/21/2015 | 2/22/2015 | 4.3573 | 6.4648 | DC36 | Limpopo |
| 4 | 11/29/2016 | 11/29/2016 | 12/2/2016 | 4.1836 | 6.3405 | DC36 | Limpopo |
| 4 | 12/24/2018 | 12/27/2018 | 12/27/2018 | 3.7779 | 5.7466 | DC36 | Limpopo |
| 4 | 1/10/2019 | 1/11/2019 | 1/13/2019 | 1.661 | 2.7173 | DC36 | Limpopo |
| 4 | 4/19/2014 | 4/20/2014 | 4/22/2014 | 3.9205 | 4.558 | DC37 | North West |
| 4 | 5/2/2014 | 5/2/2014 | 5/5/2014 | 6.5259 | 8.2259 | DC37 | North West |
| 4 | 9/8/2015 | 9/11/2015 | 9/11/2015 | 6.7096 | 8.1096 | DC37 | North West |
| 4 | 1/22/2019 | 1/24/2019 | 1/25/2019 | 7.9089 | 10.7923 | DC39 | North West |
| 4 | 1/4/2016 | 1/5/2016 | 1/7/2016 | 4.3173 | 5.3923 | DC42 | Gauteng |
| 4 | 12/24/2018 | 12/27/2018 | 12/27/2018 | 2.6216 | 3.2966 | DC42 | Gauteng |
| 4 | 1/7/2015 | 1/7/2015 | 1/10/2015 | 5.0673 | 7.1256 | DC45 | Northern Cape |
| 4 | 2/9/2015 | 2/11/2015 | 2/12/2015 | 5.4931 | 8.8848 | DC45 | Northern Cape |
| 4 | 12/18/2015 | 12/18/2015 | 12/21/2015 | 7.48 | 8.9633 | DC45 | Northern Cape |
| 4 | 12/14/2018 | 12/14/2018 | 12/17/2018 | 5.905 | 9.53 | DC45 | Northern Cape |
| 4 | 10/28/2016 | 10/30/2016 | 10/31/2016 | 7.5034 | 9.0284 | DC47 | Limpopo |
| 4 | 1/4/2016 | 1/7/2016 | 1/7/2016 | 3.6673 | 5.5923 | DC48 | Gauteng |
| 4 | 11/15/2018 | 11/16/2018 | 11/18/2018 | 3.9605 | 5.2605 | DC48 | Gauteng |
| 4 | 3/19/2017 | 3/19/2017 | 3/22/2017 | 3.9847 | 7.4522 | DC6 | Northern Cape |
| 4 | 1/1/2014 | 1/2/2014 | 1/4/2014 | 5.9735 | 8.7423 | DC8 | Northern Cape |
| 4 | 2/9/2016 | 2/10/2016 | 2/12/2016 | 6.8723 | 7.7598 | DC8 | Northern Cape |
| 4 | 12/17/2016 | 12/18/2016 | 12/20/2016 | 8.0404 | 10.1466 | DC8 | Northern Cape |
| 4 | 11/11/2018 | 11/12/2018 | 11/14/2018 | 9.8855 | 11.0855 | DC8 | Northern Cape |
| 4 | 1/30/2016 | 2/1/2016 | 2/2/2016 | 1.551 | 2.5848 | DC9 | Northern Cape |
| 4 | 1/13/2018 | 1/13/2018 | 1/16/2018 | 5.2923 | 7.3923 | DC9 | Northern Cape |
| 4 | 12/24/2018 | 12/24/2018 | 12/27/2018 | 7.9466 | 8.5466 | DC9 | Northern Cape |
| 4 | 11/10/2015 | 11/10/2015 | 11/13/2015 | 4.2855 | 6.4205 | TSH | Gauteng |
| 3 | 3/21/2014 | 3/22/2014 | 3/23/2014 | 3.1555 | 6.3722 | BUF | Eastern Cape |
| 3 | 3/21/2017 | 3/22/2017 | 3/23/2017 | 1.8388 | 2.2222 | BUF | Eastern Cape |
| 3 | 7/6/2017 | 7/6/2017 | 7/8/2017 | 2.7189 | 4.2189 | BUF | Eastern Cape |
| 3 | 9/8/2019 | 9/9/2019 | 9/10/2019 | 2.8763 | 4.0596 | BUF | Eastern Cape |
| 3 | 11/25/2019 | 11/25/2019 | 11/27/2019 | 1.8439 | 2.5105 | BUF | Eastern Cape |
| 3 | 2/15/2014 | 2/16/2014 | 2/17/2014 | 3.9848 | 5.3598 | DC1 | Western Cape |
| 3 | 12/29/2015 | 12/29/2015 | 12/31/2015 | 6.6508 | 9.4216 | DC1 | Western Cape |
| 3 | 1/30/2016 | 1/31/2016 | 2/1/2016 | 5.8856 | 7.2298 | DC1 | Western Cape |
| 3 | 3/20/2017 | 3/20/2017 | 3/22/2017 | 6.4347 | 8.3722 | DC1 | Western Cape |
| 3 | 12/6/2017 | 12/6/2017 | 12/8/2017 | 7.08 | 7.5466 | DC1 | Western Cape |
| 3 | 2/12/2018 | 2/12/2018 | 2/14/2018 | 2.5044 | 3.3598 | DC1 | Western Cape |
| 3 | 1/4/2016 | 1/5/2016 | 1/6/2016 | 5.4145 | 7.8256 | DC22 | KwaZulu-Natal |
| 3 | 3/5/2016 | 3/7/2016 | 3/7/2016 | 4.1 | 6.2888 | DC22 | KwaZulu-Natal |
| 3 | 2/13/2016 | 2/15/2016 | 2/15/2016 | 0.8598 | 1.2848 | DC27 | KwaZulu-Natal |
| 3 | 3/5/2016 | 3/7/2016 | 3/7/2016 | 1.2555 | 2.1555 | DC27 | KwaZulu-Natal |
| 3 | 12/22/2016 | 12/22/2016 | 12/24/2016 | 2.0883 | 3.5716 | DC27 | KwaZulu-Natal |
| 3 | 10/28/2014 | 10/30/2014 | 10/30/2014 | 5.0617 | 6.2284 | DC29 | KwaZulu-Natal |
| 3 | 1/24/2015 | 1/24/2015 | 1/26/2015 | 4.7923 | 7.6923 | DC29 | KwaZulu-Natal |
| 3 | 3/14/2015 | 3/15/2015 | 3/16/2015 | 0.8222 | 1.2222 | DC29 | KwaZulu-Natal |
| 3 | 10/30/2015 | 10/30/2015 | 11/1/2015 | 1.1391 | 1.9284 | DC29 | KwaZulu-Natal |
| 3 | 11/5/2015 | 11/6/2015 | 11/7/2015 | 5.7939 | 10.3605 | DC29 | KwaZulu-Natal |
| 3 | 11/29/2015 | 12/1/2015 | 12/1/2015 | 2.6059 | 4.5966 | DC29 | KwaZulu-Natal |
| 3 | 12/31/2016 | 1/1/2017 | 1/2/2017 | 3.2937 | 4.4923 | DC29 | KwaZulu-Natal |
| 3 | 1/22/2017 | 1/23/2017 | 1/24/2017 | 3.8589 | 6.6923 | DC29 | KwaZulu-Natal |
| 3 | 11/12/2017 | 11/13/2017 | 11/14/2017 | 2.6605 | 5.0605 | DC29 | KwaZulu-Natal |
| 3 | 11/19/2017 | 11/20/2017 | 11/21/2017 | 4.9272 | 6.1605 | DC29 | KwaZulu-Natal |
| 3 | 1/17/2018 | 1/19/2018 | 1/19/2018 | 2.2923 | 3.6923 | DC29 | KwaZulu-Natal |
| 3 | 2/27/2018 | 2/27/2018 | 3/1/2018 | 2.1306 | 3.9848 | DC29 | KwaZulu-Natal |
| 3 | 10/9/2018 | 10/11/2018 | 10/11/2018 | 3.595 | 5.3284 | DC29 | KwaZulu-Natal |
| 3 | 10/16/2019 | 10/17/2019 | 10/18/2019 | 3.495 | 7.7284 | DC29 | KwaZulu-Natal |
| 3 | 12/6/2014 | 12/6/2014 | 12/8/2014 | 4.868 | 5.368 | DC2 | Western Cape |
| 3 | 2/9/2017 | 2/10/2017 | 2/11/2017 | 4.9491 | 6.4848 | DC2 | Western Cape |
| 3 | 12/6/2017 | 12/8/2017 | 12/8/2017 | 7.5903 | 9.9133 | DC2 | Western Cape |
| 3 | 1/16/2018 | 1/16/2018 | 1/18/2018 | 5.0446 | 6.9208 | DC2 | Western Cape |
| 3 | 1/26/2019 | 1/28/2019 | 1/28/2019 | 4.8208 | 6.6923 | DC2 | Western Cape |
| 3 | 11/11/2015 | 11/11/2015 | 11/13/2015 | 6.4605 | 7.8605 | DC33 | Limpopo |
| 3 | 10/29/2016 | 10/29/2016 | 10/31/2016 | 6.5284 | 6.9284 | DC33 | Limpopo |
| 3 | 12/22/2016 | 12/22/2016 | 12/24/2016 | 3.33 | 4.8466 | DC33 | Limpopo |
| 3 | 11/26/2018 | 11/28/2018 | 11/28/2018 | 6.6439 | 11.0105 | DC33 | Limpopo |
| 3 | 1/11/2019 | 1/12/2019 | 1/13/2019 | 1.8756 | 3.7923 | DC33 | Limpopo |
| 3 | 12/25/2019 | 12/25/2019 | 12/27/2019 | 6.2855 | 6.7966 | DC33 | Limpopo |
| 3 | 1/23/2014 | 1/25/2014 | 1/25/2014 | 2.1523 | 2.8923 | DC36 | Limpopo |
| 3 | 10/24/2015 | 10/26/2015 | 10/26/2015 | 5.0417 | 6.0884 | DC36 | Limpopo |
| 3 | 11/25/2015 | 11/27/2015 | 11/27/2015 | 5.4339 | 6.6405 | DC36 | Limpopo |
| 3 | 2/2/2016 | 2/2/2016 | 2/4/2016 | 4.0581 | 4.5248 | DC36 | Limpopo |
| 3 | 3/5/2016 | 3/7/2016 | 3/7/2016 | 4.8088 | 5.2622 | DC36 | Limpopo |
| 3 | 11/4/2016 | 11/4/2016 | 11/6/2016 | 4.0739 | 4.8405 | DC36 | Limpopo |
| 3 | 12/8/2016 | 12/9/2016 | 12/10/2016 | 2.6166 | 3.2366 | DC36 | Limpopo |
| 3 | 11/20/2017 | 11/21/2017 | 11/22/2017 | 3.6005 | 6.5805 | DC36 | Limpopo |
| 3 | 1/6/2018 | 1/6/2018 | 1/8/2018 | 7.3123 | 8.9923 | DC36 | Limpopo |
| 3 | 11/26/2018 | 11/28/2018 | 11/28/2018 | 2.8939 | 4.7855 | DC36 | Limpopo |
| 3 | 1/31/2017 | 2/2/2017 | 2/2/2017 | 1.0206 | 1.9848 | DC37 | North West |
| 3 | 2/11/2017 | 2/12/2017 | 2/13/2017 | 1.3626 | 2.1848 | DC37 | North West |
| 3 | 4/6/2018 | 4/6/2018 | 4/8/2018 | 4.1914 | 4.8247 | DC37 | North West |
| 3 | 5/21/2018 | 5/23/2018 | 5/23/2018 | 5.0981 | 7.8092 | DC37 | North West |
| 3 | 12/24/2019 | 12/25/2019 | 12/26/2019 | 2.6966 | 4.5966 | DC37 | North West |
| 3 | 1/16/2019 | 1/17/2019 | 1/18/2019 | 8.1811 | 8.6923 | DC39 | North West |
| 3 | 1/17/2014 | 1/17/2014 | 1/19/2014 | 4.97 | 7.4923 | DC45 | Northern Cape |
| 3 | 1/30/2016 | 1/31/2016 | 2/1/2016 | 3.8787 | 4.7923 | DC45 | Northern Cape |
| 3 | 1/14/2018 | 1/16/2018 | 1/16/2018 | 5.5145 | 7.8923 | DC45 | Northern Cape |
| 3 | 11/28/2019 | 11/28/2019 | 11/30/2019 | 5.3827 | 6.9939 | DC45 | Northern Cape |
| 3 | 12/24/2014 | 12/24/2014 | 12/26/2014 | 3.6966 | 5.1966 | DC47 | Limpopo |
| 3 | 1/6/2016 | 1/7/2016 | 1/8/2016 | 7.0256 | 9.8923 | DC47 | Limpopo |
| 3 | 1/11/2019 | 1/12/2019 | 1/13/2019 | 3.7256 | 4.4923 | DC47 | Limpopo |
| 3 | 12/25/2019 | 12/25/2019 | 12/27/2019 | 4.8633 | 5.0966 | DC47 | Limpopo |
| 3 | 11/10/2015 | 11/10/2015 | 11/12/2015 | 5.2939 | 9.4605 | DC48 | Gauteng |
| 3 | 1/1/2014 | NA | 1/3/2014 | NA | NA | DC6 | Northern Cape |
| 3 | 1/10/2016 | 1/10/2016 | 1/12/2016 | 3.2023 | 4.9423 | DC6 | Northern Cape |
| 3 | 12/30/2016 | 1/1/2017 | 1/1/2017 | 5.5174 | 6.3623 | DC6 | Northern Cape |
| 3 | 12/6/2017 | 12/6/2017 | 12/8/2017 | 3.86 | 4.5366 | DC6 | Northern Cape |
| 3 | 10/26/2018 | 10/26/2018 | 10/28/2018 | 7.065 | 8.2084 | DC6 | Northern Cape |
| 3 | 2/5/2019 | 2/6/2019 | 2/7/2019 | 5.0714 | 5.8248 | DC6 | Northern Cape |
| 3 | 3/9/2015 | 3/9/2015 | 3/11/2015 | 5.6666 | 7.1555 | DC8 | Northern Cape |
| 3 | 12/18/2015 | 12/18/2015 | 12/20/2015 | 8.2216 | 11.2716 | DC8 | Northern Cape |
| 3 | 1/30/2016 | 1/30/2016 | 2/1/2016 | 6.8398 | 8.3923 | DC8 | Northern Cape |
| 3 | 11/28/2016 | 11/29/2016 | 11/30/2016 | 9.9522 | 11.1855 | DC8 | Northern Cape |
| 3 | 12/30/2016 | 12/30/2016 | 1/1/2017 | 5.4035 | 7.7966 | DC8 | Northern Cape |
| 3 | 2/8/2017 | 2/9/2017 | 2/10/2017 | 3.7181 | 4.6348 | DC8 | Northern Cape |
| 3 | 1/4/2018 | 1/4/2018 | 1/6/2018 | 7.3589 | 7.4673 | DC8 | Northern Cape |
| 3 | 3/4/2018 | 3/5/2018 | 3/6/2018 | 4.4222 | 6.7888 | DC8 | Northern Cape |
| 3 | 12/16/2018 | 12/16/2018 | 12/18/2018 | 5.33 | 10.0966 | DC8 | Northern Cape |
| 3 | 3/15/2019 | 3/15/2019 | 3/17/2019 | 4.6972 | 5.6722 | DC8 | Northern Cape |
| 3 | 1/23/2015 | 1/23/2015 | 1/25/2015 | 6.5423 | 7.9423 | DC9 | Northern Cape |
| 3 | 12/18/2016 | 12/20/2016 | 12/20/2016 | 4.8466 | 6.5466 | DC9 | Northern Cape |
| 3 | 1/16/2019 | 1/17/2019 | 1/18/2019 | 9.4423 | 10.1423 | DC9 | Northern Cape |
| 3 | 1/16/2019 | 1/18/2019 | 1/18/2019 | 5.51 | 7.6656 | MAN | Free State |
| 2 | 1/22/2014 | 1/23/2014 | 1/23/2014 | 2.0423 | 3.3423 | BUF | Eastern Cape |
| 2 | 1/27/2014 | 1/27/2014 | 1/28/2014 | 1.6673 | 2.4423 | BUF | Eastern Cape |
| 2 | 5/3/2014 | 5/4/2014 | 5/4/2014 | 7.1259 | 10.3759 | BUF | Eastern Cape |
| 2 | 5/23/2014 | 5/24/2014 | 5/24/2014 | 1.6259 | 2.3759 | BUF | Eastern Cape |
| 2 | 8/12/2014 | 8/13/2014 | 8/13/2014 | 3.6409 | 6.3909 | BUF | Eastern Cape |
| 2 | 8/18/2014 | 8/19/2014 | 8/19/2014 | 2.7409 | 3.6409 | BUF | Eastern Cape |
| 2 | 1/15/2015 | 1/16/2015 | 1/16/2015 | 1.6173 | 1.6923 | BUF | Eastern Cape |
| 2 | 1/23/2015 | 1/24/2015 | 1/24/2015 | 1.7923 | 2.4923 | BUF | Eastern Cape |
| 2 | 3/3/2015 | 3/4/2015 | 3/4/2015 | 3.1472 | 3.4722 | BUF | Eastern Cape |
| 2 | 5/20/2015 | 5/20/2015 | 5/21/2015 | 0.9259 | 1.4259 | BUF | Eastern Cape |
| 2 | 11/5/2015 | 11/5/2015 | 11/6/2015 | 4.7605 | 7.1105 | BUF | Eastern Cape |
| 2 | 11/30/2015 | 11/30/2015 | 12/1/2015 | 0.7786 | 1.4605 | BUF | Eastern Cape |
| 2 | 12/27/2015 | 12/27/2015 | 12/28/2015 | 2.4966 | 2.5966 | BUF | Eastern Cape |
| 2 | 1/29/2016 | 1/29/2016 | 1/30/2016 | 1.6923 | 1.9423 | BUF | Eastern Cape |
| 2 | 2/23/2016 | 2/23/2016 | 2/24/2016 | 1.2098 | 2.0348 | BUF | Eastern Cape |
| 2 | 2/28/2016 | 2/29/2016 | 2/29/2016 | 1.5598 | 2.5848 | BUF | Eastern Cape |
| 2 | 5/31/2016 | 6/1/2016 | 6/1/2016 | 1.0802 | 1.7846 | BUF | Eastern Cape |
| 2 | 1/27/2017 | 1/28/2017 | 1/28/2017 | 0.6673 | 0.8423 | BUF | Eastern Cape |
| 2 | 3/30/2017 | 3/30/2017 | 3/31/2017 | 2.8472 | 5.5722 | BUF | Eastern Cape |
| 2 | 5/23/2017 | 5/23/2017 | 5/24/2017 | 3.7759 | 7.2759 | BUF | Eastern Cape |
| 2 | 6/6/2017 | 6/6/2017 | 6/7/2017 | 3.9096 | 4.7846 | BUF | Eastern Cape |
| 2 | 9/4/2017 | 9/5/2017 | 9/5/2017 | 6.5346 | 7.1096 | BUF | Eastern Cape |
| 2 | 10/22/2017 | 10/23/2017 | 10/23/2017 | 5.3784 | 8.7284 | BUF | Eastern Cape |
| 2 | 10/31/2017 | 10/31/2017 | 11/1/2017 | 2.5944 | 3.7284 | BUF | Eastern Cape |
| 2 | 1/11/2018 | 1/11/2018 | 1/12/2018 | 1.5673 | 1.6423 | BUF | Eastern Cape |
| 2 | 2/8/2018 | 2/9/2018 | 2/9/2018 | 1.6348 | 2.8348 | BUF | Eastern Cape |
| 2 | 3/27/2018 | 3/28/2018 | 3/28/2018 | 0.4722 | 0.7722 | BUF | Eastern Cape |
| 2 | 6/13/2018 | 6/13/2018 | 6/14/2018 | 1.1846 | 1.8346 | BUF | Eastern Cape |
| 2 | 7/23/2018 | 7/23/2018 | 7/24/2018 | 3.9189 | 4.2189 | BUF | Eastern Cape |
| 2 | 9/30/2018 | 9/30/2018 | 10/1/2018 | 4.019 | 4.5096 | BUF | Eastern Cape |
| 2 | 11/30/2018 | 11/30/2018 | 12/1/2018 | 0.6786 | 1.3105 | BUF | Eastern Cape |
| 2 | 4/29/2019 | 4/29/2019 | 4/30/2019 | 3.758 | 4.708 | BUF | Eastern Cape |
| 2 | 5/19/2019 | 5/19/2019 | 5/20/2019 | 7.8259 | 8.0759 | BUF | Eastern Cape |
| 2 | 10/16/2019 | 10/17/2019 | 10/17/2019 | 5.4034 | 7.4284 | BUF | Eastern Cape |
| 2 | 11/5/2019 | 11/5/2019 | 11/6/2019 | 0.9605 | 1.0105 | BUF | Eastern Cape |
| 2 | 3/23/2014 | 3/23/2014 | 3/24/2014 | 2.5722 | 2.5722 | DC12 | Eastern Cape |
| 2 | 2/24/2016 | 2/24/2016 | 2/25/2016 | 3.4598 | 6.4848 | DC12 | Eastern Cape |
| 2 | 12/17/2018 | 12/18/2018 | 12/18/2018 | 5.9466 | 7.4966 | DC12 | Eastern Cape |
| 2 | 2/25/2014 | 2/26/2014 | 2/26/2014 | 3.8598 | 4.8223 | DC1 | Western Cape |
| 2 | 3/2/2015 | 3/3/2015 | 3/3/2015 | 8.9534 | 10.4097 | DC1 | Western Cape |
| 2 | 1/4/2016 | 1/4/2016 | 1/5/2016 | 4.6735 | 5.0423 | DC1 | Western Cape |
| 2 | 2/6/2019 | 2/6/2019 | 2/7/2019 | 9.0759 | 9.5098 | DC1 | Western Cape |
| 2 | 1/28/2014 | 1/28/2014 | 1/29/2014 | 0.9923 | 1.4256 | DC22 | KwaZulu-Natal |
| 2 | 2/11/2014 | 2/12/2014 | 2/12/2014 | 3.7514 | 5.3848 | DC22 | KwaZulu-Natal |
| 2 | 12/1/2015 | 12/1/2015 | 12/2/2015 | 5.3966 | 9.7966 | DC22 | KwaZulu-Natal |
| 2 | 12/18/2015 | 12/18/2015 | 12/19/2015 | 5.68 | 5.8966 | DC22 | KwaZulu-Natal |
| 2 | 12/8/2016 | 12/8/2016 | 12/9/2016 | 4.3716 | 4.8466 | DC22 | KwaZulu-Natal |
| 2 | 1/1/2017 | 1/2/2017 | 1/2/2017 | 4.1423 | 5.7423 | DC22 | KwaZulu-Natal |
| 2 | 1/23/2017 | 1/23/2017 | 1/24/2017 | 3.0923 | 4.6423 | DC22 | KwaZulu-Natal |
| 2 | 2/1/2017 | 2/1/2017 | 2/2/2017 | 4.5848 | 4.7348 | DC22 | KwaZulu-Natal |
| 2 | 4/4/2017 | 4/4/2017 | 4/5/2017 | 6.558 | 7.408 | DC22 | KwaZulu-Natal |
| 2 | 12/6/2018 | 12/6/2018 | 12/7/2018 | 6.6966 | 7.1633 | DC22 | KwaZulu-Natal |
| 2 | 12/17/2018 | 12/17/2018 | 12/18/2018 | 6.2466 | 6.4966 | DC22 | KwaZulu-Natal |
| 2 | 11/29/2019 | 11/30/2019 | 11/30/2019 | 7.0939 | 7.1605 | DC22 | KwaZulu-Natal |
| 2 | 12/24/2019 | 12/24/2019 | 12/25/2019 | 3.5633 | 6.0633 | DC22 | KwaZulu-Natal |
| 2 | 12/20/2014 | 12/20/2014 | 12/21/2014 | 0.6091 | 0.6716 | DC27 | KwaZulu-Natal |
| 2 | 1/4/2015 | 1/5/2015 | 1/5/2015 | 1.3173 | 1.8423 | DC27 | KwaZulu-Natal |
| 2 | 10/7/2015 | 10/7/2015 | 10/8/2015 | 3.5534 | 4.7784 | DC27 | KwaZulu-Natal |
| 2 | 11/25/2015 | 11/26/2015 | 11/26/2015 | 0.7439 | 1.2605 | DC27 | KwaZulu-Natal |
| 2 | 12/18/2015 | 12/19/2015 | 12/19/2015 | 2.4133 | 2.43 | DC27 | KwaZulu-Natal |
| 2 | 1/23/2016 | 1/24/2016 | 1/24/2016 | 0.6423 | 1.1673 | DC27 | KwaZulu-Natal |
| 2 | 2/2/2016 | 2/2/2016 | 2/3/2016 | 1.5014 | 2.6181 | DC27 | KwaZulu-Natal |
| 2 | 2/19/2016 | 2/20/2016 | 2/20/2016 | 3.7598 | 6.0098 | DC27 | KwaZulu-Natal |
| 2 | 12/8/2016 | 12/9/2016 | 12/9/2016 | 1.8841 | 2.8966 | DC27 | KwaZulu-Natal |
| 2 | 2/11/2017 | 2/11/2017 | 2/12/2017 | 0.8848 | 0.9098 | DC27 | KwaZulu-Natal |
| 2 | 11/26/2018 | 11/27/2018 | 11/27/2018 | 1.3355 | 2.4605 | DC27 | KwaZulu-Natal |
| 2 | 12/17/2018 | 12/18/2018 | 12/18/2018 | 1.4466 | 2.7966 | DC27 | KwaZulu-Natal |
| 2 | 11/6/2019 | 11/7/2019 | 11/7/2019 | 2.1605 | 3.3355 | DC27 | KwaZulu-Natal |
| 2 | 1/5/2014 | 1/5/2014 | 1/6/2014 | 0.9923 | 1.8923 | DC29 | KwaZulu-Natal |
| 2 | 1/23/2014 | 1/24/2014 | 1/24/2014 | 3.6423 | 3.8923 | DC29 | KwaZulu-Natal |
| 2 | 1/28/2014 | 1/28/2014 | 1/29/2014 | 0.8423 | 1.1923 | DC29 | KwaZulu-Natal |
| 2 | 3/14/2014 | 3/15/2014 | 3/15/2014 | 0.6722 | 0.9222 | DC29 | KwaZulu-Natal |
| 2 | 3/22/2014 | 3/23/2014 | 3/23/2014 | 1.6222 | 2.2222 | DC29 | KwaZulu-Natal |
| 2 | 3/31/2014 | 4/1/2014 | 4/1/2014 | 3.0401 | 3.258 | DC29 | KwaZulu-Natal |
| 2 | 4/11/2014 | 4/12/2014 | 4/12/2014 | 4.408 | 5.458 | DC29 | KwaZulu-Natal |
| 2 | 9/23/2014 | 9/23/2014 | 9/24/2014 | 2.2096 | 4.2096 | DC29 | KwaZulu-Natal |
| 2 | 12/3/2014 | 12/3/2014 | 12/4/2014 | 2.2966 | 4.2966 | DC29 | KwaZulu-Natal |
| 2 | 1/12/2015 | 1/12/2015 | 1/13/2015 | 2.2923 | 4.2923 | DC29 | KwaZulu-Natal |
| 2 | 3/24/2015 | 3/24/2015 | 3/25/2015 | 0.5222 | 1.0222 | DC29 | KwaZulu-Natal |
| 2 | 4/13/2015 | 4/14/2015 | 4/14/2015 | 3.458 | 4.058 | DC29 | KwaZulu-Natal |
| 2 | 5/18/2015 | 5/19/2015 | 5/19/2015 | 4.2759 | 4.8759 | DC29 | KwaZulu-Natal |
| 2 | 7/11/2015 | 7/12/2015 | 7/12/2015 | 2.7689 | 4.9189 | DC29 | KwaZulu-Natal |
| 2 | 9/24/2015 | 9/25/2015 | 9/25/2015 | 1.4596 | 2.6096 | DC29 | KwaZulu-Natal |
| 2 | 10/12/2015 | 10/13/2015 | 10/13/2015 | 8.6284 | 10.6284 | DC29 | KwaZulu-Natal |
| 2 | 10/20/2015 | 10/21/2015 | 10/21/2015 | 4.4784 | 5.3284 | DC29 | KwaZulu-Natal |
| 2 | 1/4/2016 | 1/5/2016 | 1/5/2016 | 4.3423 | 6.6923 | DC29 | KwaZulu-Natal |
| 2 | 2/13/2016 | 2/14/2016 | 2/14/2016 | 3.7348 | 5.8848 | DC29 | KwaZulu-Natal |
| 2 | 2/23/2016 | 2/24/2016 | 2/24/2016 | 2.4848 | 3.1848 | DC29 | KwaZulu-Natal |
| 2 | 3/26/2016 | 3/27/2016 | 3/27/2016 | 3.4222 | 4.7222 | DC29 | KwaZulu-Natal |
| 2 | 9/15/2016 | 9/15/2016 | 9/16/2016 | 2.7596 | 3.3096 | DC29 | KwaZulu-Natal |
| 2 | 9/26/2016 | 9/27/2016 | 9/27/2016 | 2.9596 | 3.8096 | DC29 | KwaZulu-Natal |
| 2 | 12/18/2016 | 12/19/2016 | 12/19/2016 | 2.4966 | 4.6966 | DC29 | KwaZulu-Natal |
| 2 | 12/23/2016 | 12/23/2016 | 12/24/2016 | 6.9966 | 7.5966 | DC29 | KwaZulu-Natal |
| 2 | 2/1/2017 | 2/2/2017 | 2/2/2017 | 2.4348 | 4.5848 | DC29 | KwaZulu-Natal |
| 2 | 2/28/2017 | 3/1/2017 | 3/1/2017 | 0.4035 | 0.5222 | DC29 | KwaZulu-Natal |
| 2 | 3/30/2017 | 3/31/2017 | 3/31/2017 | 3.3222 | 5.5222 | DC29 | KwaZulu-Natal |
| 2 | 5/5/2017 | 5/5/2017 | 5/6/2017 | 3.5759 | 3.7759 | DC29 | KwaZulu-Natal |
| 2 | 9/21/2017 | 9/22/2017 | 9/22/2017 | 3.5096 | 4.6096 | DC29 | KwaZulu-Natal |
| 2 | 1/12/2018 | 1/12/2018 | 1/13/2018 | 2.6923 | 2.6923 | DC29 | KwaZulu-Natal |
| 2 | 3/10/2018 | 3/10/2018 | 3/11/2018 | 3.3222 | 3.8222 | DC29 | KwaZulu-Natal |
| 2 | 10/1/2018 | 10/1/2018 | 10/2/2018 | 8.3284 | 11.4284 | DC29 | KwaZulu-Natal |
| 2 | 10/29/2018 | 10/30/2018 | 10/30/2018 | 4.3284 | 7.9284 | DC29 | KwaZulu-Natal |
| 2 | 11/24/2018 | 11/25/2018 | 11/25/2018 | 4.8605 | 6.2605 | DC29 | KwaZulu-Natal |
| 2 | 11/30/2018 | 12/1/2018 | 12/1/2018 | 2.2786 | 2.8966 | DC29 | KwaZulu-Natal |
| 2 | 12/6/2018 | 12/7/2018 | 12/7/2018 | 4.8966 | 7.0966 | DC29 | KwaZulu-Natal |
| 2 | 12/24/2018 | 12/25/2018 | 12/25/2018 | 0.8466 | 1.4966 | DC29 | KwaZulu-Natal |
| 2 | 4/13/2019 | 4/13/2019 | 4/14/2019 | 2.308 | 2.458 | DC29 | KwaZulu-Natal |
| 2 | 5/19/2019 | 5/20/2019 | 5/20/2019 | 5.8259 | 6.9759 | DC29 | KwaZulu-Natal |
| 2 | 12/17/2019 | 12/18/2019 | 12/18/2019 | 4.6466 | 7.7966 | DC29 | KwaZulu-Natal |
| 2 | 12/23/2019 | 12/24/2019 | 12/24/2019 | 3.2466 | 5.0966 | DC29 | KwaZulu-Natal |
| 2 | 1/21/2014 | 1/22/2014 | 1/22/2014 | 6.5423 | 8.0923 | DC2 | Western Cape |
| 2 | 2/26/2014 | 2/27/2014 | 2/27/2014 | 5.9419 | 7.3562 | DC2 | Western Cape |
| 2 | 3/5/2014 | 3/5/2014 | 3/6/2014 | 3.9436 | 4.5222 | DC2 | Western Cape |
| 2 | 1/6/2015 | 1/7/2015 | 1/7/2015 | 7.8637 | 10.178 | DC2 | Western Cape |
| 2 | 1/28/2015 | 1/29/2015 | 1/29/2015 | 5.4851 | 6.5208 | DC2 | Western Cape |
| 2 | 3/2/2015 | 3/3/2015 | 3/3/2015 | 8.021 | 12.1508 | DC2 | Western Cape |
| 2 | 4/9/2015 | 4/9/2015 | 4/10/2015 | 6.2437 | 7.6295 | DC2 | Western Cape |
| 2 | 10/27/2015 | 10/27/2015 | 10/28/2015 | 9.7141 | 11.1569 | DC2 | Western Cape |
| 2 | 12/13/2016 | 12/14/2016 | 12/14/2016 | 5.3966 | 7.7395 | DC2 | Western Cape |
| 2 | 1/17/2017 | 1/18/2017 | 1/18/2017 | 7.9708 | 9.5065 | DC2 | Western Cape |
| 2 | 1/22/2017 | 1/22/2017 | 1/23/2017 | 6.8065 | 8.8923 | DC2 | Western Cape |
| 2 | 2/27/2017 | 2/27/2017 | 2/28/2017 | 9.0562 | 9.2134 | DC2 | Western Cape |
| 2 | 10/30/2017 | 10/30/2017 | 10/31/2017 | 8.7284 | 10.2998 | DC2 | Western Cape |
| 2 | 12/29/2017 | 12/30/2017 | 12/30/2017 | 5.5395 | 5.7395 | DC2 | Western Cape |
| 2 | 1/3/2018 | 1/3/2018 | 1/4/2018 | 9.628 | 10.4494 | DC2 | Western Cape |
| 2 | 1/10/2018 | 1/11/2018 | 1/11/2018 | 9.1923 | 10.5208 | DC2 | Western Cape |
| 2 | 1/28/2018 | 1/29/2018 | 1/29/2018 | 6.1065 | 6.678 | DC2 | Western Cape |
| 2 | 2/7/2018 | 2/7/2018 | 2/8/2018 | 6.7419 | 6.8705 | DC2 | Western Cape |
| 2 | 2/21/2018 | 2/22/2018 | 2/22/2018 | 3.8134 | 6.0848 | DC2 | Western Cape |
| 2 | 12/16/2018 | 12/16/2018 | 12/17/2018 | 6.0109 | 6.8966 | DC2 | Western Cape |
| 2 | 3/15/2019 | 3/15/2019 | 3/16/2019 | 1.8579 | 2.3079 | DC2 | Western Cape |
| 2 | 11/1/2015 | 11/2/2015 | 11/2/2015 | 7.3605 | 9.5605 | DC33 | Limpopo |
| 2 | 1/6/2016 | 1/7/2016 | 1/7/2016 | 7.7673 | 8.6423 | DC33 | Limpopo |
| 2 | 2/19/2016 | 2/20/2016 | 2/20/2016 | 4.1848 | 5.2848 | DC33 | Limpopo |
| 2 | 10/23/2016 | 10/23/2016 | 10/24/2016 | 5.5784 | 7.4284 | DC33 | Limpopo |
| 2 | 11/5/2016 | 11/5/2016 | 11/6/2016 | 4.3355 | 4.8105 | DC33 | Limpopo |
| 2 | 11/30/2016 | 12/1/2016 | 12/1/2016 | 4.2286 | 6.2466 | DC33 | Limpopo |
| 2 | 12/18/2018 | 12/18/2018 | 12/19/2018 | 4.6216 | 4.9466 | DC33 | Limpopo |
| 2 | 12/26/2018 | 12/27/2018 | 12/27/2018 | 6.5966 | 7.2966 | DC33 | Limpopo |
| 2 | 10/20/2019 | 10/21/2019 | 10/21/2019 | 9.6117 | 10.795 | DC33 | Limpopo |
| 2 | 10/30/2014 | 10/30/2014 | 10/31/2014 | 6.4684 | 7.8884 | DC36 | Limpopo |
| 2 | 12/25/2014 | 12/26/2014 | 12/26/2014 | 1.3366 | 1.4166 | DC36 | Limpopo |
| 2 | 1/26/2015 | 1/27/2015 | 1/27/2015 | 1.8923 | 2.5523 | DC36 | Limpopo |
| 2 | 3/16/2015 | 3/17/2015 | 3/17/2015 | 4.6622 | 5.7022 | DC36 | Limpopo |
| 2 | 10/31/2015 | 10/31/2015 | 11/1/2015 | 7.5444 | 8.6884 | DC36 | Limpopo |
| 2 | 1/27/2016 | 1/28/2016 | 1/28/2016 | 2.1223 | 2.1523 | DC36 | Limpopo |
| 2 | 10/16/2016 | 10/16/2016 | 10/17/2016 | 3.7984 | 4.2284 | DC36 | Limpopo |
| 2 | 12/22/2016 | 12/22/2016 | 12/23/2016 | 3.4091 | 4.1616 | DC36 | Limpopo |
| 2 | 1/2/2017 | 1/3/2017 | 1/3/2017 | 2.0523 | 2.7323 | DC36 | Limpopo |
| 2 | 10/25/2017 | 10/25/2017 | 10/26/2017 | 3.8784 | 7.7284 | DC36 | Limpopo |
| 2 | 12/3/2017 | 12/3/2017 | 12/4/2017 | 2.7766 | 4.8566 | DC36 | Limpopo |
| 2 | 12/18/2017 | 12/18/2017 | 12/19/2017 | 3.5166 | 5.2766 | DC36 | Limpopo |
| 2 | 12/31/2017 | 1/1/2018 | 1/1/2018 | 5.9244 | 6.8123 | DC36 | Limpopo |
| 2 | 1/14/2018 | 1/15/2018 | 1/15/2018 | 4.0623 | 4.8123 | DC36 | Limpopo |
| 2 | 1/21/2018 | 1/21/2018 | 1/22/2018 | 2.3123 | 2.5923 | DC36 | Limpopo |
| 2 | 2/2/2018 | 2/2/2018 | 2/3/2018 | 1.4948 | 2.0048 | DC36 | Limpopo |
| 2 | 9/18/2018 | 9/19/2018 | 9/19/2018 | 9.6696 | 9.8096 | DC36 | Limpopo |
| 2 | 10/30/2018 | 10/30/2018 | 10/31/2018 | 6.1784 | 8.4534 | DC36 | Limpopo |
| 2 | 11/17/2018 | 11/17/2018 | 11/18/2018 | 4.823 | 7.5105 | DC36 | Limpopo |
| 2 | 3/25/2019 | 3/25/2019 | 3/26/2019 | 4.4972 | 4.8472 | DC36 | Limpopo |
| 2 | 10/8/2019 | 10/8/2019 | 10/9/2019 | 8.5084 | 9.8884 | DC36 | Limpopo |
| 2 | 11/7/2019 | 11/7/2019 | 11/8/2019 | 7.3705 | 8.3805 | DC36 | Limpopo |
| 2 | 12/2/2019 | 12/2/2019 | 12/3/2019 | 5.5066 | 5.7566 | DC36 | Limpopo |
| 2 | 8/20/2014 | 8/20/2014 | 8/21/2014 | 6.6159 | 7.8909 | DC37 | North West |
| 2 | 8/15/2016 | 8/15/2016 | 8/16/2016 | 5.9076 | 10.5076 | DC37 | North West |
| 2 | 8/20/2016 | 8/21/2016 | 8/21/2016 | 7.7409 | 9.8743 | DC37 | North West |
| 2 | 3/1/2017 | 3/1/2017 | 3/2/2017 | 1.6388 | 3.1555 | DC37 | North West |
| 2 | 3/20/2018 | 3/20/2018 | 3/21/2018 | 2.9972 | 4.7222 | DC37 | North West |
| 2 | 4/14/2018 | 4/14/2018 | 4/15/2018 | 1.9914 | 2.358 | DC37 | North West |
| 2 | 2/6/2019 | 2/7/2019 | 2/7/2019 | 0.5098 | 0.9848 | DC37 | North West |
| 2 | 5/11/2019 | 5/11/2019 | 5/12/2019 | 4.6759 | 4.7092 | DC37 | North West |
| 2 | 9/4/2019 | 9/5/2019 | 9/5/2019 | 9.3263 | 9.8429 | DC37 | North West |
| 2 | 12/30/2019 | 12/30/2019 | 12/31/2019 | 2.5466 | 3.3966 | DC37 | North West |
| 2 | 11/10/2015 | 11/10/2015 | 11/11/2015 | 6.8355 | 7.7105 | DC38 | North West |
| 2 | 12/6/2015 | 12/7/2015 | 12/7/2015 | 7.6716 | 8.1716 | DC38 | North West |
| 2 | 2/10/2015 | 2/11/2015 | 2/11/2015 | 7.3348 | 8.2348 | DC39 | North West |
| 2 | 12/6/2015 | 12/6/2015 | 12/7/2015 | 11.1091 | 12.3966 | DC39 | North West |
| 2 | 12/24/2015 | 12/24/2015 | 12/25/2015 | 8.6341 | 11.3216 | DC39 | North West |
| 2 | 11/30/2016 | 12/1/2016 | 12/1/2016 | 8.4161 | 9.2966 | DC39 | North West |
| 2 | 1/15/2018 | 1/16/2018 | 1/16/2018 | 4.1673 | 4.5423 | DC39 | North West |
| 2 | 12/24/2018 | 12/24/2018 | 12/25/2018 | 9.7133 | 10.63 | DC39 | North West |
| 2 | 11/28/2019 | 11/29/2019 | 11/29/2019 | 6.048 | 6.2105 | DC39 | North West |
| 2 | 11/11/2015 | 11/11/2015 | 11/12/2015 | 3.6605 | 7.0605 | DC42 | Gauteng |
| 2 | 12/19/2015 | 12/19/2015 | 12/20/2015 | 1.6966 | 2.4966 | DC42 | Gauteng |
| 2 | 10/26/2019 | 10/27/2019 | 10/27/2019 | 3.5784 | 4.2284 | DC42 | Gauteng |
| 2 | 10/25/2015 | 10/26/2015 | 10/26/2015 | 0.8617 | 1.695 | DC45 | Northern Cape |
| 2 | 12/26/2015 | 12/27/2015 | 12/27/2015 | 8.0633 | 8.9966 | DC45 | Northern Cape |
| 2 | 2/12/2016 | 2/12/2016 | 2/13/2016 | 6.6848 | 7.2848 | DC45 | Northern Cape |
| 2 | 10/28/2016 | 10/28/2016 | 10/29/2016 | 8.9117 | 12.395 | DC45 | Northern Cape |
| 2 | 1/6/2018 | 1/6/2018 | 1/7/2018 | 7.3589 | 7.9256 | DC45 | Northern Cape |
| 2 | 11/17/2018 | 11/17/2018 | 11/18/2018 | 8.0272 | 8.0939 | DC45 | Northern Cape |
| 2 | 12/26/2018 | 12/26/2018 | 12/27/2018 | 5.0633 | 8.33 | DC45 | Northern Cape |
| 2 | 1/8/2019 | 1/8/2019 | 1/9/2019 | 5.2589 | 5.3589 | DC45 | Northern Cape |
| 2 | 2/10/2019 | 2/10/2019 | 2/11/2019 | 2.3014 | 3.1181 | DC45 | Northern Cape |
| 2 | 10/8/2015 | 10/9/2015 | 10/9/2015 | 7.7784 | 10.5284 | DC47 | Limpopo |
| 2 | 10/25/2015 | 10/25/2015 | 10/26/2015 | 5.0284 | 6.4284 | DC47 | Limpopo |
| 2 | 12/1/2015 | 12/1/2015 | 12/2/2015 | 5.5966 | 6.7966 | DC47 | Limpopo |
| 2 | 2/3/2016 | 2/3/2016 | 2/4/2016 | 5.7848 | 7.1848 | DC47 | Limpopo |
| 2 | 2/24/2016 | 2/24/2016 | 2/25/2016 | 2.2848 | 3.5848 | DC47 | Limpopo |
| 2 | 3/7/2016 | 3/7/2016 | 3/8/2016 | 2.6722 | 4.9222 | DC47 | Limpopo |
| 2 | 11/4/2016 | 11/5/2016 | 11/5/2016 | 3.8105 | 4.0605 | DC47 | Limpopo |
| 2 | 12/26/2018 | 12/26/2018 | 12/27/2018 | 2.9966 | 3.2966 | DC47 | Limpopo |
| 2 | 12/6/2015 | 12/7/2015 | 12/7/2015 | 6.0466 | 6.3966 | DC48 | Gauteng |
| 2 | 10/29/2016 | 10/30/2016 | 10/30/2016 | 4.5784 | 5.2284 | DC48 | Gauteng |
| 2 | 12/16/2018 | 12/16/2018 | 12/17/2018 | 3.4466 | 3.5966 | DC48 | Gauteng |
| 2 | 10/26/2019 | 10/27/2019 | 10/27/2019 | 4.2284 | 4.6284 | DC48 | Gauteng |
| 2 | 11/27/2019 | 11/27/2019 | 11/28/2019 | 1.5105 | 1.7605 | DC48 | Gauteng |
| 2 | 12/2/2019 | 12/3/2019 | 12/3/2019 | 3.3966 | 4.8966 | DC48 | Gauteng |
| 2 | 1/21/2014 | 1/21/2014 | 1/22/2014 | 2.77 | 4.2367 | DC6 | Northern Cape |
| 2 | 2/26/2014 | 2/26/2014 | 2/27/2014 | 2.8014 | 4.6181 | DC6 | Northern Cape |
| 2 | 10/27/2015 | 10/27/2015 | 10/28/2015 | 6.8484 | 8.1684 | DC6 | Northern Cape |
| 2 | 2/9/2016 | 2/10/2016 | 2/10/2016 | 2.9798 | 3.2948 | DC6 | Northern Cape |
| 2 | 3/4/2016 | 3/4/2016 | 3/5/2016 | 4.6122 | 6.4522 | DC6 | Northern Cape |
| 2 | 1/17/2017 | 1/17/2017 | 1/18/2017 | 7.0223 | 8.7623 | DC6 | Northern Cape |
| 2 | 2/12/2018 | 2/12/2018 | 2/13/2018 | 1.6798 | 2.9348 | DC6 | Northern Cape |
| 2 | 11/27/2019 | 11/27/2019 | 11/28/2019 | 8.3555 | 9.6605 | DC6 | Northern Cape |
| 2 | 2/15/2014 | 2/16/2014 | 2/16/2014 | 2.4848 | 3.0098 | DC8 | Northern Cape |
| 2 | 2/11/2015 | 2/11/2015 | 2/12/2015 | 6.5848 | 10.6598 | DC8 | Northern Cape |
| 2 | 3/4/2016 | 3/4/2016 | 3/5/2016 | 3.2847 | 4.2722 | DC8 | Northern Cape |
| 2 | 12/5/2016 | 12/5/2016 | 12/6/2016 | 8.2591 | 8.6966 | DC8 | Northern Cape |
| 2 | 1/18/2017 | 1/18/2017 | 1/19/2017 | 3.8298 | 3.8423 | DC8 | Northern Cape |
| 2 | 2/14/2017 | 2/14/2017 | 2/15/2017 | 2.8723 | 4.2848 | DC8 | Northern Cape |
| 2 | 2/4/2018 | 2/5/2018 | 2/5/2018 | 8.3681 | 9.2181 | DC8 | Northern Cape |
| 2 | 10/28/2018 | 10/28/2018 | 10/29/2018 | 9.2534 | 10.7284 | DC8 | Northern Cape |
| 2 | 1/28/2019 | 1/28/2019 | 1/29/2019 | 6.1298 | 8.5923 | DC8 | Northern Cape |
| 2 | 11/28/2019 | 11/28/2019 | 11/29/2019 | 5.7605 | 8.1105 | DC8 | Northern Cape |
| 2 | 11/30/2015 | 12/1/2015 | 12/1/2015 | 2.8036 | 2.8466 | DC9 | Northern Cape |
| 2 | 12/6/2015 | 12/6/2015 | 12/7/2015 | 8.6966 | 9.1966 | DC9 | Northern Cape |
| 2 | 12/18/2015 | 12/18/2015 | 12/19/2015 | 3.8466 | 4.7466 | DC9 | Northern Cape |
| 2 | 2/6/2016 | 2/7/2016 | 2/7/2016 | 3.6598 | 4.9348 | DC9 | Northern Cape |
| 2 | 2/12/2016 | 2/12/2016 | 2/13/2016 | 5.1098 | 7.4848 | DC9 | Northern Cape |
| 2 | 10/28/2016 | 10/28/2016 | 10/29/2016 | 7.2284 | 9.8784 | DC9 | Northern Cape |
| 2 | 11/27/2016 | 11/28/2016 | 11/28/2016 | 4.7605 | 6.0605 | DC9 | Northern Cape |
| 2 | 11/12/2018 | 11/12/2018 | 11/13/2018 | 6.5855 | 7.0605 | DC9 | Northern Cape |
| 2 | 1/8/2019 | 1/9/2019 | 1/9/2019 | 5.8423 | 6.0923 | DC9 | Northern Cape |
| 2 | 1/22/2019 | 1/23/2019 | 1/23/2019 | 4.4423 | 4.9423 | DC9 | Northern Cape |
| 2 | 11/28/2019 | 11/28/2019 | 11/29/2019 | 6.3105 | 7.7605 | DC9 | Northern Cape |
| 2 | 12/19/2015 | 12/19/2015 | 12/20/2015 | 5.4533 | 5.6033 | MAN | Free State |
| 2 | 10/28/2016 | 10/28/2016 | 10/29/2016 | 4.585 | 6.6017 | MAN | Free State |
| 2 | 12/19/2016 | 12/19/2016 | 12/20/2016 | 4.82 | 7.1366 | MAN | Free State |
| 2 | 1/14/2018 | 1/14/2018 | 1/15/2018 | 3.6156 | 4.0989 | MAN | Free State |
| 2 | 12/1/2019 | 12/1/2019 | 12/2/2019 | 4.0033 | 5.47 | MAN | Free State |
| 2 | 1/6/2016 | 1/6/2016 | 1/7/2016 | 5.7023 | 5.7723 | TSH | Gauteng |
| 2 | 10/30/2016 | 10/30/2016 | 10/31/2016 | 4.3784 | 5.2034 | TSH | Gauteng |
| 2 | 12/4/2018 | 12/5/2018 | 12/5/2018 | 7.1216 | 7.6466 | TSH | Gauteng |
