# Supplementary material for: An analysis of past and future heatwaves based on a heat-associated mortality threshold: towards a heat health warning system
Source: Environ Health. 2022 Nov 19;21:112. doi: 10.1186/s12940-022-00921-4 (PMC9675182; doi:10.1186/s12940-022-00921-4)
Supplement: Supplementary file 4 — Additional file 4. Heatwave characteristics for period 2020 – 2039 using data simulated from RCP 8.5 projections. [file 12940_2022_921_MOESM4_ESM.docx]

Additional File 4: Heatwave characteristics for period 2020 – 2039 using data simulated from RCP 8.5 projections

| Duration (days) | Date_start | Date_peak | Date_end | Intensity_mean | Intensity_max | District | Province |
| --- | --- | --- | --- | --- | --- | --- | --- |
| 121 | 9/15/2015 | 11/9/2015 | 1/13/2016 | 4.3937 | 10.9667 | DC37 | North West |
| 108 | 9/12/2018 | NA | 12/28/2018 | NA | NA | DC37 | North West |
| 86 | 9/9/2019 | NA | 12/3/2019 | NA | NA | DC37 | North West |
| 79 | 1/2/2015 | 2/7/2015 | 3/21/2015 | 2.896 | 9.5 | DC37 | North West |
| 58 | 10/8/2017 | NA | 12/4/2017 | NA | NA | DC37 | North West |
| 55 | 9/7/2014 | NA | 10/31/2014 | NA | NA | DC37 | North West |
| 53 | 8/26/2016 | NA | 10/17/2016 | NA | NA | DC37 | North West |
| 43 | 1/25/2016 | 2/13/2016 | 3/7/2016 | 2.7977 | 6.4 | DC37 | North West |
| 36 | 3/6/2017 | 4/1/2017 | 4/10/2017 | 3.4491 | 9.3 | DC37 | North West |
| 35 | 4/9/2016 | NA | 5/13/2016 | NA | NA | DC37 | North West |
| 34 | 12/23/2017 | 1/1/2018 | 1/25/2018 | 4.202 | 10.8667 | DC37 | North West |
| 29 | 11/13/2014 | 11/21/2014 | 12/11/2014 | 1.9454 | 8.25 | DC37 | North West |
| 29 | 9/6/2017 | 9/14/2017 | 10/4/2017 | 7.1138 | 13.1333 | DC37 | North West |
| 28 | 4/25/2015 | NA | 5/22/2015 | NA | NA | DC37 | North West |
| 28 | 10/22/2016 | 10/25/2016 | 11/18/2016 | 3.1595 | 8.65 | DC37 | North West |
| 27 | 1/5/2019 | 1/22/2019 | 1/31/2019 | 2.2117 | 8.7333 | DC37 | North West |
| 26 | 3/27/2015 | NA | 4/21/2015 | NA | NA | DC37 | North West |
| 23 | 12/17/2015 | NA | 1/8/2016 | NA | NA | DC36 | Limpopo |
| 21 | 4/14/2018 | 4/24/2018 | 5/4/2018 | 3.9667 | 10.5 | DC37 | North West |
| 21 | 3/13/2019 | 3/21/2019 | 4/2/2019 | 4.6968 | 9.0333 | DC37 | North West |
| 20 | 11/22/2016 | 11/29/2016 | 12/11/2016 | 2.3275 | 5.2333 | DC37 | North West |
| 20 | 2/17/2019 | 3/1/2019 | 3/8/2019 | 4.7733 | 11.0333 | DC37 | North West |
| 20 | 4/25/2019 | 5/4/2019 | 5/14/2019 | 5.1533 | 9.0667 | DC37 | North West |
| 19 | 8/15/2015 | 8/19/2015 | 9/2/2015 | 7.4088 | 10.0333 | DC37 | North West |
| 18 | 1/9/2014 | 1/18/2014 | 1/26/2014 | 3.8611 | 8.2667 | DC37 | North West |
| 18 | 1/31/2018 | 2/17/2018 | 2/17/2018 | 1.8306 | 6.1667 | DC37 | North West |
| 18 | 12/29/2015 | NA | 1/15/2016 | NA | NA | DC8 | Northern Cape |
| 17 | 2/28/2018 | 3/15/2018 | 3/16/2018 | 2.8765 | 6.4 | DC37 | North West |
| 16 | 12/28/2015 | NA | 1/12/2016 | NA | NA | DC2 | Western Cape |
| 16 | 3/19/2016 | 3/29/2016 | 4/3/2016 | 4.5687 | 10.9333 | DC37 | North West |
| 15 | 9/30/2015 | NA | 10/14/2015 | NA | NA | DC36 | Limpopo |
| 14 | 1/29/2016 | NA | 2/11/2016 | NA | NA | DC2 | Western Cape |
| 14 | 12/29/2016 | NA | 1/11/2017 | NA | NA | DC2 | Western Cape |
| 14 | 8/13/2018 | NA | 8/26/2018 | NA | NA | DC37 | North West |
| 14 | 8/4/2019 | NA | 8/17/2019 | NA | NA | DC37 | North West |
| 13 | 12/1/2015 | NA | 12/13/2015 | NA | NA | DC36 | Limpopo |
| 13 | 12/19/2019 | NA | 12/31/2019 | NA | NA | DC36 | Limpopo |
| 13 | 4/28/2017 | 5/5/2017 | 5/10/2017 | 3.9641 | 5.8 | DC37 | North West |
| 13 | 4/9/2019 | 4/17/2019 | 4/21/2019 | 2.7744 | 8.9667 | DC37 | North West |
| 12 | 3/30/2014 | NA | 4/10/2014 | NA | NA | DC37 | North West |
| 12 | 1/15/2019 | NA | 1/26/2019 | NA | NA | DC45 | Northern Cape |
| 12 | 12/28/2015 | NA | 1/8/2016 | NA | NA | DC47 | Limpopo |
| 12 | 1/13/2014 | NA | 1/24/2014 | NA | NA | DC8 | Northern Cape |
| 11 | 2/3/2015 | 2/11/2015 | 2/13/2015 | 2.7577 | 4.72 | DC36 | Limpopo |
| 11 | 2/12/2016 | 2/22/2016 | 2/22/2016 | 2.9364 | 4.86 | DC36 | Limpopo |
| 11 | 2/11/2014 | 2/12/2014 | 2/21/2014 | 1.4545 | 4.55 | DC37 | North West |
| 11 | 12/9/2017 | 12/14/2017 | 12/19/2017 | 3.1788 | 7.9 | DC37 | North West |
| 11 | 3/20/2018 | 3/26/2018 | 3/30/2018 | 0.8197 | 5.9 | DC37 | North West |
| 11 | 8/26/2019 | NA | 9/5/2019 | NA | NA | DC37 | North West |
| 11 | 12/31/2015 | 1/6/2016 | 1/10/2016 | 5.9432 | 7.45 | DC39 | North West |
| 11 | 10/4/2015 | NA | 10/14/2015 | NA | NA | DC47 | Limpopo |
| 11 | 12/1/2015 | NA | 12/11/2015 | NA | NA | DC47 | Limpopo |
| 10 | 10/22/2016 | NA | 10/31/2016 | NA | NA | DC33 | Limpopo |
| 10 | 10/22/2016 | 10/30/2016 | 10/31/2016 | 4.148 | 5.98 | DC36 | Limpopo |
| 10 | 1/15/2017 | 1/19/2017 | 1/24/2017 | 2.7033 | 7.6667 | DC37 | North West |
| 10 | 12/11/2019 | 12/16/2019 | 12/20/2019 | 3.28 | 9.6667 | DC37 | North West |
| 10 | 12/18/2015 | NA | 12/27/2015 | NA | NA | DC45 | Northern Cape |
| 10 | 1/6/2015 | NA | 1/15/2015 | NA | NA | DC8 | Northern Cape |
| 10 | 11/27/2016 | NA | 12/6/2016 | NA | NA | DC8 | Northern Cape |
| 10 | 1/11/2018 | 1/16/2018 | 1/20/2018 | 6.3525 | 11.175 | DC8 | Northern Cape |
| 9 | 1/10/2018 | NA | 1/18/2018 | NA | NA | DC2 | Western Cape |
| 9 | 2/14/2019 | NA | 2/22/2019 | NA | NA | DC2 | Western Cape |
| 9 | 1/27/2016 | NA | 2/4/2016 | NA | NA | DC36 | Limpopo |
| 9 | 1/2/2016 | 1/5/2016 | 1/10/2016 | 6.0815 | 7.7667 | DC45 | Northern Cape |
| 9 | 11/28/2016 | NA | 12/6/2016 | NA | NA | DC45 | Northern Cape |
| 9 | 2/12/2016 | NA | 2/20/2016 | NA | NA | DC47 | Limpopo |
| 9 | 12/29/2015 | 1/3/2016 | 1/6/2016 | 4.1138 | 6.45 | DC6 | Northern Cape |
| 9 | 12/31/2018 | NA | 1/8/2019 | NA | NA | DC8 | Northern Cape |
| 8 | 10/23/2018 | 10/24/2018 | 10/30/2018 | 2.3 | 5.75 | BUF | Eastern Cape |
| 8 | 12/30/2015 | NA | 1/6/2016 | NA | NA | DC12 | Eastern Cape |
| 8 | 12/29/2015 | NA | 1/5/2016 | NA | NA | DC1 | Western Cape |
| 8 | 10/21/2018 | NA | 10/28/2018 | NA | NA | DC1 | Western Cape |
| 8 | 2/11/2014 | 2/12/2014 | 2/18/2014 | 2.125 | 4.8 | DC29 | KwaZulu-Natal |
| 8 | 12/13/2018 | 12/13/2018 | 12/20/2018 | 3.3281 | 6.05 | DC36 | Limpopo |
| 8 | 12/16/2016 | 12/20/2016 | 12/23/2016 | 3.1063 | 5.15 | DC37 | North West |
| 8 | 1/1/2016 | NA | 1/8/2016 | NA | NA | DC38 | North West |
| 8 | 12/18/2015 | NA | 12/25/2015 | NA | NA | DC39 | North West |
| 8 | 11/30/2015 | NA | 12/7/2015 | NA | NA | DC8 | Northern Cape |
| 8 | 1/22/2019 | NA | 1/29/2019 | NA | NA | DC8 | Northern Cape |
| 8 | 1/3/2016 | NA | 1/10/2016 | NA | NA | DC9 | Northern Cape |
| 7 | 4/8/2014 | 4/14/2014 | 4/14/2014 | 1.6929 | 4.25 | BUF | Eastern Cape |
| 7 | 1/15/2016 | 1/15/2016 | 1/21/2016 | 3.3125 | 5.8125 | DC1 | Western Cape |
| 7 | 4/10/2016 | 4/16/2016 | 4/16/2016 | 3.1714 | 6.2 | DC29 | KwaZulu-Natal |
| 7 | 3/17/2017 | 3/18/2017 | 3/23/2017 | 1.2857 | 4.2 | DC29 | KwaZulu-Natal |
| 7 | 10/24/2018 | 10/24/2018 | 10/30/2018 | 3.3 | 7.6 | DC29 | KwaZulu-Natal |
| 7 | 10/22/2018 | 10/22/2018 | 10/28/2018 | 7.8037 | 10.4857 | DC2 | Western Cape |
| 7 | 12/20/2014 | 12/21/2014 | 12/26/2014 | 1.6238 | 5.9 | DC37 | North West |
| 7 | 12/18/2015 | NA | 12/24/2015 | NA | NA | DC48 | Gauteng |
| 7 | 10/24/2015 | 10/29/2015 | 10/30/2015 | 6.4393 | 8.925 | DC8 | Northern Cape |
| 6 | 5/3/2014 | NA | 5/8/2014 | NA | NA | BUF | Eastern Cape |
| 6 | 4/10/2016 | 4/11/2016 | 4/15/2016 | 0.8417 | 2.95 | BUF | Eastern Cape |
| 6 | 12/26/2016 | NA | 12/31/2016 | NA | NA | BUF | Eastern Cape |
| 6 | 12/18/2016 | NA | 12/23/2016 | NA | NA | DC22 | KwaZulu-Natal |
| 6 | 5/14/2015 | NA | 5/19/2015 | NA | NA | DC29 | KwaZulu-Natal |
| 6 | 2/19/2016 | 2/24/2016 | 2/24/2016 | 0 | 3.4 | DC29 | KwaZulu-Natal |
| 6 | 8/27/2016 | 8/29/2016 | 9/1/2016 | 6.2667 | 8 | DC29 | KwaZulu-Natal |
| 6 | 1/30/2014 | NA | 2/4/2014 | NA | NA | DC2 | Western Cape |
| 6 | 2/14/2014 | 2/17/2014 | 2/19/2014 | 2.4643 | 5.1143 | DC2 | Western Cape |
| 6 | 12/7/2015 | NA | 12/12/2015 | NA | NA | DC33 | Limpopo |
| 6 | 11/8/2015 | 11/9/2015 | 11/13/2015 | 6.2867 | 8.38 | DC36 | Limpopo |
| 6 | 4/30/2014 | 4/30/2014 | 5/5/2014 | 7.3917 | 10.55 | DC37 | North West |
| 6 | 4/15/2017 | 4/20/2017 | 4/20/2017 | 4.7444 | 7.9 | DC37 | North West |
| 6 | 5/20/2018 | 5/25/2018 | 5/25/2018 | 4.8611 | 7.8333 | DC37 | North West |
| 6 | 9/1/2018 | 9/4/2018 | 9/6/2018 | 9.4167 | 14.8333 | DC37 | North West |
| 6 | 12/22/2018 | 12/26/2018 | 12/27/2018 | 5.3833 | 8.2 | DC38 | North West |
| 6 | 10/28/2016 | NA | 11/2/2016 | NA | NA | DC39 | North West |
| 6 | 12/6/2015 | NA | 12/11/2015 | NA | NA | DC45 | Northern Cape |
| 6 | 1/14/2018 | NA | 1/19/2018 | NA | NA | DC45 | Northern Cape |
| 6 | 12/22/2018 | NA | 12/27/2018 | NA | NA | DC45 | Northern Cape |
| 6 | 10/26/2016 | 10/30/2016 | 10/31/2016 | 5.8667 | 8.4 | DC47 | Limpopo |
| 6 | 12/6/2015 | NA | 12/11/2015 | NA | NA | DC48 | Gauteng |
| 5 | 8/27/2016 | 8/28/2016 | 8/31/2016 | 5.4 | 7.45 | BUF | Eastern Cape |
| 5 | 4/27/2017 | 5/1/2017 | 5/1/2017 | 1 | 2 | BUF | Eastern Cape |
| 5 | 11/29/2019 | NA | 12/3/2019 | NA | NA | DC27 | KwaZulu-Natal |
| 5 | 10/30/2017 | 10/30/2017 | 11/3/2017 | 3.84 | 6 | DC29 | KwaZulu-Natal |
| 5 | 4/29/2019 | 4/30/2019 | 5/3/2019 | 1.08 | 2.2 | DC29 | KwaZulu-Natal |
| 5 | 10/3/2019 | NA | 10/7/2019 | NA | NA | DC29 | KwaZulu-Natal |
| 5 | 1/20/2015 | 1/20/2015 | 1/24/2015 | 6.3114 | 7.5857 | DC2 | Western Cape |
| 5 | 1/16/2016 | 1/16/2016 | 1/20/2016 | 3.6714 | 4.2571 | DC2 | Western Cape |
| 5 | 12/2/2018 | 12/2/2018 | 12/6/2018 | 5.565 | 8.6 | DC36 | Limpopo |
| 5 | 2/27/2019 | 2/27/2019 | 3/3/2019 | 2.795 | 5.075 | DC36 | Limpopo |
| 5 | 1/1/2014 | 1/2/2014 | 1/5/2014 | 3.6533 | 7.7 | DC37 | North West |
| 5 | 11/5/2014 | 11/5/2014 | 11/9/2014 | 3.71 | 4.85 | DC37 | North West |
| 5 | 5/26/2015 | NA | 5/30/2015 | NA | NA | DC37 | North West |
| 5 | 8/29/2017 | 8/31/2017 | 9/2/2017 | 5.6867 | 8.3667 | DC37 | North West |
| 5 | 8/4/2018 | NA | 8/8/2018 | NA | NA | DC37 | North West |
| 5 | 11/28/2019 | NA | 12/2/2019 | NA | NA | DC39 | North West |
| 5 | 12/17/2016 | 12/17/2016 | 12/21/2016 | 6.36 | 7.9 | DC45 | Northern Cape |
| 5 | 12/14/2018 | 12/14/2018 | 12/18/2018 | 4.4467 | 9.1333 | DC45 | Northern Cape |
| 5 | 1/5/2019 | NA | 1/9/2019 | NA | NA | DC45 | Northern Cape |
| 5 | 11/9/2015 | 11/9/2015 | 11/13/2015 | 8.2 | 12 | DC47 | Limpopo |
| 5 | 12/20/2015 | 12/22/2015 | 12/24/2015 | 4.7 | 6.3 | DC47 | Limpopo |
| 5 | 12/23/2018 | 12/26/2018 | 12/27/2018 | 4.04 | 7.6 | DC48 | Gauteng |
| 5 | 2/14/2014 | 2/15/2014 | 2/18/2014 | 1.5622 | 2.7444 | DC6 | Northern Cape |
| 5 | 1/20/2015 | 1/20/2015 | 1/24/2015 | 5.632 | 6.64 | DC6 | Northern Cape |
| 5 | 1/29/2016 | 1/29/2016 | 2/2/2016 | 4.55 | 5.95 | DC6 | Northern Cape |
| 5 | 1/20/2015 | 1/23/2015 | 1/24/2015 | 5.85 | 7.55 | DC8 | Northern Cape |
| 5 | 12/16/2016 | 12/18/2016 | 12/20/2016 | 7.405 | 9.75 | DC8 | Northern Cape |
| 5 | 2/9/2015 | NA | 2/13/2015 | NA | NA | DC9 | Northern Cape |
| 5 | 1/3/2016 | 1/6/2016 | 1/7/2016 | 3.7533 | 6.5667 | MAN | Free State |
| 4 | 7/21/2018 | 7/22/2018 | 7/24/2018 | 3.025 | 4.6 | BUF | Eastern Cape |
| 4 | 1/30/2016 | 2/1/2016 | 2/2/2016 | 4.75 | 7.15 | DC12 | Eastern Cape |
| 4 | 4/10/2014 | 4/12/2014 | 4/13/2014 | 7.8781 | 8.7 | DC1 | Western Cape |
| 4 | 1/4/2016 | 1/6/2016 | 1/7/2016 | 2.4021 | 3.15 | DC27 | KwaZulu-Natal |
| 4 | 4/23/2018 | 4/26/2018 | 4/26/2018 | 3.4 | 6.9 | DC29 | KwaZulu-Natal |
| 4 | 12/15/2018 | 12/18/2018 | 12/18/2018 | 3.075 | 6.6 | DC29 | KwaZulu-Natal |
| 4 | 1/1/2014 | 1/2/2014 | 1/4/2014 | 4.3893 | 7.5 | DC2 | Western Cape |
| 4 | 4/10/2014 | 4/12/2014 | 4/13/2014 | 8.1429 | 9.3286 | DC2 | Western Cape |
| 4 | 3/3/2016 | 3/4/2016 | 3/6/2016 | 4.1714 | 6.6571 | DC2 | Western Cape |
| 4 | 3/19/2017 | 3/20/2017 | 3/22/2017 | 7.5 | 11.0429 | DC2 | Western Cape |
| 4 | 2/12/2018 | 2/13/2018 | 2/15/2018 | 1.6179 | 3.4429 | DC2 | Western Cape |
| 4 | 2/6/2019 | 2/7/2019 | 2/9/2019 | 5.9893 | 9.7571 | DC2 | Western Cape |
| 4 | 10/25/2019 | 10/28/2019 | 10/28/2019 | 5.5167 | 9.0333 | DC33 | Limpopo |
| 4 | 2/19/2015 | 2/21/2015 | 2/22/2015 | 4.5725 | 6.68 | DC36 | Limpopo |
| 4 | 11/29/2016 | 11/29/2016 | 12/2/2016 | 3.805 | 5.98 | DC36 | Limpopo |
| 4 | 12/7/2016 | 12/7/2016 | 12/10/2016 | 2.38 | 2.86 | DC36 | Limpopo |
| 4 | 11/15/2018 | 11/17/2018 | 11/18/2018 | 4.9 | 7.15 | DC36 | Limpopo |
| 4 | 11/25/2018 | 11/28/2018 | 11/28/2018 | 2.8313 | 4.425 | DC36 | Limpopo |
| 4 | 12/24/2018 | 12/27/2018 | 12/27/2018 | 3.3813 | 5.35 | DC36 | Limpopo |
| 4 | 1/10/2019 | 1/11/2019 | 1/13/2019 | 1.5688 | 2.625 | DC36 | Limpopo |
| 4 | 10/6/2019 | 10/8/2019 | 10/9/2019 | 6.615 | 9.26 | DC36 | Limpopo |
| 4 | 4/19/2014 | 4/20/2014 | 4/22/2014 | 4.2625 | 4.9 | DC37 | North West |
| 4 | 9/8/2015 | 9/11/2015 | 9/11/2015 | 6.7 | 8.1 | DC37 | North West |
| 4 | 5/17/2016 | 5/17/2016 | 5/20/2016 | 2.2083 | 3.0333 | DC37 | North West |
| 4 | 12/31/2016 | 1/1/2017 | 1/3/2017 | 1.825 | 3 | DC37 | North West |
| 4 | 1/31/2017 | 2/2/2017 | 2/3/2017 | 0.8583 | 2.2 | DC37 | North West |
| 4 | 11/10/2015 | 11/10/2015 | 11/13/2015 | 4.1062 | 8.2 | DC38 | North West |
| 4 | 10/28/2016 | 10/30/2016 | 10/31/2016 | 5.9812 | 7.275 | DC38 | North West |
| 4 | 1/14/2019 | 1/14/2019 | 1/17/2019 | 3.6562 | 5.275 | DC38 | North West |
| 4 | 1/13/2018 | 1/14/2018 | 1/16/2018 | 5.6375 | 9.025 | DC39 | North West |
| 4 | 12/24/2018 | 12/26/2018 | 12/27/2018 | 8.95 | 10.5 | DC39 | North West |
| 4 | 1/22/2019 | 1/24/2019 | 1/25/2019 | 7.9167 | 10.8 | DC39 | North West |
| 4 | 1/4/2016 | 1/5/2016 | 1/7/2016 | 4.225 | 5.3 | DC42 | Gauteng |
| 4 | 12/24/2018 | 12/27/2018 | 12/27/2018 | 2.225 | 2.9 | DC42 | Gauteng |
| 4 | 1/7/2015 | 1/7/2015 | 1/10/2015 | 4.975 | 7.0333 | DC45 | Northern Cape |
| 4 | 2/9/2015 | 2/11/2015 | 2/12/2015 | 5.7083 | 9.1 | DC45 | Northern Cape |
| 4 | 1/4/2016 | 1/7/2016 | 1/7/2016 | 3.575 | 5.5 | DC48 | Gauteng |
| 4 | 11/15/2018 | 11/16/2018 | 11/18/2018 | 3.6 | 4.9 | DC48 | Gauteng |
| 4 | 1/13/2019 | 1/14/2019 | 1/16/2019 | 1 | 1.8 | DC48 | Gauteng |
| 4 | 12/29/2016 | 1/1/2017 | 1/1/2017 | 5.3767 | 6.27 | DC6 | Northern Cape |
| 4 | 3/19/2017 | 3/19/2017 | 3/22/2017 | 4.4625 | 7.93 | DC6 | Northern Cape |
| 4 | 10/25/2018 | 10/26/2018 | 10/28/2018 | 6.65 | 7.58 | DC6 | Northern Cape |
| 4 | 1/1/2014 | 1/2/2014 | 1/4/2014 | 5.8813 | 8.65 | DC8 | Northern Cape |
| 4 | 12/29/2016 | 12/30/2016 | 1/1/2017 | 5.3375 | 7.4 | DC8 | Northern Cape |
| 4 | 1/4/2018 | 1/4/2018 | 1/7/2018 | 6.825 | 7.375 | DC8 | Northern Cape |
| 4 | 11/11/2018 | 11/12/2018 | 11/14/2018 | 9.525 | 10.725 | DC8 | Northern Cape |
| 4 | 12/15/2018 | 12/16/2018 | 12/18/2018 | 5.8813 | 9.7 | DC8 | Northern Cape |
| 4 | 1/22/2015 | 1/23/2015 | 1/25/2015 | 6.1625 | 7.85 | DC9 | Northern Cape |
| 4 | 1/30/2016 | 2/1/2016 | 2/2/2016 | 1.6125 | 2.8 | DC9 | Northern Cape |
| 4 | 1/13/2018 | 1/13/2018 | 1/16/2018 | 5.2 | 7.3 | DC9 | Northern Cape |
| 4 | 12/24/2018 | 12/24/2018 | 12/27/2018 | 7.55 | 8.15 | DC9 | Northern Cape |
| 4 | 1/15/2019 | 1/17/2019 | 1/18/2019 | 8.8875 | 10.05 | DC9 | Northern Cape |
| 4 | 1/15/2019 | 1/18/2019 | 1/18/2019 | 4.8583 | 7.0333 | MAN | Free State |
| 4 | 11/29/2019 | 11/30/2019 | 12/2/2019 | 3.4583 | 5.9 | MAN | Free State |
| 3 | 3/21/2014 | 3/22/2014 | 3/23/2014 | 3.6333 | 6.85 | BUF | Eastern Cape |
| 3 | 5/23/2014 | 5/24/2014 | 5/25/2014 | 1.8 | 2.3 | BUF | Eastern Cape |
| 3 | 3/21/2017 | 3/22/2017 | 3/23/2017 | 2.3167 | 2.7 | BUF | Eastern Cape |
| 3 | 9/8/2019 | 9/9/2019 | 9/10/2019 | 2.8667 | 4.05 | BUF | Eastern Cape |
| 3 | 11/25/2019 | 11/25/2019 | 11/27/2019 | 1.4833 | 2.15 | BUF | Eastern Cape |
| 3 | 2/15/2014 | 2/16/2014 | 2/17/2014 | 4.3 | 5.675 | DC1 | Western Cape |
| 3 | 1/30/2016 | 1/31/2016 | 2/1/2016 | 5.9958 | 7.2375 | DC1 | Western Cape |
| 3 | 3/20/2017 | 3/20/2017 | 3/22/2017 | 7.0125 | 8.95 | DC1 | Western Cape |
| 3 | 12/6/2017 | 12/6/2017 | 12/8/2017 | 6.7833 | 7.25 | DC1 | Western Cape |
| 3 | 1/4/2016 | 1/5/2016 | 1/6/2016 | 5.5222 | 7.9333 | DC22 | KwaZulu-Natal |
| 3 | 2/24/2016 | 2/24/2016 | 2/26/2016 | 2.7556 | 4.8333 | DC22 | KwaZulu-Natal |
| 3 | 3/5/2016 | 3/7/2016 | 3/7/2016 | 4.7778 | 6.9667 | DC22 | KwaZulu-Natal |
| 3 | 2/13/2016 | 2/15/2016 | 2/15/2016 | 1.075 | 1.5 | DC27 | KwaZulu-Natal |
| 3 | 3/5/2016 | 3/7/2016 | 3/7/2016 | 1.7333 | 2.6333 | DC27 | KwaZulu-Natal |
| 3 | 10/28/2014 | 10/30/2014 | 10/30/2014 | 4.4333 | 5.6 | DC29 | KwaZulu-Natal |
| 3 | 1/24/2015 | 1/24/2015 | 1/26/2015 | 4.7 | 7.6 | DC29 | KwaZulu-Natal |
| 3 | 3/14/2015 | 3/15/2015 | 3/16/2015 | 1.3 | 1.7 | DC29 | KwaZulu-Natal |
| 3 | 11/5/2015 | 11/6/2015 | 11/7/2015 | 5.4333 | 10 | DC29 | KwaZulu-Natal |
| 3 | 3/5/2016 | 3/7/2016 | 3/7/2016 | 1.8 | 4.4 | DC29 | KwaZulu-Natal |
| 3 | 12/31/2016 | 1/1/2017 | 1/2/2017 | 3.1 | 4.4 | DC29 | KwaZulu-Natal |
| 3 | 1/22/2017 | 1/23/2017 | 1/24/2017 | 3.7667 | 6.6 | DC29 | KwaZulu-Natal |
| 3 | 11/12/2017 | 11/13/2017 | 11/14/2017 | 2.3 | 4.7 | DC29 | KwaZulu-Natal |
| 3 | 11/19/2017 | 11/20/2017 | 11/21/2017 | 4.5667 | 5.8 | DC29 | KwaZulu-Natal |
| 3 | 1/17/2018 | 1/19/2018 | 1/19/2018 | 2.2 | 3.6 | DC29 | KwaZulu-Natal |
| 3 | 2/27/2018 | 2/27/2018 | 3/1/2018 | 2.4333 | 4.2 | DC29 | KwaZulu-Natal |
| 3 | 10/9/2018 | 10/11/2018 | 10/11/2018 | 2.9667 | 4.7 | DC29 | KwaZulu-Natal |
| 3 | 10/16/2019 | 10/17/2019 | 10/18/2019 | 2.8667 | 7.1 | DC29 | KwaZulu-Natal |
| 3 | 2/25/2014 | 2/27/2014 | 2/27/2014 | 4.1571 | 7.5714 | DC2 | Western Cape |
| 3 | 12/6/2014 | 12/6/2014 | 12/8/2014 | 4.4714 | 4.9714 | DC2 | Western Cape |
| 3 | 1/28/2015 | 1/29/2015 | 1/30/2015 | 4 | 6.4286 | DC2 | Western Cape |
| 3 | 12/5/2015 | 12/7/2015 | 12/7/2015 | 3.7952 | 4.0714 | DC2 | Western Cape |
| 3 | 2/21/2017 | 2/23/2017 | 2/23/2017 | 3.3238 | 4.6857 | DC2 | Western Cape |
| 3 | 12/6/2017 | 12/8/2017 | 12/8/2017 | 7.1937 | 9.5167 | DC2 | Western Cape |
| 3 | 1/26/2019 | 1/28/2019 | 1/28/2019 | 4.7286 | 6.6 | DC2 | Western Cape |
| 3 | 11/11/2015 | 11/11/2015 | 11/13/2015 | 6.1 | 7.5 | DC33 | Limpopo |
| 3 | 12/22/2016 | 12/22/2016 | 12/24/2016 | 2.9333 | 4.45 | DC33 | Limpopo |
| 3 | 11/26/2018 | 11/28/2018 | 11/28/2018 | 6.2833 | 10.65 | DC33 | Limpopo |
| 3 | 1/11/2019 | 1/12/2019 | 1/13/2019 | 1.7833 | 3.7 | DC33 | Limpopo |
| 3 | 12/25/2019 | 12/25/2019 | 12/27/2019 | 5.8889 | 6.4 | DC33 | Limpopo |
| 3 | 1/23/2014 | 1/25/2014 | 1/25/2014 | 2.06 | 2.8 | DC36 | Limpopo |
| 3 | 12/24/2014 | 12/24/2014 | 12/26/2014 | 1.7867 | 3.48 | DC36 | Limpopo |
| 3 | 10/24/2015 | 10/26/2015 | 10/26/2015 | 4.4133 | 5.46 | DC36 | Limpopo |
| 3 | 10/31/2015 | 10/31/2015 | 11/2/2015 | 5.0067 | 8.06 | DC36 | Limpopo |
| 3 | 11/25/2015 | 11/27/2015 | 11/27/2015 | 5.0733 | 6.28 | DC36 | Limpopo |
| 3 | 11/4/2016 | 11/4/2016 | 11/6/2016 | 3.7133 | 4.48 | DC36 | Limpopo |
| 3 | 11/20/2017 | 11/21/2017 | 11/22/2017 | 3.24 | 6.22 | DC36 | Limpopo |
| 3 | 1/6/2018 | 1/6/2018 | 1/8/2018 | 7.22 | 8.9 | DC36 | Limpopo |
| 3 | 10/19/2019 | 10/21/2019 | 10/21/2019 | 7.0867 | 7.38 | DC36 | Limpopo |
| 3 | 10/26/2019 | 10/28/2019 | 10/28/2019 | 3.98 | 5.04 | DC36 | Limpopo |
| 3 | 11/6/2019 | 11/7/2019 | 11/8/2019 | 6.78 | 8.02 | DC36 | Limpopo |
| 3 | 2/11/2017 | 2/12/2017 | 2/13/2017 | 1.5778 | 2.4 | DC37 | North West |
| 3 | 4/6/2018 | 4/6/2018 | 4/8/2018 | 4.5333 | 5.1667 | DC37 | North West |
| 3 | 12/24/2019 | 12/25/2019 | 12/26/2019 | 2.3 | 4.2 | DC37 | North West |
| 3 | 12/5/2015 | 12/7/2015 | 12/7/2015 | 7.3667 | 8.625 | DC38 | North West |
| 3 | 11/15/2018 | 11/16/2018 | 11/17/2018 | 5.55 | 6.5 | DC38 | North West |
| 3 | 12/5/2015 | 12/6/2015 | 12/7/2015 | 10.8667 | 12.1 | DC39 | North West |
| 3 | 12/15/2018 | 12/16/2018 | 12/17/2018 | 5.5889 | 7.0667 | DC39 | North West |
| 3 | 1/16/2019 | 1/17/2019 | 1/18/2019 | 8.1889 | 8.7 | DC39 | North West |
| 3 | 1/17/2014 | 1/17/2014 | 1/19/2014 | 4.8778 | 7.4 | DC45 | Northern Cape |
| 3 | 1/30/2016 | 1/31/2016 | 2/1/2016 | 3.8889 | 4.7 | DC45 | Northern Cape |
| 3 | 11/28/2019 | 11/28/2019 | 11/30/2019 | 5.0222 | 6.6333 | DC45 | Northern Cape |
| 3 | 12/24/2014 | 12/24/2014 | 12/26/2014 | 3.3 | 4.8 | DC47 | Limpopo |
| 3 | 10/31/2015 | 10/31/2015 | 11/2/2015 | 5.7667 | 9.3 | DC47 | Limpopo |
| 3 | 1/28/2016 | 1/30/2016 | 1/30/2016 | 4.7333 | 6.3 | DC47 | Limpopo |
| 3 | 11/4/2016 | 11/5/2016 | 11/6/2016 | 3.4333 | 3.7 | DC47 | Limpopo |
| 3 | 1/11/2019 | 1/12/2019 | 1/13/2019 | 3.6333 | 4.4 | DC47 | Limpopo |
| 3 | 12/25/2019 | 12/25/2019 | 12/27/2019 | 4.4667 | 4.7 | DC47 | Limpopo |
| 3 | 11/10/2015 | 11/10/2015 | 11/12/2015 | 4.9333 | 9.1 | DC48 | Gauteng |
| 3 | 10/29/2016 | 10/31/2016 | 10/31/2016 | 5.6 | 8.9 | DC48 | Gauteng |
| 3 | 1/1/2014 | NA | 1/3/2014 | NA | NA | DC6 | Northern Cape |
| 3 | 1/10/2016 | 1/10/2016 | 1/12/2016 | 3.11 | 4.85 | DC6 | Northern Cape |
| 3 | 12/6/2017 | 12/6/2017 | 12/8/2017 | 3.4633 | 4.14 | DC6 | Northern Cape |
| 3 | 1/11/2018 | 1/12/2018 | 1/13/2018 | 4.3967 | 5.63 | DC6 | Northern Cape |
| 3 | 2/5/2019 | 2/6/2019 | 2/7/2019 | 5.2867 | 6.04 | DC6 | Northern Cape |
| 3 | 3/9/2015 | 3/9/2015 | 3/11/2015 | 6.1444 | 7.6333 | DC8 | Northern Cape |
| 3 | 12/18/2015 | 12/18/2015 | 12/20/2015 | 7.825 | 10.875 | DC8 | Northern Cape |
| 3 | 1/30/2016 | 1/30/2016 | 2/1/2016 | 6.85 | 8.3 | DC8 | Northern Cape |
| 3 | 2/9/2016 | 2/10/2016 | 2/11/2016 | 7.0583 | 7.975 | DC8 | Northern Cape |
| 3 | 2/8/2017 | 2/9/2017 | 2/10/2017 | 3.9333 | 4.85 | DC8 | Northern Cape |
| 3 | 3/4/2018 | 3/5/2018 | 3/6/2018 | 4.9 | 7.2667 | DC8 | Northern Cape |
| 3 | 10/17/2019 | 10/19/2019 | 10/19/2019 | 4.3333 | 6 | DC8 | Northern Cape |
| 3 | 11/28/2019 | 11/28/2019 | 11/30/2019 | 4.3583 | 7.75 | DC8 | Northern Cape |
| 3 | 12/18/2016 | 12/20/2016 | 12/20/2016 | 4.45 | 6.15 | DC9 | Northern Cape |
| 3 | 11/12/2018 | 11/14/2018 | 11/14/2018 | 7.4667 | 9.95 | DC9 | Northern Cape |
| 3 | 1/7/2019 | 1/7/2019 | 1/9/2019 | 5.8833 | 6.15 | DC9 | Northern Cape |
| 3 | 11/28/2019 | 11/30/2019 | 11/30/2019 | 6.55 | 7.75 | DC9 | Northern Cape |
| 3 | 12/18/2015 | 12/18/2015 | 12/20/2015 | 5.0778 | 6.2 | MAN | Free State |
| 3 | 12/15/2018 | 12/16/2018 | 12/17/2018 | 2.9111 | 5.6667 | MAN | Free State |
| 3 | 1/22/2019 | 1/23/2019 | 1/24/2019 | 6.2556 | 8.0333 | MAN | Free State |
| 3 | 11/10/2015 | 11/10/2015 | 11/12/2015 | 3.66 | 4.88 | TSH | Gauteng |
| 3 | 10/29/2016 | 10/30/2016 | 10/31/2016 | 2.8033 | 3.395 | TSH | Gauteng |
| 2 | 1/22/2014 | 1/23/2014 | 1/23/2014 | 1.95 | 3.25 | BUF | Eastern Cape |
| 2 | 1/27/2014 | 1/27/2014 | 1/28/2014 | 1.575 | 2.35 | BUF | Eastern Cape |
| 2 | 7/16/2014 | 7/16/2014 | 7/17/2014 | 4.975 | 5.95 | BUF | Eastern Cape |
| 2 | 8/12/2014 | 8/13/2014 | 8/13/2014 | 3.3 | 6.05 | BUF | Eastern Cape |
| 2 | 8/18/2014 | 8/19/2014 | 8/19/2014 | 2.4 | 3.3 | BUF | Eastern Cape |
| 2 | 10/22/2014 | 10/22/2014 | 10/23/2014 | 1.8 | 1.9 | BUF | Eastern Cape |
| 2 | 1/15/2015 | 1/16/2015 | 1/16/2015 | 1.525 | 1.6 | BUF | Eastern Cape |
| 2 | 1/23/2015 | 1/24/2015 | 1/24/2015 | 1.7 | 2.4 | BUF | Eastern Cape |
| 2 | 2/23/2015 | 2/23/2015 | 2/24/2015 | 0.575 | 1 | BUF | Eastern Cape |
| 2 | 3/3/2015 | 3/4/2015 | 3/4/2015 | 3.625 | 3.95 | BUF | Eastern Cape |
| 2 | 5/20/2015 | 5/20/2015 | 5/21/2015 | 0.85 | 1.35 | BUF | Eastern Cape |
| 2 | 11/5/2015 | 11/5/2015 | 11/6/2015 | 4.4 | 6.75 | BUF | Eastern Cape |
| 2 | 12/27/2015 | 12/27/2015 | 12/28/2015 | 2.1 | 2.2 | BUF | Eastern Cape |
| 2 | 1/29/2016 | 1/29/2016 | 1/30/2016 | 1.6 | 1.85 | BUF | Eastern Cape |
| 2 | 2/23/2016 | 2/23/2016 | 2/24/2016 | 1.425 | 2.25 | BUF | Eastern Cape |
| 2 | 2/28/2016 | 2/29/2016 | 2/29/2016 | 1.775 | 2.8 | BUF | Eastern Cape |
| 2 | 5/31/2016 | 6/1/2016 | 6/1/2016 | 0.825 | 1.35 | BUF | Eastern Cape |
| 2 | 12/17/2016 | 12/18/2016 | 12/18/2016 | 2.4 | 2.95 | BUF | Eastern Cape |
| 2 | 1/27/2017 | 1/28/2017 | 1/28/2017 | 0.575 | 0.75 | BUF | Eastern Cape |
| 2 | 3/30/2017 | 3/30/2017 | 3/31/2017 | 3.325 | 6.05 | BUF | Eastern Cape |
| 2 | 5/23/2017 | 5/23/2017 | 5/24/2017 | 3.7 | 7.2 | BUF | Eastern Cape |
| 2 | 5/30/2017 | 5/30/2017 | 5/31/2017 | 2.075 | 2.4 | BUF | Eastern Cape |
| 2 | 6/6/2017 | 6/6/2017 | 6/7/2017 | 3.475 | 4.35 | BUF | Eastern Cape |
| 2 | 7/6/2017 | 7/6/2017 | 7/7/2017 | 3.325 | 3.7 | BUF | Eastern Cape |
| 2 | 9/4/2017 | 9/5/2017 | 9/5/2017 | 6.525 | 7.1 | BUF | Eastern Cape |
| 2 | 10/22/2017 | 10/23/2017 | 10/23/2017 | 4.75 | 8.1 | BUF | Eastern Cape |
| 2 | 10/31/2017 | 10/31/2017 | 11/1/2017 | 2.1 | 3.1 | BUF | Eastern Cape |
| 2 | 1/11/2018 | 1/11/2018 | 1/12/2018 | 1.475 | 1.55 | BUF | Eastern Cape |
| 2 | 2/8/2018 | 2/9/2018 | 2/9/2018 | 1.85 | 3.05 | BUF | Eastern Cape |
| 2 | 3/27/2018 | 3/28/2018 | 3/28/2018 | 0.95 | 1.25 | BUF | Eastern Cape |
| 2 | 5/1/2018 | 5/2/2018 | 5/2/2018 | 4.225 | 4.8 | BUF | Eastern Cape |
| 2 | 6/13/2018 | 6/13/2018 | 6/14/2018 | 0.75 | 1.4 | BUF | Eastern Cape |
| 2 | 9/30/2018 | 9/30/2018 | 10/1/2018 | 3.7 | 4.5 | BUF | Eastern Cape |
| 2 | 4/29/2019 | 4/29/2019 | 4/30/2019 | 4.1 | 5.05 | BUF | Eastern Cape |
| 2 | 5/19/2019 | 5/19/2019 | 5/20/2019 | 7.75 | 8 | BUF | Eastern Cape |
| 2 | 7/27/2019 | 7/27/2019 | 7/28/2019 | 3.175 | 4.85 | BUF | Eastern Cape |
| 2 | 10/16/2019 | 10/17/2019 | 10/17/2019 | 4.775 | 6.8 | BUF | Eastern Cape |
| 2 | 11/5/2019 | 11/5/2019 | 11/6/2019 | 0.6 | 0.65 | BUF | Eastern Cape |
| 2 | 3/23/2014 | 3/23/2014 | 3/24/2014 | 2.6 | 2.6 | DC12 | Eastern Cape |
| 2 | 1/22/2017 | 1/22/2017 | 1/23/2017 | 7 | 10.05 | DC12 | Eastern Cape |
| 2 | 12/17/2018 | 12/18/2018 | 12/18/2018 | 5.1 | 6.65 | DC12 | Eastern Cape |
| 2 | 2/25/2014 | 2/26/2014 | 2/26/2014 | 4.175 | 5.1375 | DC1 | Western Cape |
| 2 | 3/2/2015 | 3/3/2015 | 3/3/2015 | 9.5312 | 10.9875 | DC1 | Western Cape |
| 2 | 2/12/2018 | 2/12/2018 | 2/13/2018 | 3.567 | 3.675 | DC1 | Western Cape |
| 2 | 1/27/2019 | 1/28/2019 | 1/28/2019 | 7.6188 | 8.2125 | DC1 | Western Cape |
| 2 | 2/6/2019 | 2/6/2019 | 2/7/2019 | 9.3911 | 9.825 | DC1 | Western Cape |
| 2 | 1/23/2014 | 1/24/2014 | 1/24/2014 | 2.5667 | 2.5667 | DC22 | KwaZulu-Natal |
| 2 | 1/28/2014 | 1/28/2014 | 1/29/2014 | 1.1 | 1.5333 | DC22 | KwaZulu-Natal |
| 2 | 2/11/2014 | 2/12/2014 | 2/12/2014 | 4.1667 | 5.8 | DC22 | KwaZulu-Natal |
| 2 | 12/1/2015 | 12/1/2015 | 12/2/2015 | 5.2 | 9.6 | DC22 | KwaZulu-Natal |
| 2 | 12/18/2015 | 12/18/2015 | 12/19/2015 | 5.4833 | 5.7 | DC22 | KwaZulu-Natal |
| 2 | 12/8/2016 | 12/8/2016 | 12/9/2016 | 4.175 | 4.65 | DC22 | KwaZulu-Natal |
| 2 | 1/1/2017 | 1/2/2017 | 1/2/2017 | 4.25 | 5.85 | DC22 | KwaZulu-Natal |
| 2 | 1/23/2017 | 1/23/2017 | 1/24/2017 | 3.2 | 4.75 | DC22 | KwaZulu-Natal |
| 2 | 2/1/2017 | 2/1/2017 | 2/2/2017 | 5 | 5.15 | DC22 | KwaZulu-Natal |
| 2 | 4/4/2017 | 4/4/2017 | 4/5/2017 | 7.1 | 7.95 | DC22 | KwaZulu-Natal |
| 2 | 12/6/2018 | 12/6/2018 | 12/7/2018 | 6.5 | 6.9667 | DC22 | KwaZulu-Natal |
| 2 | 12/17/2018 | 12/17/2018 | 12/18/2018 | 6.05 | 6.3 | DC22 | KwaZulu-Natal |
| 2 | 11/29/2019 | 11/30/2019 | 11/30/2019 | 6.9333 | 7 | DC22 | KwaZulu-Natal |
| 2 | 12/24/2019 | 12/24/2019 | 12/25/2019 | 3.3667 | 5.8667 | DC22 | KwaZulu-Natal |
| 2 | 12/20/2014 | 12/20/2014 | 12/21/2014 | 0.2125 | 0.275 | DC27 | KwaZulu-Natal |
| 2 | 1/4/2015 | 1/5/2015 | 1/5/2015 | 1.225 | 1.75 | DC27 | KwaZulu-Natal |
| 2 | 2/10/2015 | 2/11/2015 | 2/11/2015 | 0.2875 | 0.425 | DC27 | KwaZulu-Natal |
| 2 | 10/7/2015 | 10/7/2015 | 10/8/2015 | 2.925 | 4.15 | DC27 | KwaZulu-Natal |
| 2 | 10/31/2015 | 10/31/2015 | 11/1/2015 | 2.125 | 4.125 | DC27 | KwaZulu-Natal |
| 2 | 12/18/2015 | 12/19/2015 | 12/19/2015 | 2.0167 | 2.0333 | DC27 | KwaZulu-Natal |
| 2 | 1/23/2016 | 1/24/2016 | 1/24/2016 | 0.55 | 1.075 | DC27 | KwaZulu-Natal |
| 2 | 2/2/2016 | 2/2/2016 | 2/3/2016 | 1.7167 | 2.8333 | DC27 | KwaZulu-Natal |
| 2 | 2/19/2016 | 2/20/2016 | 2/20/2016 | 3.975 | 6.225 | DC27 | KwaZulu-Natal |
| 2 | 12/8/2016 | 12/9/2016 | 12/9/2016 | 1.4875 | 2.5 | DC27 | KwaZulu-Natal |
| 2 | 2/11/2017 | 2/11/2017 | 2/12/2017 | 1.1 | 1.125 | DC27 | KwaZulu-Natal |
| 2 | 11/6/2019 | 11/7/2019 | 11/7/2019 | 1.8 | 2.975 | DC27 | KwaZulu-Natal |
| 2 | 1/23/2014 | 1/24/2014 | 1/24/2014 | 3.55 | 3.8 | DC29 | KwaZulu-Natal |
| 2 | 1/28/2014 | 1/28/2014 | 1/29/2014 | 0.75 | 1.1 | DC29 | KwaZulu-Natal |
| 2 | 3/14/2014 | 3/15/2014 | 3/15/2014 | 1.15 | 1.4 | DC29 | KwaZulu-Natal |
| 2 | 3/22/2014 | 3/23/2014 | 3/23/2014 | 2.1 | 2.7 | DC29 | KwaZulu-Natal |
| 2 | 3/31/2014 | 4/1/2014 | 4/1/2014 | 3.45 | 3.6 | DC29 | KwaZulu-Natal |
| 2 | 4/11/2014 | 4/12/2014 | 4/12/2014 | 4.75 | 5.8 | DC29 | KwaZulu-Natal |
| 2 | 9/23/2014 | 9/23/2014 | 9/24/2014 | 2.2 | 4.2 | DC29 | KwaZulu-Natal |
| 2 | 10/6/2014 | 10/7/2014 | 10/7/2014 | 9.95 | 12 | DC29 | KwaZulu-Natal |
| 2 | 1/12/2015 | 1/12/2015 | 1/13/2015 | 2.2 | 4.2 | DC29 | KwaZulu-Natal |
| 2 | 2/18/2015 | 2/19/2015 | 2/19/2015 | 2.25 | 4.3 | DC29 | KwaZulu-Natal |
| 2 | 3/24/2015 | 3/24/2015 | 3/25/2015 | 1 | 1.5 | DC29 | KwaZulu-Natal |
| 2 | 4/13/2015 | 4/14/2015 | 4/14/2015 | 3.8 | 4.4 | DC29 | KwaZulu-Natal |
| 2 | 4/25/2015 | 4/26/2015 | 4/26/2015 | 1.4 | 1.8 | DC29 | KwaZulu-Natal |
| 2 | 7/11/2015 | 7/12/2015 | 7/12/2015 | 2.25 | 4.4 | DC29 | KwaZulu-Natal |
| 2 | 9/24/2015 | 9/25/2015 | 9/25/2015 | 1.45 | 2.6 | DC29 | KwaZulu-Natal |
| 2 | 10/12/2015 | 10/13/2015 | 10/13/2015 | 8 | 10 | DC29 | KwaZulu-Natal |
| 2 | 10/20/2015 | 10/21/2015 | 10/21/2015 | 3.85 | 4.7 | DC29 | KwaZulu-Natal |
| 2 | 1/4/2016 | 1/5/2016 | 1/5/2016 | 4.25 | 6.6 | DC29 | KwaZulu-Natal |
| 2 | 2/13/2016 | 2/14/2016 | 2/14/2016 | 3.95 | 6.1 | DC29 | KwaZulu-Natal |
| 2 | 3/26/2016 | 3/27/2016 | 3/27/2016 | 3.9 | 5.2 | DC29 | KwaZulu-Natal |
| 2 | 9/15/2016 | 9/15/2016 | 9/16/2016 | 2.75 | 3.3 | DC29 | KwaZulu-Natal |
| 2 | 9/26/2016 | 9/27/2016 | 9/27/2016 | 2.95 | 3.8 | DC29 | KwaZulu-Natal |
| 2 | 12/23/2016 | 12/23/2016 | 12/24/2016 | 6.6 | 7.2 | DC29 | KwaZulu-Natal |
| 2 | 2/1/2017 | 2/2/2017 | 2/2/2017 | 2.65 | 4.8 | DC29 | KwaZulu-Natal |
| 2 | 2/28/2017 | 3/1/2017 | 3/1/2017 | 0.75 | 1 | DC29 | KwaZulu-Natal |
| 2 | 3/30/2017 | 3/31/2017 | 3/31/2017 | 3.8 | 6 | DC29 | KwaZulu-Natal |
| 2 | 4/4/2017 | 4/4/2017 | 4/5/2017 | 2.65 | 5.2 | DC29 | KwaZulu-Natal |
| 2 | 5/5/2017 | 5/5/2017 | 5/6/2017 | 3.5 | 3.7 | DC29 | KwaZulu-Natal |
| 2 | 9/21/2017 | 9/22/2017 | 9/22/2017 | 3.5 | 4.6 | DC29 | KwaZulu-Natal |
| 2 | 1/12/2018 | 1/12/2018 | 1/13/2018 | 2.6 | 2.6 | DC29 | KwaZulu-Natal |
| 2 | 3/10/2018 | 3/10/2018 | 3/11/2018 | 3.8 | 4.3 | DC29 | KwaZulu-Natal |
| 2 | 10/1/2018 | 10/1/2018 | 10/2/2018 | 7.7 | 10.8 | DC29 | KwaZulu-Natal |
| 2 | 11/24/2018 | 11/25/2018 | 11/25/2018 | 4.5 | 5.9 | DC29 | KwaZulu-Natal |
| 2 | 11/30/2018 | 12/1/2018 | 12/1/2018 | 1.9 | 2.5 | DC29 | KwaZulu-Natal |
| 2 | 12/6/2018 | 12/7/2018 | 12/7/2018 | 4.5 | 6.7 | DC29 | KwaZulu-Natal |
| 2 | 3/6/2019 | 3/6/2019 | 3/7/2019 | 0.5 | 0.6 | DC29 | KwaZulu-Natal |
| 2 | 4/13/2019 | 4/13/2019 | 4/14/2019 | 2.65 | 2.8 | DC29 | KwaZulu-Natal |
| 2 | 5/19/2019 | 5/20/2019 | 5/20/2019 | 5.75 | 6.9 | DC29 | KwaZulu-Natal |
| 2 | 7/18/2019 | 7/18/2019 | 7/19/2019 | 7.5 | 7.7 | DC29 | KwaZulu-Natal |
| 2 | 12/17/2019 | 12/18/2019 | 12/18/2019 | 4.25 | 7.4 | DC29 | KwaZulu-Natal |
| 2 | 12/23/2019 | 12/24/2019 | 12/24/2019 | 2.85 | 4.7 | DC29 | KwaZulu-Natal |
| 2 | 1/21/2014 | 1/22/2014 | 1/22/2014 | 6.45 | 8 | DC2 | Western Cape |
| 2 | 1/6/2015 | 1/7/2015 | 1/7/2015 | 7.7714 | 10.0857 | DC2 | Western Cape |
| 2 | 3/2/2015 | 3/3/2015 | 3/3/2015 | 8.4988 | 12.6286 | DC2 | Western Cape |
| 2 | 4/9/2015 | 4/9/2015 | 4/10/2015 | 6.5857 | 7.9714 | DC2 | Western Cape |
| 2 | 10/19/2015 | 10/20/2015 | 10/20/2015 | 1.9143 | 3.1286 | DC2 | Western Cape |
| 2 | 10/27/2015 | 10/27/2015 | 10/28/2015 | 9.0857 | 10.5286 | DC2 | Western Cape |
| 2 | 11/29/2015 | 11/30/2015 | 11/30/2015 | 8.95 | 10.3571 | DC2 | Western Cape |
| 2 | 11/26/2016 | 11/27/2016 | 11/27/2016 | 7.2286 | 8.9714 | DC2 | Western Cape |
| 2 | 12/13/2016 | 12/14/2016 | 12/14/2016 | 5 | 7.3429 | DC2 | Western Cape |
| 2 | 1/17/2017 | 1/18/2017 | 1/18/2017 | 7.8786 | 9.4143 | DC2 | Western Cape |
| 2 | 1/22/2017 | 1/22/2017 | 1/23/2017 | 6.7143 | 8.8 | DC2 | Western Cape |
| 2 | 2/10/2017 | 2/10/2017 | 2/11/2017 | 4.475 | 6.7 | DC2 | Western Cape |
| 2 | 2/27/2017 | 2/27/2017 | 2/28/2017 | 9.2714 | 9.4286 | DC2 | Western Cape |
| 2 | 10/30/2017 | 10/30/2017 | 10/31/2017 | 8.1 | 9.6714 | DC2 | Western Cape |
| 2 | 12/29/2017 | 12/30/2017 | 12/30/2017 | 5.1429 | 5.3429 | DC2 | Western Cape |
| 2 | 1/3/2018 | 1/3/2018 | 1/4/2018 | 9.5357 | 10.3571 | DC2 | Western Cape |
| 2 | 1/28/2018 | 1/29/2018 | 1/29/2018 | 6.0143 | 6.5857 | DC2 | Western Cape |
| 2 | 2/7/2018 | 2/7/2018 | 2/8/2018 | 6.9571 | 7.0857 | DC2 | Western Cape |
| 2 | 2/21/2018 | 2/22/2018 | 2/22/2018 | 4.0286 | 6.3 | DC2 | Western Cape |
| 2 | 10/7/2018 | 10/8/2018 | 10/8/2018 | 6.8786 | 8.7571 | DC2 | Western Cape |
| 2 | 12/16/2018 | 12/16/2018 | 12/17/2018 | 5.6143 | 6.5 | DC2 | Western Cape |
| 2 | 3/15/2019 | 3/15/2019 | 3/16/2019 | 2.3357 | 2.7857 | DC2 | Western Cape |
| 2 | 12/12/2019 | 12/12/2019 | 12/13/2019 | 2.7143 | 3.1 | DC2 | Western Cape |
| 2 | 11/1/2015 | 11/2/2015 | 11/2/2015 | 7 | 9.2 | DC33 | Limpopo |
| 2 | 1/6/2016 | 1/7/2016 | 1/7/2016 | 7.675 | 8.55 | DC33 | Limpopo |
| 2 | 2/19/2016 | 2/20/2016 | 2/20/2016 | 4.4 | 5.5 | DC33 | Limpopo |
| 2 | 11/5/2016 | 11/5/2016 | 11/6/2016 | 3.975 | 4.45 | DC33 | Limpopo |
| 2 | 11/30/2016 | 12/1/2016 | 12/1/2016 | 3.85 | 5.85 | DC33 | Limpopo |
| 2 | 10/25/2017 | 10/25/2017 | 10/26/2017 | 6.6833 | 7.5 | DC33 | Limpopo |
| 2 | 9/18/2018 | 9/18/2018 | 9/19/2018 | 11.2 | 11.8 | DC33 | Limpopo |
| 2 | 12/18/2018 | 12/18/2018 | 12/19/2018 | 4.225 | 4.55 | DC33 | Limpopo |
| 2 | 12/26/2018 | 12/27/2018 | 12/27/2018 | 6.2 | 6.9 | DC33 | Limpopo |
| 2 | 10/20/2019 | 10/21/2019 | 10/21/2019 | 8.9833 | 10.1667 | DC33 | Limpopo |
| 2 | 12/19/2019 | 12/19/2019 | 12/20/2019 | 3.725 | 4.95 | DC33 | Limpopo |
| 2 | 12/26/2018 | 12/27/2018 | 12/27/2018 | 2.0288 | 2.6225 | DC34 | Limpopo |
| 2 | 10/12/2014 | 10/12/2014 | 10/13/2014 | 4.85 | 4.94 | DC36 | Limpopo |
| 2 | 10/30/2014 | 10/30/2014 | 10/31/2014 | 5.84 | 7.26 | DC36 | Limpopo |
| 2 | 11/9/2014 | 11/9/2014 | 11/10/2014 | 2.24 | 3.48 | DC36 | Limpopo |
| 2 | 1/26/2015 | 1/27/2015 | 1/27/2015 | 1.8 | 2.46 | DC36 | Limpopo |
| 2 | 10/16/2016 | 10/16/2016 | 10/17/2016 | 3.17 | 3.6 | DC36 | Limpopo |
| 2 | 12/22/2016 | 12/22/2016 | 12/23/2016 | 3.0125 | 3.765 | DC36 | Limpopo |
| 2 | 1/2/2017 | 1/3/2017 | 1/3/2017 | 1.96 | 2.64 | DC36 | Limpopo |
| 2 | 10/24/2017 | 10/25/2017 | 10/25/2017 | 6.09 | 7.1 | DC36 | Limpopo |
| 2 | 12/3/2017 | 12/3/2017 | 12/4/2017 | 2.38 | 4.46 | DC36 | Limpopo |
| 2 | 12/18/2017 | 12/18/2017 | 12/19/2017 | 3.12 | 4.88 | DC36 | Limpopo |
| 2 | 12/31/2017 | 1/1/2018 | 1/1/2018 | 5.68 | 6.72 | DC36 | Limpopo |
| 2 | 1/14/2018 | 1/15/2018 | 1/15/2018 | 3.97 | 4.72 | DC36 | Limpopo |
| 2 | 1/21/2018 | 1/21/2018 | 1/22/2018 | 2.22 | 2.5 | DC36 | Limpopo |
| 2 | 2/2/2018 | 2/2/2018 | 2/3/2018 | 1.71 | 2.22 | DC36 | Limpopo |
| 2 | 9/18/2018 | 9/19/2018 | 9/19/2018 | 9.66 | 9.8 | DC36 | Limpopo |
| 2 | 10/30/2018 | 10/30/2018 | 10/31/2018 | 5.55 | 7.825 | DC36 | Limpopo |
| 2 | 11/17/2019 | 11/18/2019 | 11/18/2019 | 3.675 | 3.8 | DC36 | Limpopo |
| 2 | 11/27/2019 | 11/28/2019 | 11/28/2019 | 2.37 | 4.06 | DC36 | Limpopo |
| 2 | 12/2/2019 | 12/2/2019 | 12/3/2019 | 5.11 | 5.36 | DC36 | Limpopo |
| 2 | 3/23/2014 | 3/24/2014 | 3/24/2014 | 0.525 | 0.6 | DC37 | North West |
| 2 | 5/9/2014 | 5/10/2014 | 5/10/2014 | 5.875 | 6 | DC37 | North West |
| 2 | 8/20/2014 | 8/20/2014 | 8/21/2014 | 6.275 | 7.55 | DC37 | North West |
| 2 | 8/15/2016 | 8/15/2016 | 8/16/2016 | 5.5667 | 10.1667 | DC37 | North West |
| 2 | 8/20/2016 | 8/21/2016 | 8/21/2016 | 7.4 | 9.5333 | DC37 | North West |
| 2 | 3/1/2017 | 3/1/2017 | 3/2/2017 | 2.1167 | 3.6333 | DC37 | North West |
| 2 | 5/9/2018 | 5/10/2018 | 5/10/2018 | 8.4833 | 8.6 | DC37 | North West |
| 2 | 2/6/2019 | 2/7/2019 | 2/7/2019 | 0.725 | 1.2 | DC37 | North West |
| 2 | 12/30/2019 | 12/30/2019 | 12/31/2019 | 2.15 | 3 | DC37 | North West |
| 2 | 10/8/2015 | 10/8/2015 | 10/9/2015 | 5.35 | 6.0833 | DC38 | North West |
| 2 | 10/31/2015 | 11/1/2015 | 11/1/2015 | 7.275 | 7.625 | DC38 | North West |
| 2 | 11/30/2016 | 12/1/2016 | 12/1/2016 | 4.775 | 4.9 | DC38 | North West |
| 2 | 2/10/2015 | 2/11/2015 | 2/11/2015 | 7.65 | 8.55 | DC39 | North West |
| 2 | 12/11/2015 | 12/11/2015 | 12/12/2015 | 6.5375 | 9.175 | DC39 | North West |
| 2 | 11/30/2016 | 12/1/2016 | 12/1/2016 | 8.1375 | 9 | DC39 | North West |
| 2 | 12/6/2015 | 12/7/2015 | 12/7/2015 | 6.6 | 8.1 | DC42 | Gauteng |
| 2 | 12/19/2015 | 12/19/2015 | 12/20/2015 | 1.3 | 2.1 | DC42 | Gauteng |
| 2 | 10/29/2016 | 10/30/2016 | 10/30/2016 | 3.05 | 3.2 | DC42 | Gauteng |
| 2 | 10/26/2019 | 10/27/2019 | 10/27/2019 | 2.95 | 3.6 | DC42 | Gauteng |
| 2 | 1/23/2015 | 1/24/2015 | 1/24/2015 | 7.7 | 7.8667 | DC45 | Northern Cape |
| 2 | 10/26/2015 | 10/27/2015 | 10/27/2015 | 1.95 | 2.8333 | DC45 | Northern Cape |
| 2 | 11/30/2015 | 11/30/2015 | 12/1/2015 | 6.9333 | 7.3 | DC45 | Northern Cape |
| 2 | 10/28/2016 | 10/28/2016 | 10/29/2016 | 8.2833 | 11.7667 | DC45 | Northern Cape |
| 2 | 11/3/2016 | 11/3/2016 | 11/4/2016 | 4.9667 | 7.1333 | DC45 | Northern Cape |
| 2 | 1/6/2018 | 1/6/2018 | 1/7/2018 | 7.2667 | 7.8333 | DC45 | Northern Cape |
| 2 | 11/17/2018 | 11/17/2018 | 11/18/2018 | 7.6667 | 7.7333 | DC45 | Northern Cape |
| 2 | 11/4/2019 | 11/4/2019 | 11/5/2019 | 7.3833 | 9.1 | DC45 | Northern Cape |
| 2 | 10/25/2015 | 10/25/2015 | 10/26/2015 | 4.4 | 5.8 | DC47 | Limpopo |
| 2 | 2/3/2016 | 2/3/2016 | 2/4/2016 | 6 | 7.4 | DC47 | Limpopo |
| 2 | 2/24/2016 | 2/24/2016 | 2/25/2016 | 2.5 | 3.8 | DC47 | Limpopo |
| 2 | 3/7/2016 | 3/7/2016 | 3/8/2016 | 3.15 | 5.4 | DC47 | Limpopo |
| 2 | 12/22/2016 | 12/22/2016 | 12/23/2016 | 1.7 | 2.2 | DC47 | Limpopo |
| 2 | 1/7/2018 | 1/8/2018 | 1/8/2018 | 8.6 | 8.9 | DC47 | Limpopo |
| 2 | 12/26/2018 | 12/26/2018 | 12/27/2018 | 2.6 | 2.9 | DC47 | Limpopo |
| 2 | 10/8/2019 | 10/8/2019 | 10/9/2019 | 6.45 | 9.6 | DC47 | Limpopo |
| 2 | 12/20/2019 | 12/20/2019 | 12/21/2019 | 2.85 | 4.3 | DC47 | Limpopo |
| 2 | 12/16/2018 | 12/16/2018 | 12/17/2018 | 3.05 | 3.2 | DC48 | Gauteng |
| 2 | 10/20/2019 | 10/20/2019 | 10/21/2019 | 6.8 | 7.5 | DC48 | Gauteng |
| 2 | 10/26/2019 | 10/27/2019 | 10/27/2019 | 3.6 | 4 | DC48 | Gauteng |
| 2 | 11/27/2019 | 11/27/2019 | 11/28/2019 | 1.15 | 1.4 | DC48 | Gauteng |
| 2 | 12/2/2019 | 12/3/2019 | 12/3/2019 | 3 | 4.5 | DC48 | Gauteng |
| 2 | 1/21/2014 | 1/21/2014 | 1/22/2014 | 2.6778 | 4.1444 | DC6 | Northern Cape |
| 2 | 2/26/2014 | 2/26/2014 | 2/27/2014 | 3.0167 | 4.8333 | DC6 | Northern Cape |
| 2 | 1/6/2015 | 1/7/2015 | 1/7/2015 | 3.965 | 4.05 | DC6 | Northern Cape |
| 2 | 10/27/2015 | 10/27/2015 | 10/28/2015 | 6.22 | 7.54 | DC6 | Northern Cape |
| 2 | 3/4/2016 | 3/4/2016 | 3/5/2016 | 5.09 | 6.93 | DC6 | Northern Cape |
| 2 | 1/17/2017 | 1/17/2017 | 1/18/2017 | 6.93 | 8.67 | DC6 | Northern Cape |
| 2 | 2/12/2018 | 2/12/2018 | 2/13/2018 | 1.895 | 3.15 | DC6 | Northern Cape |
| 2 | 11/27/2019 | 11/27/2019 | 11/28/2019 | 7.995 | 9.3 | DC6 | Northern Cape |
| 2 | 2/15/2014 | 2/16/2014 | 2/16/2014 | 2.7 | 3.225 | DC8 | Northern Cape |
| 2 | 2/11/2015 | 2/11/2015 | 2/12/2015 | 6.8 | 10.875 | DC8 | Northern Cape |
| 2 | 10/8/2015 | 10/8/2015 | 10/9/2015 | 6.6125 | 6.975 | DC8 | Northern Cape |
| 2 | 1/8/2017 | 1/8/2017 | 1/9/2017 | 3.625 | 4.65 | DC8 | Northern Cape |
| 2 | 1/18/2017 | 1/18/2017 | 1/19/2017 | 3.7375 | 3.75 | DC8 | Northern Cape |
| 2 | 11/29/2017 | 11/29/2017 | 11/30/2017 | 8.95 | 9.2 | DC8 | Northern Cape |
| 2 | 1/29/2018 | 1/29/2018 | 1/30/2018 | 5.3 | 7 | DC8 | Northern Cape |
| 2 | 2/4/2018 | 2/5/2018 | 2/5/2018 | 8.5833 | 9.4333 | DC8 | Northern Cape |
| 2 | 10/28/2018 | 10/28/2018 | 10/29/2018 | 8.625 | 10.1 | DC8 | Northern Cape |
| 2 | 11/18/2018 | 11/18/2018 | 11/19/2018 | 8.35 | 8.825 | DC8 | Northern Cape |
| 2 | 11/25/2018 | 11/25/2018 | 11/26/2018 | 4.9375 | 5.575 | DC8 | Northern Cape |
| 2 | 1/16/2019 | 1/16/2019 | 1/17/2019 | 8.625 | 8.775 | DC8 | Northern Cape |
| 2 | 3/16/2019 | 3/17/2019 | 3/17/2019 | 4.6875 | 4.725 | DC8 | Northern Cape |
| 2 | 12/30/2019 | 12/31/2019 | 12/31/2019 | 5.1 | 6.625 | DC8 | Northern Cape |
| 2 | 11/30/2015 | 12/1/2015 | 12/1/2015 | 2.425 | 2.45 | DC9 | Northern Cape |
| 2 | 12/6/2015 | 12/6/2015 | 12/7/2015 | 8.3 | 8.8 | DC9 | Northern Cape |
| 2 | 12/18/2015 | 12/18/2015 | 12/19/2015 | 3.45 | 4.35 | DC9 | Northern Cape |
| 2 | 2/6/2016 | 2/7/2016 | 2/7/2016 | 3.875 | 5.15 | DC9 | Northern Cape |
| 2 | 2/12/2016 | 2/12/2016 | 2/13/2016 | 5.325 | 7.7 | DC9 | Northern Cape |
| 2 | 10/28/2016 | 10/28/2016 | 10/29/2016 | 6.6 | 9.25 | DC9 | Northern Cape |
| 2 | 11/3/2016 | 11/3/2016 | 11/4/2016 | 4.3 | 6.5 | DC9 | Northern Cape |
| 2 | 11/27/2016 | 11/28/2016 | 11/28/2016 | 4.4 | 5.7 | DC9 | Northern Cape |
| 2 | 1/22/2019 | 1/23/2019 | 1/23/2019 | 4.35 | 4.85 | DC9 | Northern Cape |
| 2 | 10/28/2016 | 10/28/2016 | 10/29/2016 | 3.4167 | 5.4333 | MAN | Free State |
| 2 | 12/19/2016 | 12/19/2016 | 12/20/2016 | 3.8833 | 6.2 | MAN | Free State |
| 2 | 1/14/2018 | 1/14/2018 | 1/15/2018 | 2.9833 | 3.4667 | MAN | Free State |
| 2 | 11/13/2018 | 11/14/2018 | 11/14/2018 | 5.75 | 5.9333 | MAN | Free State |
| 2 | 12/25/2018 | 12/25/2018 | 12/26/2018 | 6.4 | 6.9667 | MAN | Free State |
| 2 | 1/6/2016 | 1/6/2016 | 1/7/2016 | 4.43 | 4.5 | TSH | Gauteng |
| 2 | 12/4/2018 | 12/5/2018 | 12/5/2018 | 5.545 | 6.07 | TSH | Gauteng |
| 2 | 10/20/2019 | 10/21/2019 | 10/21/2019 | 3.1367 | 3.2533 | TSH | Gauteng |
